# Supplementary material for: Atezolizumab plus stereotactic ablative radiotherapy for medically inoperable patients with early-stage non-small cell lung cancer: a multi-institutional phase I trial
Source: Nat Commun. 2023 Sep 2;14:5332. doi: 10.1038/s41467-023-40813-w (PMC10474145; doi:10.1038/s41467-023-40813-w)
Supplement: Supplementary file 1 — Supplementary Information [file 41467_2023_40813_MOESM1_ESM.pdf]

SUPPLEMENTAL TABLES

Supplemental Table 1: Normal Tissue Radiation Dose Constraints

**Serial Tissue**	**Volume**	**Volume Max (Gy)**		**Max Point Dose (Gy)**	
		4 fractions	5 fractions	4 fractions	5 fractions
Spinal Cord	<0.35 cc<br><1.2 cc	20.8 Gy	23 Gy<br>14.5	26 Gy	30 Gy
Esophagus	<5 cc	18.8 Gy	19.5 Gy	30 Gy	35 Gy
Brachial Plexus	<3 cc	23.6 Gy	27 Gy	27.2 Gy	30.5 Gy
Heart/Pericardium	<15 cc	28 Gy	32 Gy	44 Gy	50 Gy
Proximal Bronchial Tree	<4 cc	15.6 Gy	16.5 Gy	34.8 Gy	40 Gy
Skin	<10 cc	33.2 Gy	36.5 Gy	36 Gy	39.5 Gy
**Parallel Tissue**	**Critical Volume (cc)**	**Critical Volume Dose Max (Gy)**			
		4 fractions	5 fractions		
Chest wall*	<30 cc	30 Gy	30 Gy		
Combined Lung -GTV	1500 cc<br>1000 cc<br>< 10%	11.6 Gy<br>12.4 Gy<br>20 Gy	12.5 Gy<br>13.5 Gy<br>20 Gy		

* Chest wall constraint recommended when feasible

Supplemental Table 2: Flow Cytometry antibody panels

Marker	*Dye*	*Clone*	*Company*	*Cat.*	*Dil.*
T cell stimulation panel					
Live/dead	Aqua	NA	Invitrogen	L34957	1/200*
CD3	BB515	UCHT1	BD Bioscience	564465	1/80*
CD4	BuV395	L200	BD Bioscience	564107	1/80*
CD8	BuV737	SK1	BD Bioscience	612754	1/80*
FoxP3	Alexa647	259D/C7	BD Bioscience	560045	1/20*
GranB	Alexa700	GB11	BD Bioscience	560213	1/20*
IL10	BV421	JES3-9D7	BD Bioscience	566276	1/20*
TNFa	BV711	MAb11	BioLegend	502940	1/20*
CD25	PE	M-A251	BD Bioscience	560989	1/20*
Ki67	PE-Cy5	SoLA15	Invitrogen	15-5698-82	1/80*
IFNg	PE-Cy7	4S.B3	BD Bioscience	557844	1/40*
TGFb	PE-CF594	TW4-9E7	BD Bioscience	562422	1/20*
PD1	BB700	EH12.1	BD Bioscience	566461	1/40*
T cell Panel					
Live/dead	Aqua	NA	Invitrogen	L34957	1/200*
CD3	BB515	UCHT1	BD Bioscience	564465	1/80*
CD4	BuV395	L200	BD Bioscience	564107	1/80*
CD8	BuV737	SK1	BD Bioscience	612754	1/80*
CD45RO	BV711	UCHL1	BD Bioscience	563723	1/40*
CD19	BV605	SJ25C1	BD Bioscience	562653	1/80*
CD25	PECF594	M-A251	BD Bioscience	562403	1/20*
PD-1	PE	MIH4	BD Bioscience	560908	1/10*
CD278	BV785	C398.4A	BD Bioscience	567923	1/80*
Ki67	PE-Cy5	SoLA15	Invitrogen	15-5698-82	1/40*
HLADR	PE-Cy7	G46-6	BD Bioscience	560651	1/40*
TREG / NK panel					
Live/dead	Aqua	NA	Invitrogen	L34957	1/200*
CD3	BB515	UCHT1	BD Bioscience	564465	1/80*
CD4	BuV395	L200	BD Bioscience	564107	1/80*
CD8	BuV737	SK1	BD Bioscience	612754	1/80*
CD16	Alexa700	3G8	BD Bioscience	557920	1/80*
CD56	PE	MY31	BD Bioscience	566647	1/20*
CD25	PECF594	M-A251	BD Bioscience	562403	1/20*
FoxP3	Alexa647	259D/C7	BD Bioscience	560045	1/20*
TIGIT	BB700	741182	BD Bioscience	741182	1/40*
TIM3	BV711	7D3	BD Bioscience	565566	1/40*
B cell panel					
Live/dead	Aqua	NA	Invitrogen	L34957	1/200*
CD19	BV786	SJ25C1	BD Bioscience	563325	1/80*
HLADR	PE-Cy7	G46-6	BD Bioscience	560651	1/40*

IgD	BB700	IA6-2	BD Bioscience	566538	1/40*
CD27	Alexa647	M-T271	BioLegend	366434	1/40*
CD5	BV605	UCHT2	BD Bioscience	563945	1/40*
CD1d	PE	CD1d42	BD Bioscience	550255	1/20*
CD25	PECF594	M-A251	BD Bioscience	562403	1/20*
PDL1	BuV395	MIH1	BD Bioscience	740320	1/20*
IL10	BV421	JES3-9D7	BD Bioscience	566276	1/40*
MDSC / DC panel					
Live/dead	Aqua	NA	Invitrogen	L34957	1/200*
CD3	BV711	OKT3	BioLegend	317328	1/80*
CD19	BV711	SJ25C1	BD Bioscience	563036	1/80*
CD56	BV711	B159	BD Bioscience	740781	1/80*
CD14	BuV395	MϕP9	BD Bioscience	563562	1/40*
CD11b	PECY7	M1/70	BD Bioscience	561098	1/40*
CD33	BB515	WM53	BD Bioscience	564588	1/40*
HLADR	BB700	G46-6	BD Bioscience	566481	1/40*
CD11c	BV605	B-ly6	BD Bioscience	563930	1/40*
CD1c	PE	F10/21A3	BD Bioscience	564900	1/40*
CD141	BV421	1A4	BD Bioscience	563321	1/80*
CD303	BV786	V24-785	BD Bioscience	748000	1/80*
PDL1	APC	MIH1	BD Bioscience	563741	1/40*

Supplemental Table 3: All Adverse Events

Adverse Event	Total number patients	Grade	Number patients
Abdominal distension	1	1	1
Anemia	11	1	9
		2	2
Anxiety	1	1	1
Atrioventricular block	1	3	1
Chills	3	1	3
Conjunctivitis	2	2	2
Constipation	3	1	3
Dehydration	1	3	1
Dry Skin	3	1	3
Dysphagia	1	1	1
Ear disorder	1	2	1
Fever	2	1	2
Gastroesophageal Reflux	2	1	1
		2	1
Hyperthyroidism	3	1	2
		2	1
Hypothyroidism	3	1	1
		2	2
Diarrhea	5	1	5
Bronchial/upper respiratory infection	1	2	1
Increased INR	1	3	1
Increased cardiac troponins	2	1	2
Nausea	4	1	2
		2	2
Edema of limbs	5	1	3
		2	2
Fatigue	12	1	8
		2	4
Malaise	1	1	1
Edema, other	2	1	2
Chest pain	3	1	2
		2	1
Night sweats	1	1	1
Hematuria	1	1	1
Hypertension	1	3	1
Hypotension	3	1	1
		2	2
Laryngitis	1	1	1
Liver function test abnormality	5	1	4
		3	1
Lymphopenia	12	1	3
		2	6
		3	3

Leukocytosis	1	1	1
Memory impairment	1	1	1
Neutropenia	1	1	1
Thrombocytopenia	3	1	3
Decreased white blood cell count	6	1	6
Anorexia	4	1	3
		2	1
Electrolyte abnormality	13	1	7
		2	1
		3	4
		4	1
Dizziness	6	1	6
Headache	3	1	3
Dyspnea	4	1	1
		2	1
		3	2
Urinary symptoms	1	1	1
Pneumonitis	2	1	1
		2	1
Cough	7	1	4
		2	3
Puritis	2	1	2
Sore throat	1	1	1
Upper respiratory/bronchial infection	3	1	1
		2	2
Wheezing	1	1	1
Watering eyes	1	1	1
Vomiting	2	1	2
Respiratory Failure	1	4	1
Hemoptysis	1	1	1
Sepsis	1	4	1
Peripheral neuropathy	1	1	1
Nasal congestion	1	1	1
Injury to extremities	1	1	1
Flu-like symptoms	1	1	1
Weight loss	2	1	1
		3	1
Rash	6	1	4
		2	1
		3	1
Skin disorders, other	3	2	2
		3	1
Other musculoskeletal	9	1	5
		2	2
		3	2

SUPLLEMENTAL FIGURES AND FIGURE LEGENEDS

Supplemental Figure 1: Baseline PD-L1 expression.

Tissue samples were stained for PD-L1 expression by IHC using anti-human PD-L1 clone E1L3N (cell signaling). Sufficient baseline or archival tissue for staining was present for 13/20 patients. TPS percent was 0% 8/13 (62%), >1% 5/13 (31%), and >50% 1/13 (8%). Representative IHC demonstrating TPS score of 4%, 10%, and 99% are shown in the right panel. Source data are provided as a Source Data file.

Supplemental Figure 1

ID	TPS
001	00
002	04
003	NA
004	00
005	00
006	NA
007	00
008	00
009	00
010	03
011	00
012	00
013	NA
014	NA
015	99
016	2
017	NA
018	NA
019	10
020	NA

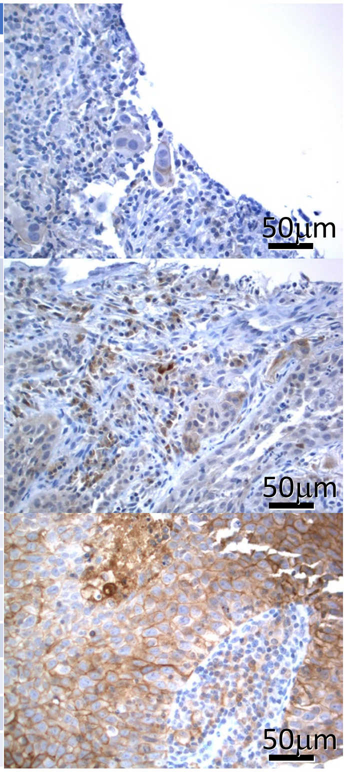

Supplemental Figure 2: Freedom from progression from the date of enrollment by the Kaplan-Meier method. **A)** Freedom from progression stratified by PD-L1 tumor proportion score (n=13; TPS<1, n=8; TPS>1, n=5). B) Freedom from progression stratified by early response to atezolizumab (RECIST v1.1, n=18; non-responder n=15; responder n=3). Source data are provided as a Source Data file.

Supplemental Figure 2

A

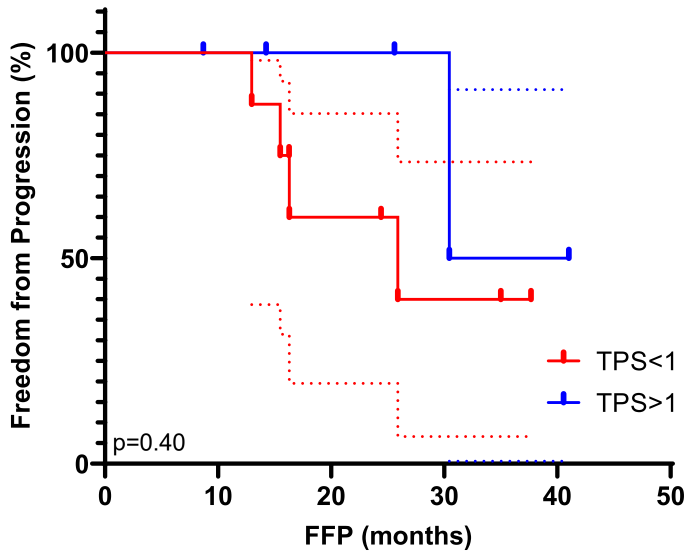

B

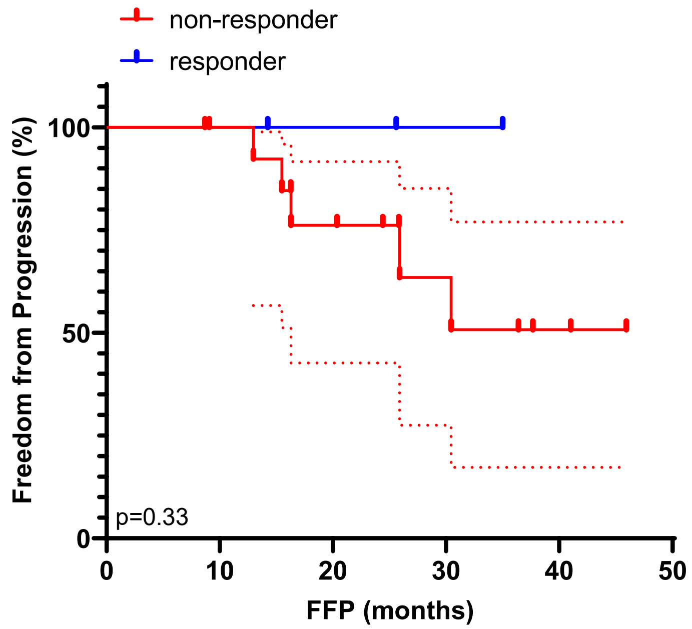

Supplemental Figure 3: Using a multi-plex quantitative immunofluorescence T cell activation panel, there was no correlation between PDL1 expression and TIL predominance or functionality in the TME including A) CD3 QIF score B) CD3 ki67 QIF and C) CD3 GZB QIF. Nine patient samples were stained (n=9; TPS<1, n=4; TPS>1, n=5). Center line represents the mean and error bars represent the standard deviation of the mean. P values determined using a two-sided t-test. Source data are provided as a Source Data file.

Supplemental Figure 3

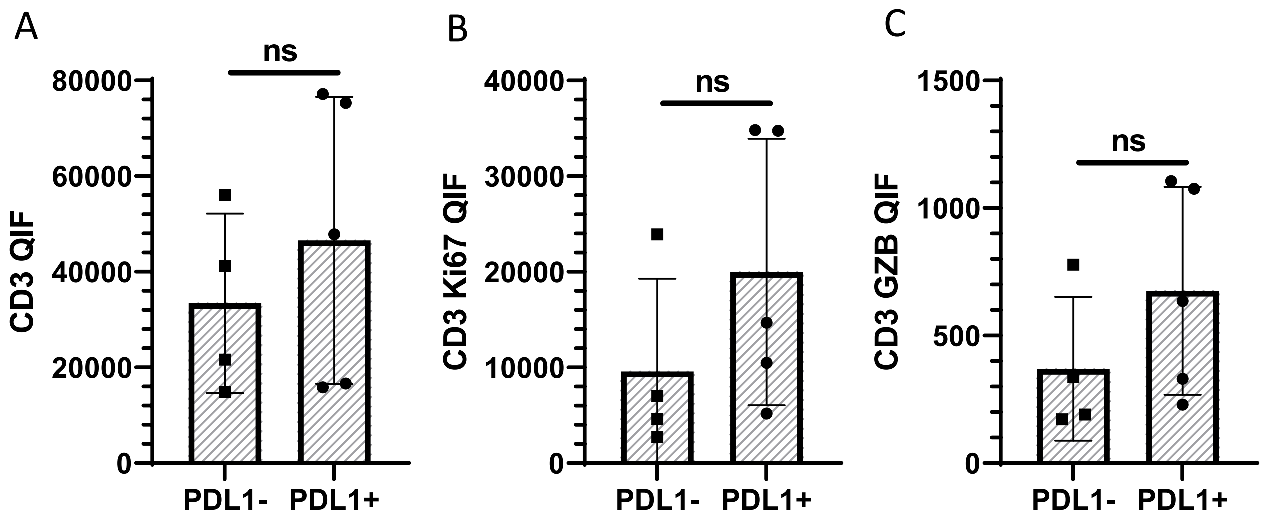

Supplemental Figure 4: Transcriptomic analysis was performed on PBMCs from a subset of patients (n=8). This analysis demonstrated no significant changes in gene expression or clustering across the examined course of therapy. A) When examining the top differentially expressed genes there was no clustering of gene expression by treatment cycle. B) Principal components analysis (PCA) also did not demonstrate grouping of gene expression by treatment cycle. C) No clustering of gene expression by treatment cycle was observed using the the BIOCARTA T cell receptor signaling gene set. D) No grouping of gene expression by treatment cycle was observed using PCA. Source data are provided as a public archive.

Supplemental Figure 4

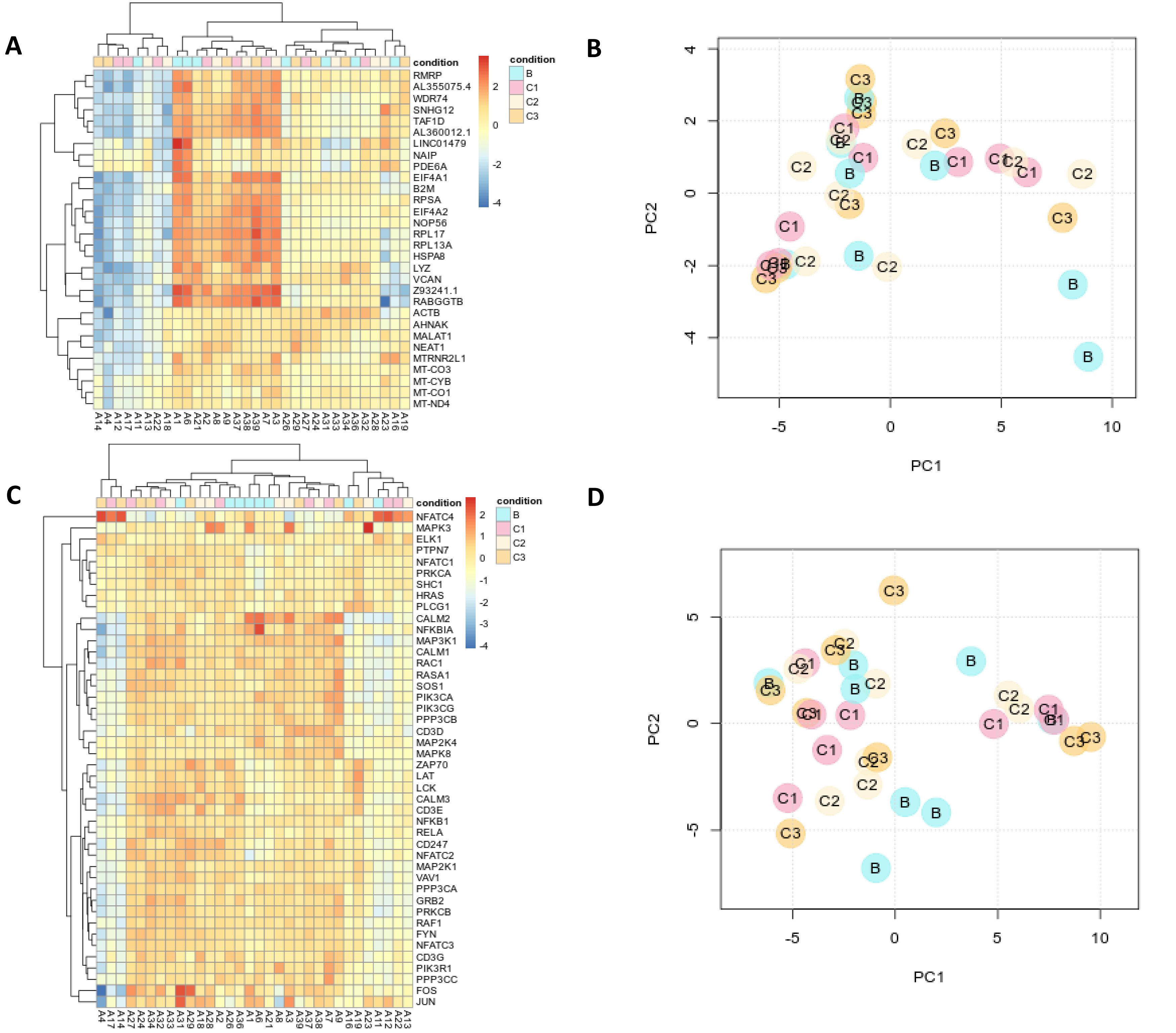

Supplemental Figure 5: Using transcriptomic analysis on pbmcs from a subset of patients (n=8) we determined a gene set, consisting largely of immune related and proliferation related genes, which clustered samples based at baseline versus post-treatment cycle 2. Source data are provided as a public archive.

Supplemental Figure 5

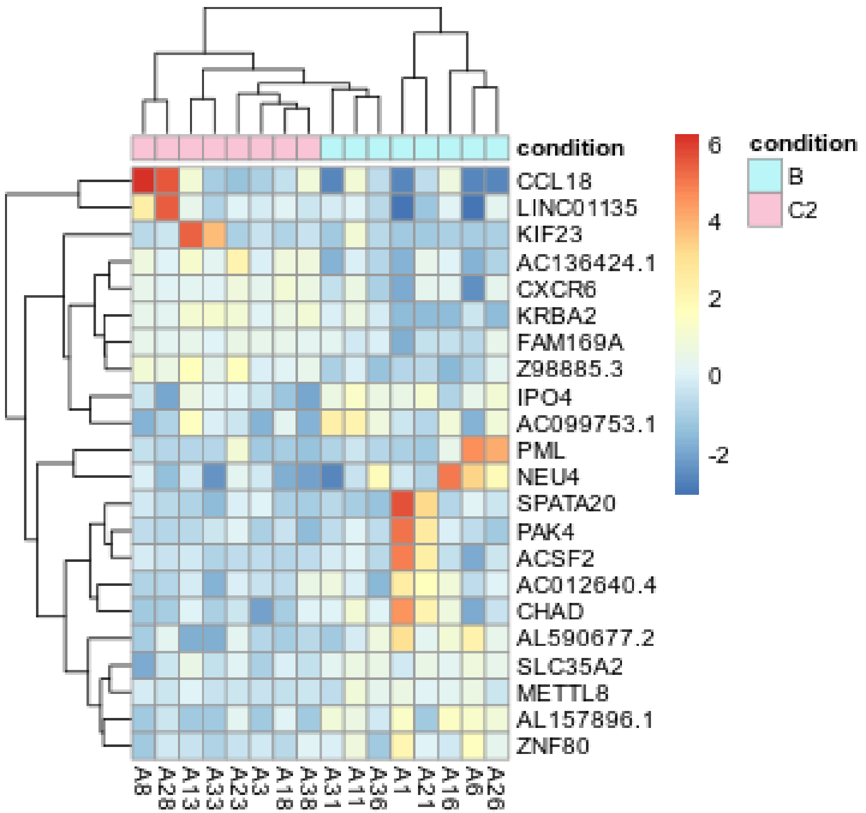

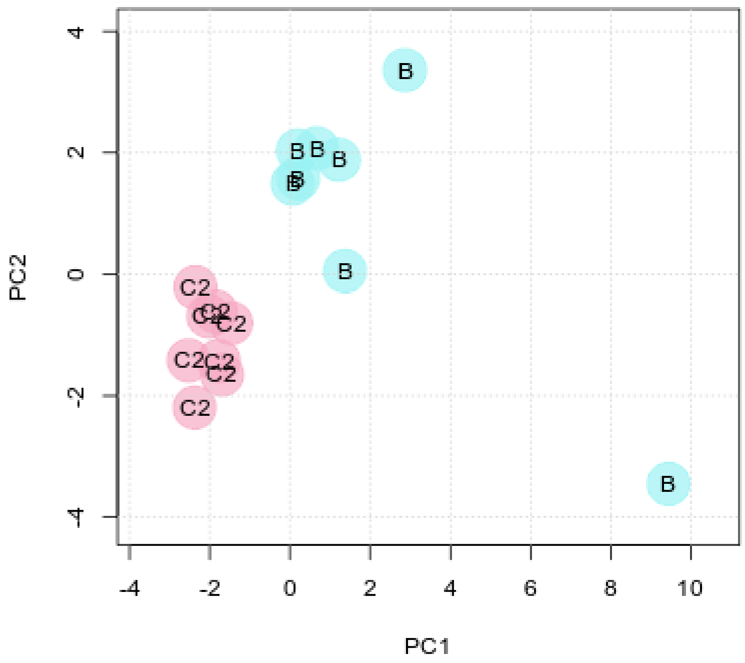

Supplemental Figure 6: Changes in PBMCs between progressors (P) and non-progressors (NP) during therapy. A) Changes in the frequency of PD1 positive among CD4+ T cells at baseline and after each of the first 3 cycles of atezolizumab. B) Changes in the frequency of PD1 positive among CD8+ T cells, C) in the mean fluorescence intensity (MFI) of Ki67on CD4+ T cells, D) in the frequency of NKT cells, E) in the frequency of TIM3 positive among NKT cells, F) in the frequency of PDL1 positive among plasmacytoid dendritic cells (pDC), G) in the MFI of PDL1 positive among pDC, and H) in the MFI of PDL1 positive among B cells. Statistical comparisons across the course of therapy were performed by ANOVA (A-C, F-H: n=16 patients at baseline; P, n=4; NP, n=12. D: P, n=3; NP, n=7. E: P, n=2, NP, n=6). Dots represent the mean and error bars represent the 95% confidence interval. Source data are provided as a Source Data file.

Supplemental Figure 6

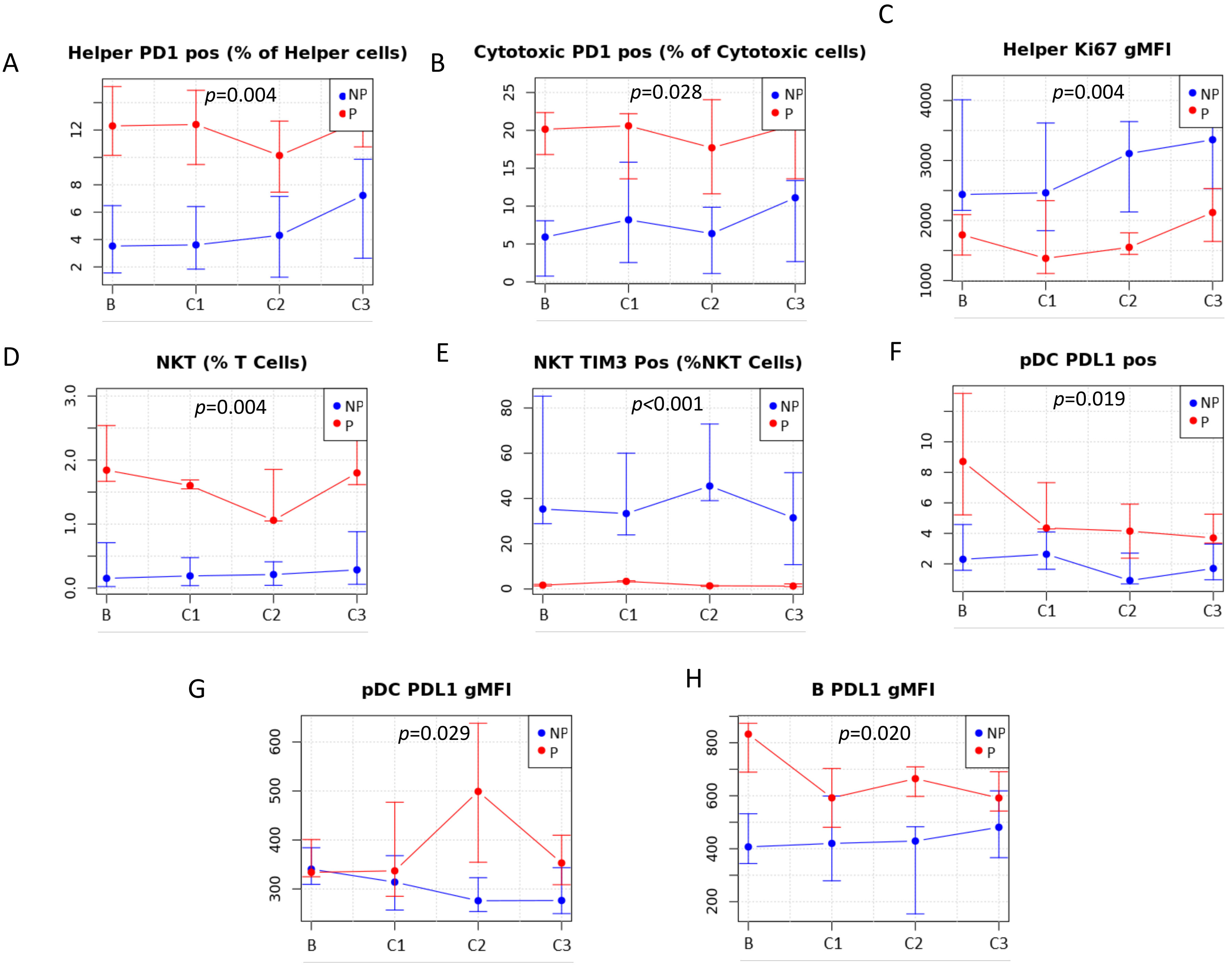

Supplemental Figure 7: Transcriptomic analysis was performed on PBMCs from a subset of patients (n=8) and demonstrated no significant changes in gene expression or clustering between progressors (P, n=3) and non-progressors (NP, n=5) at baseline. A) When examining the top differentially expressed genes there was no clustering of gene expression by progression. B) Principal components analysis (PCA) also did not demonstrate clustering or grouping of gene expression by progression. Source data are provided as a public archive.

Supplemental Figure 7

A

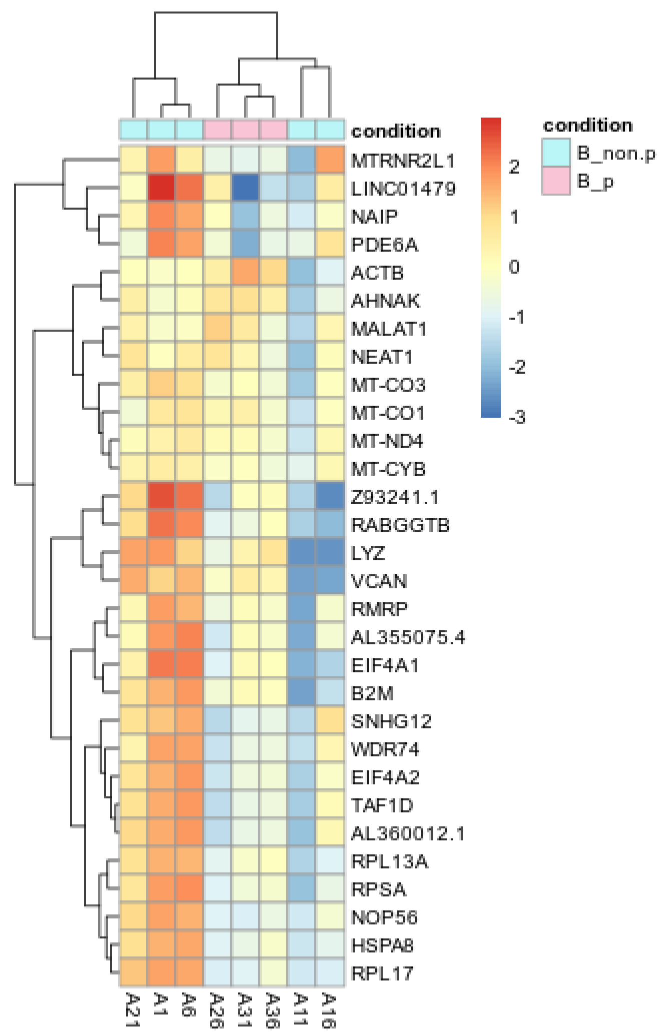

B

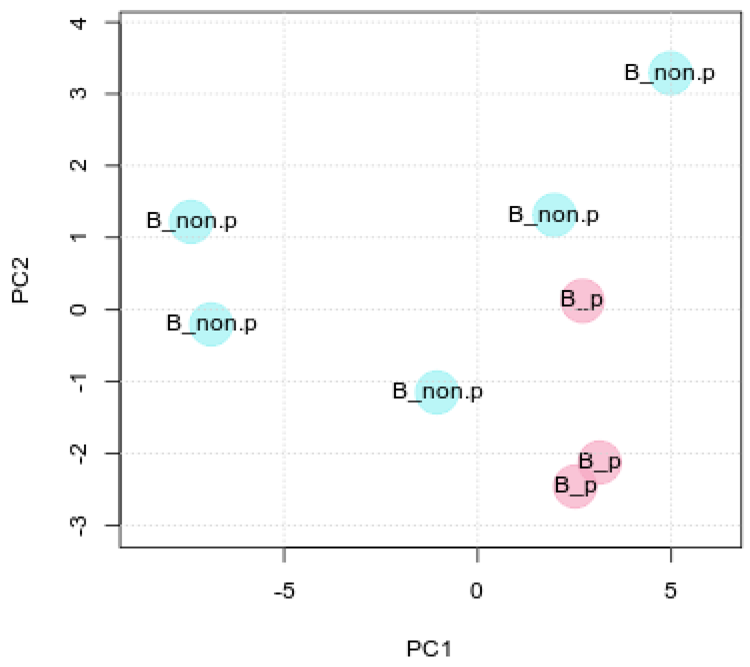

Supplemental Figure 8: Transcriptomic analysis on PBMCs from a subset of patients (n=8) demonstrated significant changes in gene expression and clustering between progressors (P, n=3) and non-progressors (NP, n=5) at baseline. A) When examining the BIOCARTA T cell receptor signaling gene set there was clustering of gene expression by progression. B) Principal components analysis (PCA) likewise demonstrates grouping of gene expression by progression. Source data are provided as a public archive.

Supplemental Figure 8

A

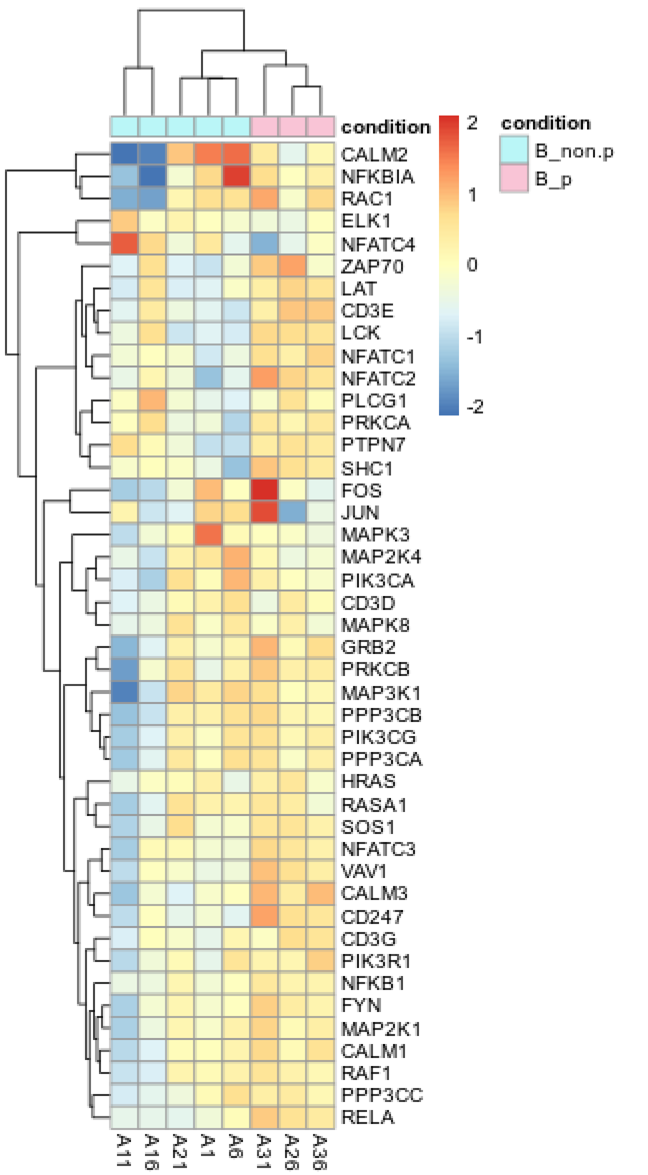

B

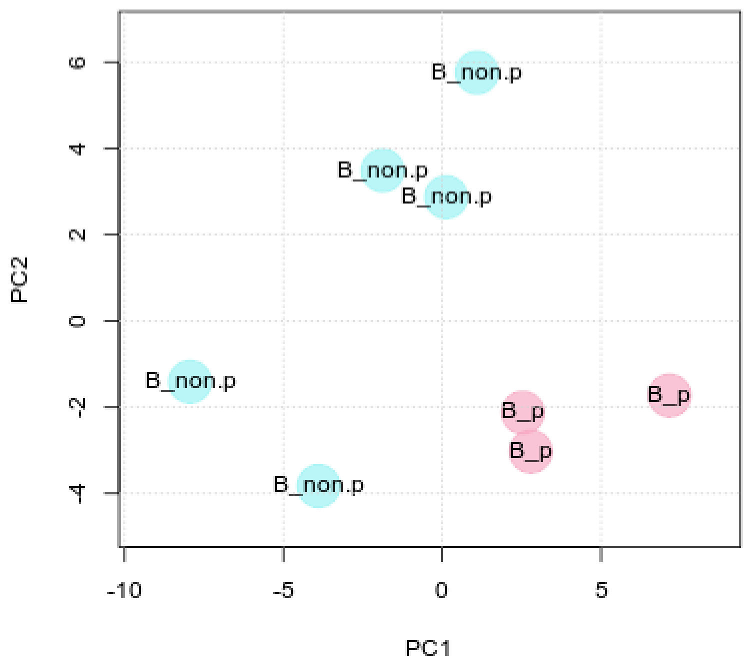

Supplemental Figure 9: Baseline PBMC Phenotype Differences in Progressors Versus Non-progressors.

A) Box and whisker plots demonstrating differences in the frequency of circulating CD4+ T cells between progressors (P, n=4) and non-progressors (NP, n=12) at baseline (n=16). B) ROC curve evaluating the ability to classify P vs. NP based on baseline levels of circulating CD4+ T cells. C) Representative flow cytometry gating for T cells of a NP (left) and P (right). D) Box and whisker plots demonstrating differences in the frequency of circulating CD4+PD1+ T cells in P vs. NP at baseline (n=16; NP, n=12; P, n=4). E) Representative flow staining for PD1 on CD4+ gated T cells in FMO negative control (top), NP (bottom left), and P (bottom right). F) ROC curve evaluating the ability to classify P vs. NP based on baseline levels of circulating CD4+PD1+ T cells. G) Box and whisker plots demonstrating differences in the frequency of circulating CD4+Tim3+ T cells in P vs. NP at baseline (n=16; NP, n=12; P, n=4). H) Representative flow staining for Tim3 on CD4+ gated T cells in FMO negative control (top), NP (bottom left), and P (bottom right). I) ROC curve evaluating the ability to classify P vs. NP based on baseline levels of circulating CD4+Tim3+ T cells. J) Box and whisker plots demonstrating differences in the frequency of circulating NKT cells in P vs. NP at baseline (n=10; NP, n=7; P, n=3). K) Representative flow staining for CD16 and CD56 double positive cells on gated T cells in FMO negative control (top), NP (bottom left), and P (bottom right). L) ROC curve evaluating the ability to classify P vs. NP based on baseline levels of circulating NKT cells. M) Box and whisker plots demonstrating differences in the frequency of circulating Tim3+ NKT cells in P vs. NP at baseline (n=8; NP, n=6; P, n=2). N) Representative flow staining forTim3 on gated NKT cells in FMO negative control (top), NP (bottom left), and P (bottom right). O) ROC curve evaluating the ability to classify P vs. NP based on baseline levels of circulating Tim3+ NKT cells. For all box and whisker plots each dot

represents the value for an individual patient, the line represents the median, the box represents the interquartile range, and the whiskers represent the spread of the data. P values determined using a two-sided t-test. Source data are provided as a Source Data file.

Supplemental Figure 9

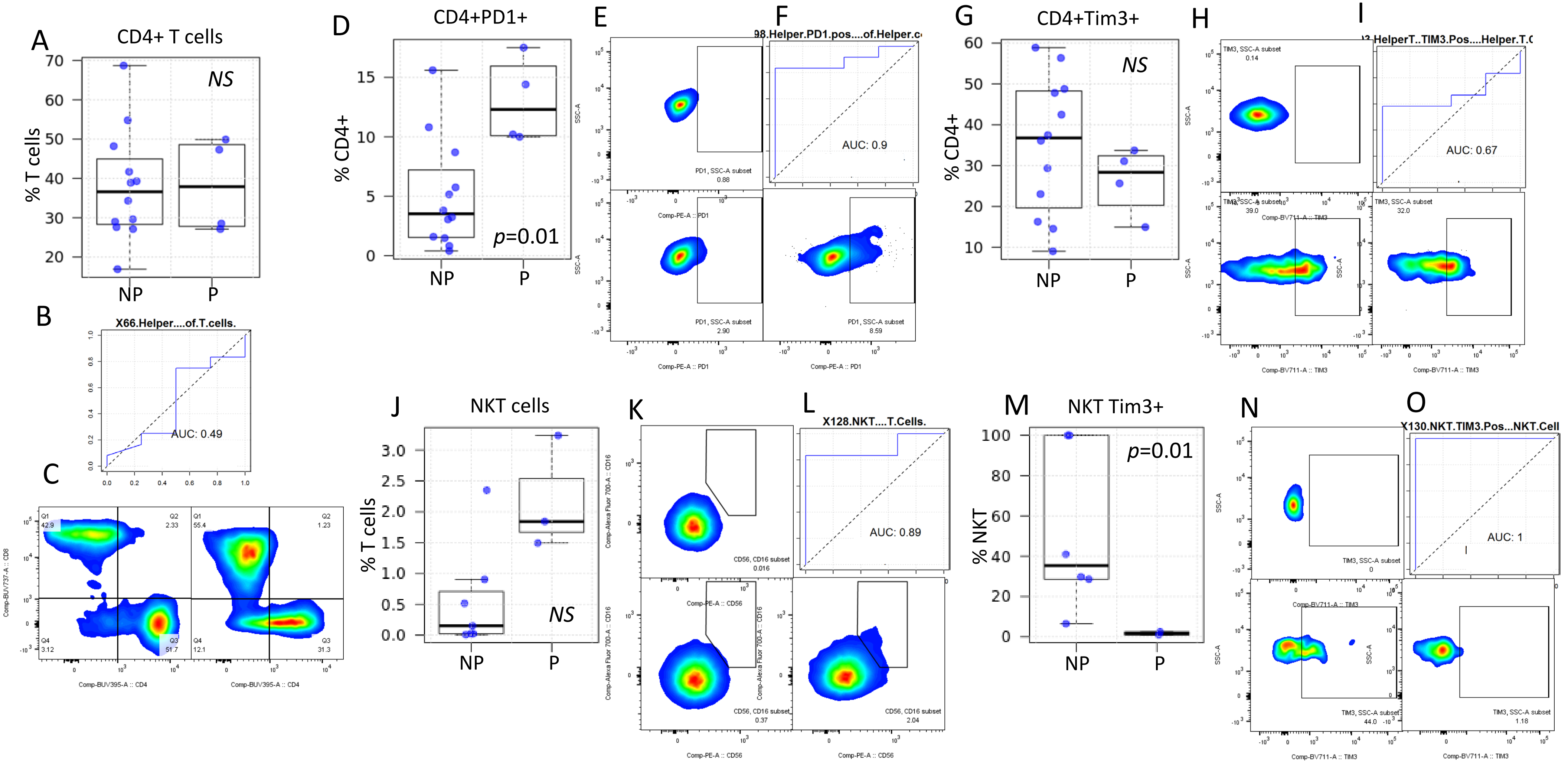

Supplementary Note: Clinical Trial Protocol

Study Title: A Phase I Trial of an Immune Checkpoint Inhibitor Plus Stereotactic Ablative Radiotherapy in Patients with Inoperable Early Stage Non-Small Cell Lung Cancer

Institutional Study #: UCDCC#258

Investigational Product: MPDL3280A (Atezolizumab)

DoD #: A18902
Genentech, Inc. #: ML29955

Multi-Principal Investigators: Karen Kelly, MD (Medical Oncology)
Megan Daly, MD (Radiation Oncology)

Investigator Contact Information:
Department of Hematology and Oncology
University of California Davis Comprehensive Cancer Center
School of Medicine
4501 X Street, Ste. 3016
Sacramento, CA 95817
Telephone: (916) 734-3735
Fax: (916) 734-7946
Email: karkelly@ucdavis.edu

Department of Radiation Oncology
University of California Davis Comprehensive Cancer Center
School of Medicine
4501 X Street, G-140
Sacramento, CA 95817
Telephone: (916) 734-5428
Email: medaly@ucdavis.edu

Co-Investigators:
Elizabeth Moore, MD (Thoracic Radiology)
Tianhong Li, MD, PhD (Medical Oncology)
David R. Gandara, MD (Medical Oncology)
Primo N. Lara, Jr., MD (Medical Oncology)
Jonathan Riess, MD, MS (Medical Oncology)
Arta Monjazeb, MD, PhD (Radiation Oncology; for correlative study assays)

Participating Centers: David Grant USAF Medical Center
101 Bodin Circle
Travis AFB, CA 94535-1809

Site PI: Amin Mirhadi, MD
Cedars-Sinai Medical Center
Samuel Oschin Comprehensive Cancer Institute
8700 Beverly Blvd
Los Angeles, CA 90048
Telephone: (310) 423-5212
Fax: (310) 659-3332

Statistician: Laurel A. Beckett, PhD
Department of Public Health Sciences
University of California Davis
Davis, CA 95616-8638

IND #: 127,072

IND Sponsor and Coordinating Center: University of California Davis Comprehensive Cancer Center

Version #:
Original/March 17, 2015
Version 1.0 / April 9, 2015
Version 2.0 / May 5, 2015
Version 3.0 / June 26, 2015
Version 4.0 / July 15, 2015
Version 5.0 / August 13, 2015
Version 6.0 / December 7, 2015
Version 7.0 / January 19, 2016
Version 8.0 / September 28, 2016
Version 9.0 / April 14, 2017
Version 10.0 / December 23, 2017
Version 11.0 / March 3, 2020

PROTOCOL SIGNATURE PAGE

Protocol Number: UCDCC#258

Protocol Title: A Phase I Trial of an Immune Checkpoint Inhibitor plus Stereotactic Ablative Radiotherapy in Patients with Inoperable Early Stage Non-Small Cell Lung Cancer

I have read this protocol and agree that it contains all necessary details for carrying out this study. I will conduct the study as outlined herein and will complete the study within the time designated, in accordance with all stipulations of the protocol and in accordance with Good Clinical Practices, local regulatory requirements, and the Declaration of Helsinki.

I will provide copies of the protocol and all pertinent information to all individuals responsible to me who assist in the conduct of this study. I will discuss this material with them to ensure that they are fully informed regarding the study agent(s) and the conduct of the study.

____
Investigator Name (print)

____
Investigator Signature

____
Date

LIST OF ABBREVIATIONS

ADCC	Antibody-dependent cell mediated cytotoxicity
AE	Adverse event
AESI	Adverse event of special interest
AFB	Air Force Base
ALK	Anaplastic lymphoma kinase
ALT	Alanine aminotransferase/serum glutamic pyruvic transaminase/SGPT
AST	Aspartate aminotransferase/serum glutamic oxaloacetic transaminase/SGOT
CNS	Central nervous system
CTCAE	Common Terminology Criteria for Adverse Events
CTV	Clinical target volume
CRF	Case Report/Record Form
CRP	C-reactive protein
DCART	Dynamic Conformal Arc Radiation Therapy
DCR	Disease control rate
DLco	diffusing capacity of the lung for carbon monoxide
DLT	Dose limiting toxicity
DoD	Department of Defense
DVH	Dose volume histogram
EBV	Epstein Barr virus
EGFR	Epidermal growth factor receptor
FACS	Fluorescence-activated cell sorting
FDA	U.S. Food and Drug Administration
FFPE	Formalin fixed paraffin embedded
FNA	Fine needle aspiration
4DCT	Four-dimensional CT
GTV	Gross tumor volume
Hr	Hour
HBsAg	Hepatitis B surface antigen
HBV	Hepatitis B virus
HCV	Hepatitis C virus
i.v.	Intravenous(ly)
ICH	International Conference on Harmonization
IEC	Independent Ethics Committee
IHC	Immunohistochemical
IMRT	Intensity modulated radiotherapy
IND	Investigational New Drug
IRB	Institutional Review Board
irAE	immune-related adverse event
irRECIST	Immune Related RECIST (irRECIST)
ITV	Internal target volume
LVEF	left ventricular ejection fraction
LFTs	Liver function tests
MRI	Magnetic resonance imaging

MTD	Maximum tolerated dose
NSCLC	Non small cell lung cancer
OCR	Office of Clinical Research
ORR	Objective response rate
OS	Overall survival
PBMC	peripheral blood mononuclear cell
PD-1	Programmed death-1
PD-L1	Programmed death-ligand 1
PFS	Progressive-free survival
PTV	Planning treatment volume
qPCR	Quantitative polymerase chain reaction
QTcF	Fridericia-corrected QT
REB	Research Ethics Board
RECIST	Response Evaluation Criteria in Solid Tumors
SAE	Serious Adverse Event
SAR	Stereotactic ablative radiotherapy
SBRT	Stereotactic body radiotherapy
SD	Stable disease
TNBK	T, B, and natural killer
TNF	Tumor necrosis factor
TSH	Thyroid-stimulating hormone
SOP	Standard Operating Procedure
VMAT	Volume Modulated Arc therapy
UCD	University of California, Davis
UCDCCC	UC Davis Comprehensive Cancer Center
ULN	Upper limit of normal
USAF	United States Air Force
USAMRMC	US Army Medical Research and Material Command
VA	Veterans Affairs

SCHEMA

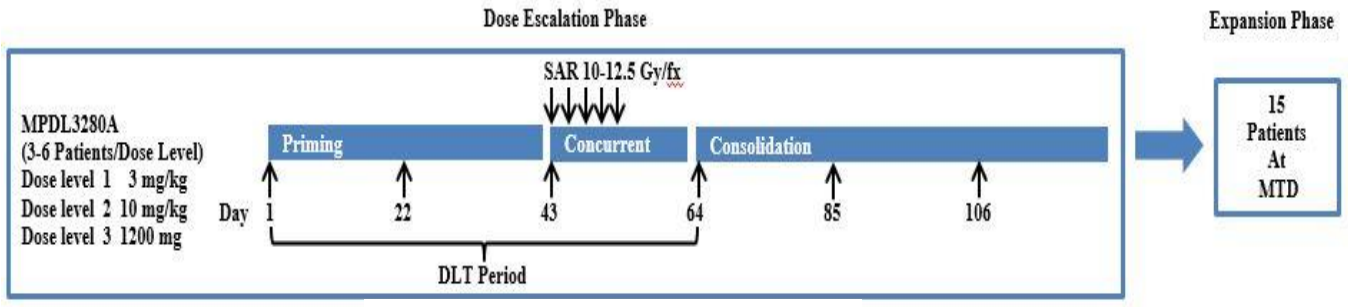
Dose Escalation Phase
Expansion Phase
SAR 10-12.5 Gy/fx
MPDL3280A
(3-6 Patients/Dose Level)
Dose level 1 3 mg/kg
Dose level 2 10 mg/kg
Dose level 3 1200 mg
Priming
Concurrent
Consolidation
Day 1
22
43
64
85
106
DLT Period
15
Patients
At
MTD

TABLE OF CONTENTS

PROTOCOL SIGNATURE PAGE 3
LIST OF ABBREVIATIONS 4
SCHEMA 6
1.0 OBJECTIVES 10
1.1 Primary Objective 10
1.2 Secondary Objectives 10
1.3 Exploratory Objectives 10
2.0 BACKGROUND 10
2.1 Medically Inoperable Early Stage Lung Cancer 10
2.2 Stereotactic Ablative Radiotherapy In Early Stage Lung Cancer 11
2.3 Cancer Immunotherapy 12
2.4 Immune Checkpoint Inhibitors 12
2.5 MPDL3280A 14
2.6 Immune Checkpoint Inhibitors Plus Stereotactic Ablative Radiotherapy 15
2.7 Biomarkers 16
2.8 Preliminary Data 17
2.9 Rationale 19
3.0 STUDY DESIGN 19
4.0 STUDY POPULATION 21
4.1 Inclusion Criteria 22
4.2 Exclusion Criteria 23
5.0 TREATMENT PLAN 24
5.1 MPDL3280A 24
5.2 SAR 25
5.2.4 Target Volumes 26
6.0 GENERAL PLAN TO MANAGE SAFETY CONCERNS 29
6.1 Management of MPDL3280A-Specific Adverse Events 30
6.2 Guidelines for Dosage Modification and Treatment Interruption or Discontinuation 30
6.3 Management of SAR- Specific Adverse Events 37
7.0 CONCOMITANT AND EXCLUDED THERAPIES 38
7.1 Concomitant Therapy 38
7.2 Excluded Therapy 39
8.0 TREATMENT DURATION 41
8.1 Duration of Follow Up 41
9.0 STUDY ASSESSMENTS AND MONITORING 41
10.0 ASSESSMENT TYPES 43
10.1 Efficacy 43

10.2 Safety 46
11.0 CORRELATIVE SCIENCE 47
11.1 Rationale 47
11.2 Study Design and Methodology 48
11.3 All Study Variables 48
12.0 DATA AND STATISTICAL ANALYSIS PLAN 49
12.1 Sample Size 49
12.2 Statistical Analysis Plan 49
12.3 Safety 50
12.4 Efficacy 50
12.5 Subject Course 50
12.6 Correlative Laboratory Markers 50
13.0 SAFETY REPORTING OF ADVERSE EVENTS 50
13.1 Assessment of Safety 50
13.2 Risks Associated with MPDL3280A 50
13.3 Adverse Events 51
13.4 Methods and Timing for Assessing and Recording Safety Variables 51
13.5 Procedures for Eliciting, Recording, and Reporting Adverse Events 53
13.6 MedWatch 3500A Reporting Guidelines 56
13.7 Additional Reporting Requirements for IND Holders 58
13.8 Study Close-Out 59
14.0 ETHICAL CONSIDERATIONS AND ADMINISTRATIVE PROCEDURES 60
14.1 Ethics and Good Clinical Practice 60
14.2 Institutional Review Board/Independent Ethics Committee 60
14.3 Study Documentation 60
14.4 Informed Consent 61
14.5 Discontinuation of Study Support 62
14.6 Amendments to the Protocol 62
14.7 Protocol Deviations 63
14.8 Quality Assurance 63
15.0 OVERSIGHT AND MONITORING 63
15.1 Data and Safety Monitoring 63
15.2 Data Safety and Monitoring (USAMRMC ORP Human Research Protections Office (HRPO) Guidelines) 64
15.3 Investigator Monitoring Guidelines 64
16.0 REFERENCES 65
17.0 APPENDICES 70
Appendix 1: Performance Status Scale 71
Appendix 2: Study Registration 72

Appendix 3: Data Submission Schedule......73
Appendix 4: Molecular Correlative Sample Handling......74
Appendix 5: Anaphylaxis Precautions......76
Appendix 6: Immune-Related Response Criteria (irRC)......77
Appendix 7: Safety Reporting Fax Cover Sheet......79
Appendix 8: Reporting Requirements and Responsibilities of the Principal Investigator to the USAMRMC ORP Human Research Protections Office (HRPO)......80

1.0 OBJECTIVES

1.1 Primary Objective

To determine the maximum tolerated dose (MTD) of MPDL3280A that can be given with SAR in patients with inoperable early stage NSCLC.

1.2 Secondary Objectives

1.2.1 To characterize the safety profile of this regimen using CTCAE v4 (Common Toxicity Criteria for Adverse Events version 4)

1.2.2 To provide preliminary efficacy data of the combination as determine by objective response rate and disease free survival using RECIST 1.1 (Response Evaluation Criteria for Solid Tumors) and Immune Related RECIST (irRECIST).

1.3 Exploratory Objectives

1.3.1 To analyze serial blood for change in cytokine signatures, FACS and immunophenotyping of peripheral blood mononuclear cells (PBMCs) and tumor infiltrating immune cells.

1.3.2 To evaluate pre and post treatment tumor tissue (if available) for PD-L1 and other immune proteins in the tumor and tumor microenvironment and for molecular profiling in a subset of patient samples.

1.3.3 To discover biomarkers of response from the data obtained in 1.3.1 and 1.3.2.

2.0 BACKGROUND

2.1 Medically Inoperable Early Stage Lung Cancer

Surgical resection is the standard of care for the treatment of early stage lung cancer with cure rates of 79% for patients with stage I disease. Unfortunately not all patients with early stage lung cancer are eligible for resection due to the presence of comorbidities such as emphysema and heart disease, common smoking related sequelae. In a review of the California Cancer Registry from 1989–2003, 18% of patients with Stage I disease did not undergo a surgical resection [1]. A similar study abstracting data from the VA Central Cancer Registry from 2003–2004 revealed that veterans with cancer have substantial comorbid illnesses [4]. The investigators reported 35% of patients with stage I/II lung cancer were unable to undergo a surgical resection. Poor health was cited as the reason in > 60% of cases. Another subpopulation of patients frequently not offered surgery or refused surgery is the elderly (defined as patients ≥65 years old) even without comorbidities. Wang and colleagues reported 40% of healthy elderly veterans did not undergo surgical resection of their early stage lung cancer solely because of advancing age [5].

In the upcoming years we can expect to see an increase the number of patients with inoperable early stage lung cancer due to two major factors. First is our aging population. Today, the life expectancy of Americans is 79.8 years and this will continue to increase. Already the median age for patients with lung cancer is 70 years old [6]. Second, is the implementation of low dose

CT (computerized tomography) screening. CT screening guidelines have already been extended to increase the screening age (from 74 years old to 80 years) and a long awaited national implementation of CT screening will be officially launched in 2015. Several centers including VA (Veterans Affairs) medical systems have already instituted CT screening programs. In the National Lung Screening Trial (NLST), 52% of the lung cancers detected by a positive screen were stage IA [7]. With an increase in the number of patients with inoperable early stage lung cancer, it is imperative that enhanced curative treatment strategies are identified.

2.2 Stereotactic Ablative Radiotherapy In Early Stage Lung Cancer

Stereotactic ablative radiotherapy (SAR), which allows precise delivery of radiation doses over 1-5 fractions to the target, has emerged as a potentially curative treatment option for patients with early stage, medically inoperable non-small cell lung cancer (NSCLC). In a landmark study RTOG (Radiation Therapy Oncology Group) 0236, 59 medically inoperable patients with primary tumors ≤5 cm received 54 Gy (Gray) in 3 fractions [8]. The estimated 3-year primary tumor and local regional control rates were 97.6% (95% CI [Confidence Interval] 84.3-99.7%) and 87.2% (95% CI 71-94.7%). The 3-year distant failure rate was 22.1% (95% CI 12.3-37.8%), 3-year disease free survival (DFS) was 48.3% (95% CI 34.4-60.8%), and 3-year OS was 55.8% (95% CI 41.6-67.9%). Other early prospective trials confirmed high rates of in-field control exceeding 88-98% and 3year survival rates of 38-76% [9, 10]. A recent meta-analysis analyzed 4850 patients in 40 studies treated with SAR. The authors identified 1, 3, and 5 year OS of 83.4%, 56.6% and 41.2%, respectively [11]. In aggregate, robust prospective data has established SAR as the standard-of-care for medically inoperable, early stage NSCLC. Although the majority of prospective trials have enrolled patients with T1-2N0 disease, a body of literature suggests SBRT is also a safe and effective treatment for T3N0 tumors <5 cm in diameter [54], and T3N0 patients were eligible on the landmark RTOG 0236 [8].

Especially important in this patient population is that SAR is well tolerated. In the RTOG 0236 study, grade 3 and 4 toxicity was observed in 13% and 4% of patients, respectively. Only 2 of the 59 patients (3.3%) developed grade 3 pneumonitis. Similarly, secondary analyses demonstrated no clinically significant change in pulmonary function testing following SAR, and poor baseline pulmonary function did not predict for decreased survival [16]. Large published series specifically evaluating the risk of pneumonitis following SAR are listed in Table 1 and consistently demonstrated a low rate of significant pneumonitis and no deaths [12-15].

Table 1. Radiation Pneumonitis Following SAR

Study	# patients	Grade 3+	Grade 4+	Grade 5
Chang JY [12]	130	2.3%	0%	0
Matsuo Y [13]	74	1.4%	0%	0
Baker R [14]	240	1.3%	0%	0
Barriger RB [15]	251	2%	0.4%	0

Despite impressive local control, distant failure remains problematic and 30% of patients will die from distant metastases within 3 years. A systematic review of patterns-of-failure following SAR

identified regional failure of <5-11.3% and distant failure of 11.1-29.2% in studies with median follow-up of at least 30 months [17]. T2-3 lesions, increasing diameter, higher grade, non-squamous histology, and increased SUV (standardized uptake value) were all associated with increased risk of distant metastases [8, 18-20]. Systemic therapy to decrease regional and distant failure in selected higher-risk patients would be logical; however the factors that lead to medical inoperability often preclude the use of conventional cytotoxic chemotherapy following SAR. This necessitates the integration of novel agents that have mild toxicity profiles. In this proposal, we will evaluate the use of an exciting novel and tolerable immune checkpoint inhibitor to improve efficacy by treating subclinical distant micrometastases.

2.3 Cancer Immunotherapy

The allure of cancer immunotherapy as a magic bullet against cancer has intrigued researchers for over a century. The rationale underlying anti-cancer immunotherapy stems from the concept of immune surveillance first attributed to Ehrlich and colleagues over a century ago [21]. It was founded in the idea that tissue rejection is actually a manifestation of an immune surveillance mechanism that guards against spontaneous arising tumors. If such a mechanism does exist then it stands to reason that it can be re-invigorated and harnessed to battle malignancy in cancer patients. This idea, in its simplest form, is particularly attractive given that the immune system should be able to identify and specifically eradicate malignant cells based on the expression of abnormal antigens not expressed or present in normal tissues [22]. On a cellular level antigen presenting cells (APCs), such as dendritic cells, phagocytize fragments of dying cancer cells and present them on their cell surface. CD8+ T cells can recognize these abnormal antigens and become activated to kill cells expressing that antigen. CD4+ T cells can either help this process by expressing ligands and cytokines which help activate and sustain the APCs or they can become immunosuppressive regulatory T cells (Tregs) which express FOXP3 (forkhead box P3) and inhibit CD8+ T cells. In reality this is a gross oversimplification and the true complexity of the interactions between the host immune system and cancer are not fully understood but many cell types and factors are involved.

2.4 Immune Checkpoint Inhibitors

Immune checkpoint signaling pathways are important for switching CD8+ T cells on and off as necessary to defend against foreign stimuli; for example, the immune checkpoint inhibitory pathways naturally turn off CD8+ T cells by receptor-ligand interactions with other immune partners such as APC and Treg cells to keep them from being in a state of chronic activation [23]. Tumors exploit these inhibitory pathways to avoid cell death by producing the cognate ligands that then bind to the inhibitory receptors on CD8+ T cells leading to CD8+ T cells inactivation and inability to attack. An inhibitory checkpoint pathway of great interest is PD-1 (Program death-1) (Figure 1). The PD-1 protein is a co-T-cell regulatory receptor expressed on

CD8+ T cells and Tregs that mediates immunosuppression by binding to its two ligands (Program death-Ligand 1) PD-L1 and PD-L2 (Program death-Ligand 2/not shown) that are expressed by partnering immune cells (APC) and tumor cells [23]. As a result, a dampened immune response occurs and tumor cells survive. In preclinical tumor models the inhibition of this receptor-ligand interaction leads to an enhanced T-cell response and increased tumor killing.

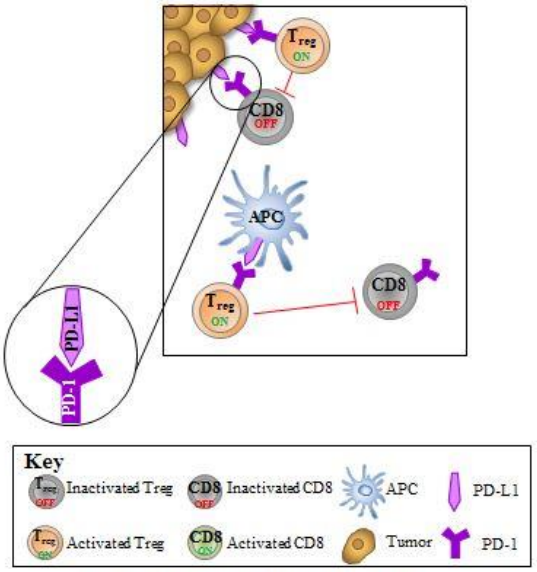

Figure 1: PD-1 Inhibitory Pathway

Several monoclonal antibodies have been developed to block the activation of the PD-1 inhibitory pathway and have been tested in heavily pretreated patients with advanced disease. Table 2 summarizes the agent's characteristics, early clinical activity and safety in the lung cancer patients participating in phase I trials [24-28].

Table 2. Preliminary Safety and Efficacy of PD-1/PD-L1 Inhibitors in Lung Cancer

Agent	Target	Structure	ADCC	ORR	Grade 3-5 AE
Nivolumab	PD -1	IgG4	Reduced	17% (22/129)	6%<br>2% pneumonitis
Pembrolizumab	PD-1	IgG4	Reduced	20% (39/194)	10%<br>2% pneumonitis
MPDL3280A	PD-L1	IgG1	Absent	23% (12/53)	12%<br>No pneumonitis
MEDI4736	PD-L1	IgG1	Absent	16% (9/58)	3% (N=155)<br>No pneumonitis

Abbreviations: ADCC–antibody dependent cellular cytotoxicity; ORR- objective response rate; AE – adverse event; PD-1 – programmed death -1; PD-L1 – program death-ligand 1

A remarkable finding is the consistent antitumor activity with objective response rates (ORR) ranging from 17% to 23%. Moreover all trials reported that majority of responses occurred by the first tumor assessment, were durable and can last beyond treatment discontinuation. Importantly, efficacy was seen across all histological subtypes. The agents were safe and well tolerated with low rates of grade 3 or higher adverse events (AEs). The most mature trial with nivolumab (a PD-1 inhibitor) reported a 1 year OS rate of 42% with a median OS of 9.9 months [25]. The median progression free survival (PFS) was 2.3 months. A host of clinical trials in the first and second line advanced disease settings as monotherapy and in combination with chemotherapy and targeted therapies are being conducted. Therapies targeting this pathway have now been approved for metastatic melanoma and metastatic non-small cell lung cancer.

Preliminary data of pembrolizumab (a PD-1 inhibitor) in 42 untreated patients with advanced lung cancer demonstrated an ORR of 26% and an interim PFS of 26 weeks [29]. Likewise a smaller study with nivolumab showed an ORR of 30% (6/20 patients) with a 36 week PFS and a 1 year OS rate of 75% in untreated metastatic patients [30]. Nivolumab in combination with erlotinib in patients with EGFR (epidermal growth factor receptor) mutated tumors previously treated with erlotinib reported a response rate of 19% (4/21 patients) and in combination with platinum based doublets showed responses ranging between 33–47% in 56 patients across all three doublets evaluated with median PFS ranging from 21-31 weeks [31, 32]. In summary, this data has generated overwhelming enthusiasm for the evaluation of immune checkpoint inhibitors across all stages of lung cancer. Clinical trials in the adjuvant setting are expected shortly.

2.5 MPDL3280A

MPDL3280A is a human immunoglobulin (Ig) G1 monoclonal antibody consisting of two heavy chains (448 amino acids) and two light chains (214 amino acids) and is produced in Chinese hamster ovary cells. MPDL3280A was engineered to eliminate Fc-effector function via a single amino acid substitution (asparagine to alanine) at position 298 on the heavy chain, which results in a non-glycosylated antibody that has minimal binding to Fc receptors and prevents Fc-effector function at expected concentrations in humans. MPDL3280A targets human programmed death-ligand 1 (PD-L1) and inhibits its interaction with its receptor, programmed death-1 (PD-1).

Inhibitors to PD-L1 (MPDL3280A and MEDI4736, Table 2), are theoretically more advantageous than PD-1 inhibitors because 1) PD-L1 can dually bind to another PD-1 family receptor B7.1 found on T cells. By blocking both receptor interactions allows additional restoration of T-cell activity and enhanced T-cell priming. 2) Leaving the PD-1/PD-L2 interaction intact may maintain immune homeostasis and prevent autoimmunity (such as the autoimmunity that causes pneumonitis). 3) The lack of ADCC (antibody-dependent cellular cytotoxicity) will avoid depletion of activated T cells and tumor infiltrating cells.

In a phase I trial conducted in 171 patients with advanced solid tumors, MPDL3280A was administered intravenously every 3 weeks in doses ranging from 0.03 to 20 mg (milligram)/kg (kilogram) [33]. No dose limiting toxicity (DLT) was seen. In the dose expansion phase, the 10, 15 and 20 mg/kg doses were selected for further evaluation. Fifty-three of the 141 patients enrolled had NSCLC [34]. An overall ORR was seen in 22% (9/41patients). A response was seen in 19% (6/31 patients) with non-squamous cell histology and 33% (3/9 patients) with

squamous cell histology. Responses were rapid and durable. The 24 week PFS was 46%. Adverse event data was available on all patients treated on this trial. Toxicities were primarily grade 1-2 and did not require an intervention. Grade 3-4 AEs were infrequent and included fatigue 4% and dyspnea 3%. Immune related grade 3-4 events were seen in 4 patients. No grade 3-4 pneumonitis was observed and no toxic deaths were reported. The most common AEs were fatigue (43%), cough (26%), diarrhea (26%), nausea (25%), decreased appetite (25%), headache (25%), constipation (25%) dyspnea (23%), pyrexia (22%), arthralgias (19%), rash (18%) and insomnia (18%). Overall MPDL3280A was active and well tolerated in lung cancer patients. A dose of 15 mg/kg (equivalent to 1200 mg flat dosing) was the recommended phase II dose.

Based on the tolerability, mild toxicity profile and efficacy of immune checkpoint inhibitors in heavily pretreated lung cancer patients, we hypothesize that they could present a feasible treatment option for patients with early stage inoperable disease. To date, no studies have been performed in early stage lung cancer, or in combination with SAR. The proposed study will fill this gap in our knowledge. MPDL3280A was chosen as the study agent because it is a PD-L1 inhibitor and brings the advantages discussed above over PD-1 inhibitor, has demonstrated efficacy and is well tolerated, with no reports of significant pneumonitis which would theoretically be the most likely overlapping toxicity between these therapies.

2.6 Immune Checkpoint Inhibitors Plus Stereotactic Ablative Radiotherapy

Radiotherapy is in many respects an ideal candidate for combining with immune therapies. In addition to debulking tumor and releasing tumor antigens, radiotherapy has potent immunomodulatory effects and, as opposed to chemotherapy, is not systemically immunosuppressive. The immunomodulatory effects of radiotherapy are well established in the literature [35, 36] and include shifting tumor associated macrophage polarization [37], normalization of tumor vasculature [37], improving T-cell homing to tumor sites [37], destruction of immunosuppressive stromal cells in the tumor [37], microenvironment [38], induction of immunogenic cell death [39, 40] amongst many others. Our data, (described in the next section) as well as other preclinical data, demonstrates that radiotherapy can improve the efficacy of immunotherapy including checkpoint blockade [41, 42]. This is substantiated by clinical studies and case reports that have demonstrated this combination is safe and with promising efficacy. A phase I trial combining one, two or three doses of local SAR at 20 Gy per fraction with IL-2 (Interleukin-2) for metastatic melanoma did not demonstrated any dose limiting toxicities. A striking 71.4% systemic response rate, far superior to historical controls with IL-2 alone, was observed [43]. The majority of responders had long term durable responses and the toxicity was no higher than what was expected with IL-2 alone. A phase I/II study of ipilimumab (another immune checkpoint inhibitor) at 3 or 10 mg/kg intravenously plus a single 8 Gy fraction to 1-3 bone lesions was evaluated in 41 patients with metastatic castrate resistant prostate cancer (MCRPC) [44]. There was no DLT. In the entire group treatment related grade 3 or 4 AEs occurred in 39% of patients and 22% were immune related. Six patients (15%) had confirmed declines in their PSA (prostate-specific antigen). One patient had a partial response and 15% of patients had stable disease. A phase III trial of ipilimumab 10 mg/kg plus bone directed radiotherapy versus placebo and radiotherapy in MCRPC has completed accrual. A recent case report in the New England Journal of Medicine illustrates the ability of radiotherapy to induce a systemic response to CTLA-4 (Cytotoxic T-lymphocyte-associated protein 4)

checkpoint blockade in a melanoma patient who was previously not responding to therapy [45]. A number of similar case studies with ipilimumab have since been reported including a case of long-term durable complete response in a patient with metastatic refractory NSCLC.[46]. Activity with radiotherapy has also been seen with other immune stimulatory agents. TLR9 (toll-like receptor 9) agonists are known to induce and activate dendritic cells and B cells. In combination with radiotherapy an objective systemic responses was observed in 20% of patients and disease stability in about another 20% of patients with heavily pretreated systemic [47] or cutaneous lymphoma [48].

2.7 Biomarkers

In this era of precision medicine the ability to select patients most likely to benefit from a treatment has become an integral component of drug development. For immune checkpoint inhibitors the leading candidate is immunohistochemical (IHC) expression of tumor PD-L1. However marked controversy revolves around its ultimate relevance as a predictive biomarker because durable responses have been seen in patients with and without PD-L1 expression [26, 27, 31]. Complicating the interpretation of this data is the different IHC antibodies used and the variable positive cutoff points. Nonetheless numerically higher response rates have been observed in patients with PD-L1 positive tumors. In the phase I MPDL3280A trial the objective responses were 80% (4/5 NSCLC patients) with PD-L1 tumor positivity and 14% (4/28 NSCLC patients) without PD-L1 tumor positivity [27]. The definitive role of PD-L1 expression is being evaluated in randomized phase III trials.

As the science emerges other promising biomarkers are being investigated such as PD-L1 expression by immune cells in the tumor microenvironment, a tumor immune checkpoint IHC signature (Immunochip) that incorporates other pathway proteins, mutational load, and blood based cytokine and immune cellular profiles. High mutational load is an interesting candidate marker of response. Genomic data has shown that smoking induced lung cancer is associated with one of the highest mutational rates [49]. In the MPDL3280A trial a higher response rate was observed in current or former smokers 26% (11/42 patients) compared with 10% (1/10 patients) in never smokers [27]. This is pertinent for our study because we expect the majority of our patients to have a smoking history. Thus, a comprehensive analysis of tumor and blood will provide valuable insight into potential biomarkers and into the mechanism of action of our combined therapy. We recognize that our tumor analysis will be limited in this patient population who may have only had a fine needle aspirate for diagnosis therefore we will focus primarily on analyzing serial blood samples. This is timely because “liquid biopsies” for predictive marker analysis are emerging as the future for companion diagnostics of new therapies. To date, there remains to be defined a predictive peripheral immune marker for checkpoint blockade immunotherapy. A recent immunophenotyping FACS panel from the NCI has demonstrated some promise in this regard (unpublished communication from Dr. Renee Donahue) and will be used in this study.

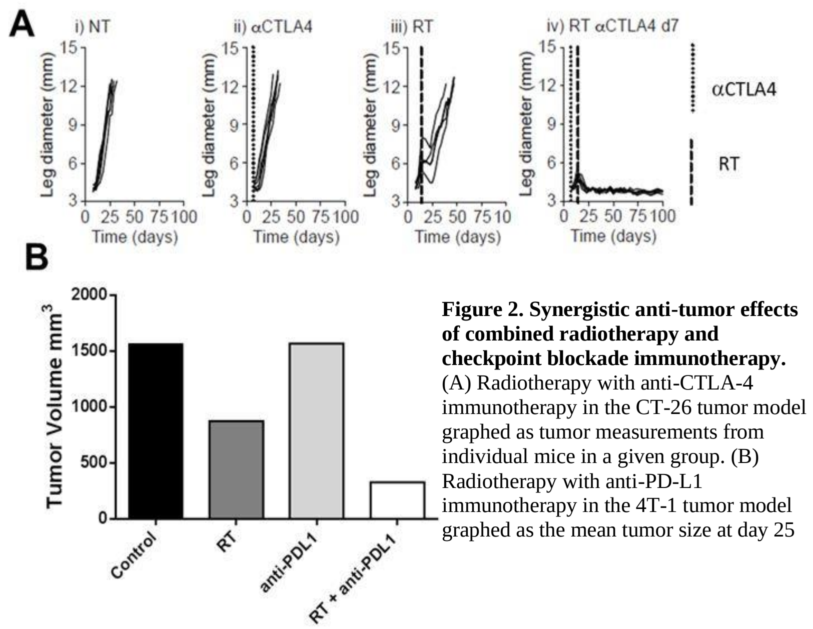

Figure 2. Synergistic anti-tumor effects of combined radiotherapy and checkpoint blockade immunotherapy. (A) Radiotherapy with anti-CTLA-4 immunotherapy in the CT-26 tumor model graphed as tumor measurements from individual mice in a given group. (B) Radiotherapy with anti-PD-L1 immunotherapy in the 4T-1 tumor model graphed as the mean tumor size at day 25

2.8 Preliminary Data

Preliminary data (Figure 2) examining radiotherapy with checkpoint blockade demonstrate a synergistic effect of combining these therapies. In our first experiments (Figure 2A), radiotherapy was delivered as a single fraction of 20 Gy to the tumor on day 14 post tumor implantation and anti-CTLA-4 (clone 9D9) was administered as a single i.p. (intraperitoneal) injection of 250ug on day 7. As expected, and corroborated by other investigators, minimal antitumor activity is typically observed with single agent immune checkpoint inhibitors in mouse models. The combination of radiotherapy with anti-CTLA-4 checkpoint blockade resulted in 100% of mice being cured. Additional studies examining the immunologic mechanism underlying these observations are underway. Duplicate experiments with anti-PD-L1 checkpoint blockade (clone 10F.9G2, 200ug i.p. in the aggressive 4T-1 tumor model likewise reveal minimal activity of the immunotherapy alone but robust effects in combination with radiotherapy (Figure 2B). Our preliminary data also revealed improved survival with combination therapy and indicate that the therapy is most effective when the checkpoint blockade is administered prior to radiotherapy (data not shown).

To gain insight into the T cell response in patients treated with SAR we recently began collecting pre- and serial post-therapy blood samples in patients with stage I lung cancer receiving SAR with IRB (Institutional Review Board) approval. Three patients have had pretreatment and one week post SAR blood collection for analysis. Figure 3 demonstrates our FACS (Fluorescence-Activated Cell Sorting) staining schema for CD8+ T cell function using granzyme b as a marker of T cell functionality. Purified human peripheral blood mononuclear cells were gated for live CD3+ cells. The left panels demonstrate the gating schema for CD8+ and CD4+ cells. The right panels demonstrate the gating schema for granzyme b on the CD8+CD4- gated cells. There is no granzyme b staining in the isotype controls (negative control, upper panels) however there is high level of activated and functional CD8+ T cells in the peripheral blood after SAR (bottom panels). These results support our hypothesis that SAR induces CD8+ T cell activation and function but that the addition of a checkpoint blockade will support the anti-tumor activity of these cells in the microenvironment. Changes in the profile of systemic cytokines are also being examined in the plasma of these patients and the results of these studies are pending. This supports our mechanistic hypothesis on how SAR and MPDL3280A might interact. As illustrated in Figure 4 the top panel shows radiotherapy can debulk the tumor, activate anti-tumor CD8+ T cells and increase their migration into the tumor micro-environment but can also induce the upregulation of PD-1 on T cells and PD-L1 in the tumor micro-environment. The interaction of PD-1/PD-L1 and the activity of Tregs prevent an effective anti-tumor immune response. The bottom panel shows that combining radiotherapy with PD-L1 blockade can increase the activation and number of anti-tumor CD8+ T cells in the tumor microenvironment, prevent the activation of and induction of Tregs, and block the inhibition of CD8+ T cells leading to an effective anti-tumor immune response.

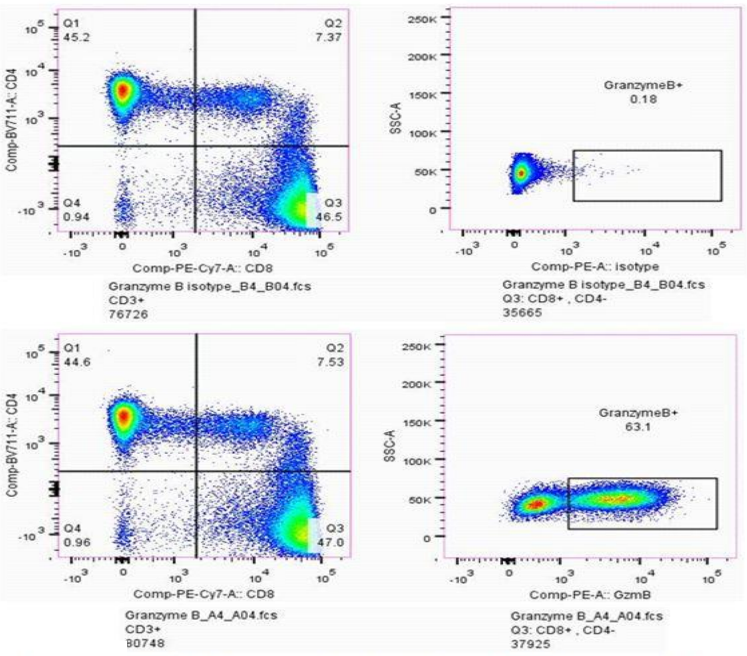

Figure 3. <u>**Gating strategy for granzyme b expression in human peripheral blood lymphocytes**</u>. Left panels demonstrate gating for CD4/CD8 on the CD3+ population. The right panels demonstrate gating for granzyme b on the CD8+ CD4- subpopulation. The bottom panels demonstrated a high level of granzymeb+ CD8+ T cells in this patient post lung SAR.

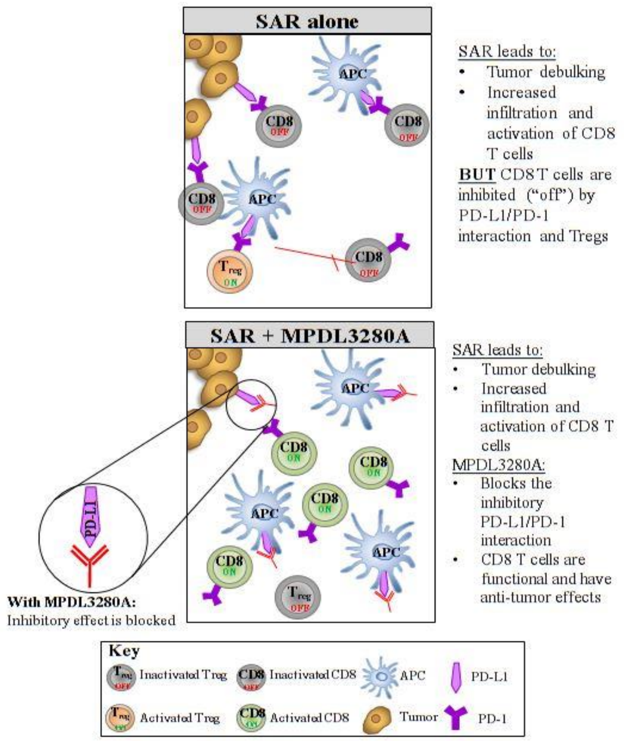

Figure 4: Proposed mechanism for synergistic activity of radiotherapy and PD-L1 checkpoint blockade

2.9 Rationale

We hypothesize that the interplay between radiation and the immune system to promote tumor cell killing will be safely enhanced by the delivery of modern SAR in combination with a novel immune checkpoint inhibitor MPDL3280A resulting in better local tumor control, the eradication of systemic micrometastasis and ultimately an increase in the cure rate for patients with inoperable early stage NSCLC.

We will conduct a phase I clinical trial of MPDL3280A plus SAR in patients with inoperable early stage NSCLC. Based on our preclinical data we postulate the optimal approach involves priming the immune system with MPDL3280A followed by concurrent administration of MPDL3280A with SAR and then 3 cycles of adjuvant MPDL3280A. In a complimentary manner we will conduct immune and molecular profiling on patient samples to elucidate the biological effects of this treatment and to identify candidate biomarkers of response that could be further evaluated and used to select patients for this therapy.

3.0 STUDY DESIGN

This is a proof of concept phase I study that will use a standard 3 + 3 dose escalation design followed by a 15 patient expansion as illustrated in the schema. Three doses will be evaluated: 3mg/kg, 10 mg/kg and 1200 mg (equivalent to 15 mg/kg). These 3 dose levels were selected based on the phase I data described above. The 10 and 15 mg/kg doses were evaluated in the

lung cancer cohort and were shown to be active and tolerable. The 15 mg/kg dose was recommended for phase III trials and eventually switched to a flat 1200 mg for prospective studies. One dose level below these, 3 mg/kg, was chosen as our initial starting dose and has shown antitumor activity. There is no -1 dose level. If dose level 1 (3 mg/kg) is not tolerated the study will be halted. MPDL3280A will be administered on day 1 every 3 weeks for a total of 6 cycles. All patients will receive two doses of MPDL3280A to prime the immune system followed by the concurrent administration of MPDL3280A plus SAR (12.5 Gy/fraction for 4 fractions for peripherally located tumors and 10 Gy/fraction for 5 fractions for centrally located tumors) delivered on non-consecutive days within a 14 day period during cycle 3. Patients will then receive 3 additional cycles of MPDL3280A. The 6-cycle regimen was arbitrarily chosen to align with current adjuvant regimens where 3 or 4 cycles of chemotherapy are standard after definitive treatment. A longer duration of therapy was considered but it was decided that a prolonged treatment time would hinder accrual and led to poorer treatment compliance in this patient population. The expansion phase will be conducted using the MTD defined as the highest dose at which no more than one of six patients develops a DLT or Dose Level 3 if the MTD is not reached.

A DLT is defined as ≥ grade 3 immune related adverse event OR other ≥ grade 3 treatment related adverse events that do not resolve to ≤ grade 2 within 14 days of onset during the 9 week DLT period OR Grade 2 diarrhea, AST/ALT > 3 x ULN with bilirubin > 2 X ULN, or pneumonitis that requires holding treatment > 14 days. Table 3 describes the standard dosing rules that will be followed.

**Table 3. Dose Escalation Rules**	
Number of Patients with DLT (dose limiting toxicity) at a Given Dose Level	Escalation Decision Rule
0 out of 3	Enter 3 patients at the next dose level
≥2 out of 3	Dose escalation will be stopped. This dose level will be declared the maximum administered dose (highest dose administered). Three (3) additional patients will be entered at the previous dose level if only 3 patients were treated previously at that dose.
1 out of 3	Enter at least 3 more patients at this dose level. If 0 of these additional 3 patients experience DLT, proceed to the next dose level. If 1 or more of these additional patients suffer DLT, then dose escalation is stopped, and this dose is declared the maximally administered dose. Three (3) additional patients will be entered at the next lowest dose level if only 3 patients were treated previously at that dose.
<1 out of 6 at the highest dose level below the maximally administered dose	This is generally the recommended phase 2 dose.

Patients will be monitored weekly during the 9-week DLT period and every 3 weeks for the remaining three cycles or more frequently if needed. All patients on active treatment will be discussed at weekly teleconferences that are held between the UC (University of California) Davis team and Dr. Mitchell at David Grant USAF (United States Air Force) Medical Center.

Per Cancer Center guidelines, a trial cannot proceed to the next dose level until a DLT meeting is conducted to comprehensively review all toxicity data and approve the dose escalation. All patients will be monitored for a minimum of 12 months following SAR to evaluate for long-term radiation effects. Preliminary efficacy as determined by ORR and DFS will be assessed every 2 cycles then every 3 months for the first two years and every 6 months for the years 3-5.

4.0 STUDY POPULATION

Patients will be recruited from:
- UC Davis Comprehensive Cancer Center (UCDCCC)
- VA Northern California Health Care System (VANCHCS), which includes VA Mather in Sacramento, CA; VA Martinez in Martinez, CA; and David Grant USAF Medical Center at Travis Air Force Base
- Cedars-Sinai Medical Center

UCDCCC: Patients will be identified at the UC Davis multidisciplinary lung cancer tumor board where new cases are presented and in pulmonary, thoracic surgery and radiation oncology across the UC Davis Health System. Broad recruitment efforts at UC Davis include 1) discussion of the trial at the bimonthly clinical investigators meeting upon trial activation, 2) weekly email broadcasts of phase I trial slot availability; and 3) monthly cancer clinical trials updates to all oncologists, radiation oncologists and surgeons in the northern and central California region. Mercy: Patients will be identified by the radiation oncologists at Mercy Cancer Center or Mercy San Juan Medical Center and referred to UCDCCC for consultation about the trial. Trial flyers and monthly email reminders are sent to these physicians.

Military patients and families: Identification of veterans for trial eligibility will be conducted by UC Davis faculty that staff VA Mather and VA Martinez and by radiation oncologists at David Grant USAF Medical Center. David Grant USAF Medical Center physicians may also recruit other military service members or their families. Monthly email reminders are sent to these physicians.

Cedars-Sinai Medical Center: Patients will be identified through their multidisciplinary lung cancer tumor board and email reminders. PIs will participate in the monthly trial updates meeting.

Table 4. Stage I NSCLC patients treated with SAR at UC Davis

	2010	2011	2012	2013
Number of Patients	16	16	23	28
Number of VA Patients	5	2	3	4

Abbreviations: NSCLC-Non-small cell lung cancer; SAR-Stereotactic Ablative Radiotherapy

The number of stage I lung cancer patients and VA patients treated at UC Davis with SARs over the past 4 years is shown in Table 4. From 2010-2013 17% of patients treated at UC Davis were veterans. David Grant USAF Medical Center treats 15-20 stage I patients per year, and Cedars-

Sinai Medical Center treats 20 patients per year. Based on a minimum of 63 patients/year and a conservative estimate that 35% of these patients will meet the eligibility criteria and participate in the trial, we will complete enrollment in the projected 27 month accrual period. With the national implementation of low dose CT screening in January 2015 we expect to see an increase the number of early stage lung cancer patients that would be potential candidates for our trial.

4.1 Inclusion Criteria

1. Adults ≥18 years of age with histologically proven T1-3N0M0 NSCLC ≤5 cm diameter (patients with tumor size up to 7 cm are allowed if radiation dose/volume histogram constraints for normal tissues can be met). T3 patients with chest wall invasion or 2 nodules within the same lobe are eligible.
2. One or more high-risk features identified:
   a. Tumor diameter ≥ 1 cm (phase I component) or ≥ 2 cm (phase II component)
   b. Tumor SUV max ≥ 6.2
   c. Moderately, poorly differentiated or undifferentiated histology
3. Evaluable disease per RECIST 1.1
4. Patients must be medically or surgically inoperable as determined by a physician OR unwilling to undergo surgical resection. Inoperability is determined by a board-certified thoracic surgeon. This is not a member of the study team, but as standard-of-care these patients are either seen by a board certified thoracic surgeon and referred for SBRT or discussed at a multidisciplinary thoracic tumor board with a thoracic surgeon present. Medical inoperability is defined as the presence of underlying physiological medical problems that would prohibit a PCR (pathological complete resection) due to a low probability of tolerating general anesthesia, the operation, the post-operative recovery period, or the removal of adjacent functioning lung. Standard justification for deeming a patient medically inoperable based on pulmonary function for surgical resection of NSCLC will include any of the following: Baseline FEV1 < 40% predicted, post-operative FEV1 < 30% predicted; severely reduced diffusion capacity; baseline hypoxemia and/or hypercapnia; exercise oxygen consumption < 50% predicted; severe pulmonary hypertension; diabetes mellitus with severe end organ damage; severe cerebral, cardiac, or peripheral vascular disease; severe chronic heart disease; or significant functional limitations that preclude the ability to take part in post-surgical pulmonary rehabilitation.
5. All patients must have an FEV1 ≥ 700cc and a DLCO ≥ 5.5 m/min/mmHg.
6. ECOG (Eastern Cooperative Oncology Group) performance status score of 0 – 2 (Appendix 1)
7. Life expectancy ≥ 12 months
8. Adequate hematologic and end organ function, defined by the following laboratory results obtained within 14 days of the first study treatment:
   - ANC > 1500 cells/ul; WBC count > 2500/uL; Lymphocyte count >500/uL; Platelet count > 100,000/uL; Hemoglobin > 9 g/dL
   - Liver function tests meeting one of the following criteria:
     a. AST and ALT < 2.5 x ULN with alkaline phosphatase ≤ 2.5 x ULN OR
     b. AST and ALT ≤ 1.5 x ULN, with alkaline phosphatase > 2.5 x ULN
     c. Serum bilirubin ≤ 1.0 x ULN

- INR and aPTT < 1.5 x ULN (for patients on anticoagulation they must be receiving a stable dose for at least 1 week prior to enrollment)
- Creatinine clearance > 30 mL/min by Cockcroft-Gault formula.

9. No history of severe hypersensitivity reactions to other mAbs.
10. No other active malignancy.
11. Patients with a history of autoimmune-related hypothyroidism on a stable dose of thyroid replacement hormone are eligible
12. Patients with controlled Type 1 diabetes mellitus on a stable insulin regimen are eligible.
13. Archival tumor sample available. Tissue from an FNA (fine-needle aspiration) is allowed but tumor tissue from a core needle biopsy is preferred
14. For female patients of childbearing potential and male patients with partners of childbearing potential agreement (by patient and/or partner) to use highly effective form(s) of contraception (i.e., one that results in a low failure rate [<1% per year] when used consistently and correctly) and to continue its use for 6 months after the last dose of MPDL3280A.
15. Signed informed consent.
16. Ability to comply with the protocol

4.2 Exclusion Criteria

1. Uncontrolled concomitant disease
2. Significant cardiovascular disease (NYHA Class II or greater); myocardial infarction within 3 month prior to randomization, unstable arrhythmias, unstable angina or a patient with a known LVEF (Left Ventricular Ejection Fraction) < 40%
3. Severe infection within 4 weeks prior to enrollment
4. Active tuberculosis
5. Oral or IV antibiotics within 2 weeks or 5 half-lives prior to enrollment
6. History of autoimmune disease.
7. Positive for Human Immunodeficiency Virus (HIV), Hepatitis B (Hepatitis B Surface Antigen [HBsAg] reactive), or Hepatitis C virus (Hepatitis C Virus Ribonucleic Acid [HCV RNA] (qualitative) is detected)
8. History of idiopathic pulmonary fibrosis, drug-induced pneumonitis, organizing pneumonia (i.e. bronchiolitis obliterans, cryptogenic organizing pneumonia, or evidence of active pneumonitis on the screening chest CT).
9. Treatment with systemic immunostimulatory agents within 4 weeks or five half-lives of the drug, whichever is shorter, prior to enrollment.
10. Treatment with systemic corticosteroids or other systemic immunosuppressive medications within past 4 weeks or 5 half-lives whichever is shorter.
11. Pregnant and/or lactating women.

5.0 TREATMENT PLAN

5.1 MPDL3280A

5.1.1 Dosage, Administration, and Compliance

MPDL3280Awill be administered at doses of 3 mg/kg, 10 mg/kg and 1200 mg intravenously on Day 1 every 3 weeks for 6 cycles. On cycle 3 MPDLA3280A will be administered 24-48 hours prior to the initiation of SAR. Study drug will be administered at the UC Davis phase I infusion center or Cedars-Sinai Medical Center. SAR will be administered at UC Davis, Mercy (for patients referred from Mercy Cancer Center or Mercy San Juan Medical Center), or Cedars-Sinai Medical Center.

Administration of MPDL3280A will be performed in a setting with emergency medical facilities and staff who are trained to monitor for and respond to medical emergencies. MPDL3280A will be delivered in infusion bags with IV infusion lines with product contacting surfaces of polyvinylchloride (PVC) and polyolefin and 0.2 µm in-line filters (filter membrane of polyethersulfone [PES]). No incompatibilities have been observed between MPDL3280A and PVC or polyolefin infusion materials (bags and infusion lines).

The initial dose of MPDL3280A will be delivered over 60 (±15) minutes. If the first infusion is tolerated without infusion-associated adverse events, the second infusion may be delivered over 30 (±10) minutes. If the 30-minute infusion is well tolerated, all subsequent infusions may be delivered over 30 (±10) minutes. For the first infusion, the patient's vital signs (heart rate, respiratory rate, blood pressures, and temperature) should be determined within 60 minutes before, during (every 15 [±5] minutes), and 30 (±10) minutes after the infusion. For subsequent infusions, vital signs will be collected within 60 minutes before infusion. Patients will be informed about the possibility of delayed post-infusion symptoms and instructed to contact their study physician if they develop acute symptoms.

No premedication will be allowed for the first dose of MPDL3280A. Premedication may be administered for Cycles ≥2 at the discretion of the treating physician. The management of infusion-related reactions will be according to severity as follows:

- In the event that a patient experiences a mild (NCI CTCAE Grade 1) infusion-related event, the infusion rate should be reduced to half the rate being given at the time of event onset. Once the event has resolved, the investigator should continue to deliver the infusion at the reduced rate for 30 minutes. If tolerated, the infusion rate may then be increased to the original rate.
- In the event that a patient experiences a moderate infusion-related event (NCI CTCAE Grade 2) or flushing, fever, or throat pain, the patient should have his or her infusion immediately interrupted and should receive aggressive symptomatic treatment. The infusion should be restarted only after the symptoms have adequately resolved to the baseline grade. The infusion rate at restart should be half of the rate that was in progress at the time of the onset of the infusion-related event.

- For severe or life-threatening infusion-related events (NCI CTCAE Grade 3 or 4), the infusion should be stopped immediately, and aggressive resuscitation and supportive measures should be initiated. Patients experiencing severe or life-threatening infusion-related events will not receive further infusion and will be further managed as clinically indicated until the event resolves.

For anaphylaxis precautions, see Appendix 5.

Guidelines for dosage medication, treatment interruption, or discontinuation and the management of specific adverse events are provided in Section 6.0.

MPDL3280A will be supplied by Genentech at no cost to study patients.

5.1.2 Formulation, Packaging, and Handling

The MPDL3280A drug product is provide in a single-use, 20-cc USP/Ph. Eur. Type 1 glass vial as a colorless to slightly yellow, sterile, preservative-free clear liquid solution intended for IV administration. The vial is designed to deliver 20.0 mL (1200 mg) of MPDL3280A solution but may contain more than the stated volume to enable delivery of the entire 20.0 mL volume. The MPDL3280A drug product is formulated as 60 mg/mL MPDL3280A in 20 mM histidine acetate, 120 mM sucrose, 0.04% polysorbate 20, pH 5.8.
MPDL3280A must be refrigerated at 2°C-8°C (36°F-46°F) upon receipt until use. MPDL3280A vials should not be used beyond the expiration date provided by the manufacturer. No preservative is used in the MPDL3280A drug product; therefore, each vial is intended for single use only. Discard any unused portion of drug left in a vial. Vial contents should not be frozen or shaken and should be protected from direct sunlight.

For further details, see the Investigator's Brochure.

5.1.3 Disposal and Destruction

Drug supply will either be disposed of at the study site according to the study site's institutional standard operating procedure or returned to Genentech with the appropriate documentation. The site's method of drug supply destruction must be agreed upon by Genentech.

Accurate records of all investigational product received at, dispensed from, returned to, and disposed of by the study site should be recorded on the Drug Log.

5.2 SAR

5.2.1 Dose Specifications and Treatment Delivery

SAR will be performed to 50 Gy over 4 fractions of 12/5 Gy each for peripherally located tumors and 50 Gy over 5 fractions of 10 Gy each for centrally located tumors (within 2 cm of the proximal bronchial tree or touching mediastinal pleura). There should be a minimum of 40 hours and a maximum of 96 hours between treatments. Corticosteroid premedication is <u>not</u> permitted. Analgesic premedication to avoid general discomfort during long treatment durations is

recommended when appropriate. The dose per fraction is to be prescribed to the prescription line at the edge of the planning treatment volume (PTV).

5.2.2 Technical Factors

Only photon (x-ray) beams with photon energies 4-15 MV will be allowed. Cobalt-60 and charged particle beams (including electrons, protons, and heavier ions) are not allowed.

5.2.3 Localization, Simulation, and Immobilization

Patients will be positioned in a stable position capable of allowing accurate reproducibility of the target position from treatment to treatment. A variety of immobilization systems may be used, including stereotactic frames that surround the patient on three sides and large rigid pillows. Patient immobilization must be reliable enough to ensure that the gross tumor volume (GTV) does not deviate beyond the confines of the PTV as with any significant probability (i.e., < 5%). A motion management strategy is mandatory if tumor motion secondary to respiratory excursion as assessed by fluoroscopy or 4DCT exceeds 1cm. Acceptable maneuvers include reliable abdominal compression with fluoroscopic verification, accelerator beam gating with the respiratory cycle, tumor tracking, and active breath-holding techniques. Internal organ inhibition maneuvers must be reliable enough to insure that the GTV does not deviate beyond the confines of the PTV with any significant probability (i.e., < 5%). Patients will undergo a tomographic imaging study (Conebeam computed tomography or Megavoltage computed tomography) immediately prior to treatment to ensure proper alignment of the geometric center (i.e., isocenter) of the simulated fields.

5.2.4 Target Volumes

Image Acquisition
Computed tomography will be used for targeting and treatment planning. IV contrast is encouraged when its use will enhance target delineation but is not required. Axial acquisitions with gantry 0 degrees will be required with spacing ≤ 3.0 mm between scans in the region of the tumor. Incorporation of a four-dimensional CT (4DCT) dataset at simulation is strongly encouraged, though is not required.

Target Volume
Using either approach a 4DCT or non-4DCT simulation, the target lesion will be outlined and designated the gross tumor volume (GTV). The target will generally be drawn using CT pulmonary windows; however, soft tissue windows with contrast may be used to avoid inclusion of adjacent vessels, atelectasis, or mediastinal or chest wall structures within the GTV. No additional clinical target volume (CTV) margin for microscopic spread will be added. If helical scanning without 4DCT simulation is used, an additional 0.5 cm in the axial plane and 1.0-1.5 cm in the longitudinal plane (craniocaudal), depending on the degree of motion as assessed by fluoroscopy, will be added to the GTV to constitute the PTV. If 4DCT simulation is obtained, an internal target volume (ITV) will be created from either a maximum intensity projection or a composite of the maximum inspiratory/maximum expiratory phases or of all available phases. An additional uniform 0.5 cm PTV margin will be added.

5.2.5 Treatment Planning

Technique and Dose Calculations
Conformal treatment approaches including 3D conformal radiotherapy using static, preferably non-coplanar fields; intensity modulated radiotherapy (IMRT), Volume Modulated Arc therapy (VMAT), Dynamic Conformal Arc Radiation Therapy (DCART), and helical tomotherapy are acceptable. Prescription lines covering the PTV may range from 60-95%. When IV and/or oral contrast are used, contrast densities should be overridden in the planning process. Tissue density heterogeneity correction is required for lung tumors. Superposition/convolution dose algorithms are preferred.

Target Coverage and Conformality
Successful treatment planning will require accomplishment of all of the following criteria:

1. Normalization: The treatment plan should be normalized such that 100% corresponds to the center of mass of the PTV.
2. Prescription Isodose Surface Coverage: The prescription isodose surface will be chosen such that 95% of the target volume (PTV) is covered by the prescription isodose surface and 99% of the target volume (PTV) receives a minimum of 90% of the prescription dose.
3. Target Dose Heterogeneity: The prescription isodose surface selected must be ≥ 60% of the dose at the center of mass of the PTV (COMPTV) and ≤ 95% of the dose at the center of mass of the PTV (COMPTV). The COMPTV corresponds to the normalization point (100%) of the plan as noted in number 1 above.
4. Respect all critical organ dose-volume limits listed below.

All SAR plans will be centrally reviewed by Dr. Daly prior to delivery of the first fraction.

5.2.6 Critical Structures

The following organs will be contoured for all cases:

Right and Left Lung: Contour based on lung window (auto-segmentation is acceptable)
Bilateral (composite) lung – GTV: Composite lung volume excluding the GTV

Spinal Cord: The spinal cord will be contoured based on the bony limits of the spinal canal. The spinal cord should be contoured starting at least 10 cm above the superior extent of the PTV and continuing on every CT slice to at least 10 below the inferior extent of the PTV.

Proximal Bronchial Tree: Will include the distal 2 cm of the trachea, the carina, the right and left mainstem bronchi, the right and left upper lobe bronchi, the bronchus intermedius, the right middle lobe bronchus, the lingular bronchus and the right and left lower lobe bronchi.

Proximal Trachea: The proximal tracheal will be contoured extending from the laryngeal inlet to 2 cm proximal to the carina.

Esophagus: The external muscular wall of the esophagus will be contoured from the pharyngoesophageal junction to the gastroesophageal junction using mediastinal windows.

Chest Wall: A 1 cm rind will be contoured extending 3 cm superior and inferior to the cranio and caudal aspects of the PTV including the ribs and intercostal muscles.

Ipsilateral Brachial Plexus: The major trunks of the brachial plexus will be contoured using the subclavian and axillary vessels as a surrogate. The neurovascular complex will be contoured starting proximally at the bifurcation of the brachiocephalic trunk into the jugular/subclavian veins (or carotid/subclavian arteries) and following along the route of the subclavian vein to the axillary vein ending after the neurovascular structures cross the second rib.

Heart: Contour the pericardium from the inferior aspect of the aortic arch to the apex of the heart

Skin: A 3 mm rind over the surface of the body will be contoured extending at least 5 cm superior and inferior to the target lesion

Critical Organ Dose-Volume Limits

Table 5 lists maximum dose limits to a point or volume within critical organs for 4 fraction SAR, and Table 6 lists dose volume constraints for 5 fraction SAR. The point maximum doses listed, as well as the volume receiving 20 Gy (V20) for lung-GTV, are absolute limits (except where otherwise noted), and treatment delivery that exceeds these limits will constitute a major protocol violation. Volumetric dose constraints are suggested guidelines, and while every effort should be made to achieve the recommended constraints, exceeding the suggested dose volume metrics (other than for lung-GTV V20) will not constitute a protocol violation.

Table 5. 4 Fraction SAR Constraints

Serial Tissue	Volume	Volume Max (Gy)	Max Point Dose (Gy)
Spinal Cord	<0.35 cc<br><1.2 cc	20.8 Gy (5.2 Gy/fx)	26 Gy (6.5 Gy/fx)
Esophagus	<5 cc	18.8 Gy (4.7 Gy/fx)	30 Gy (7.5 Gy/fx)
Brachial Plexus	<3 cc	23.6 Gy (5.9 Gy/fx)*	27.2 Gy (6.8 Gy/fx)*
Heart/Pericardium	<15 cc	28 Gy (7 Gy/fx)	44 Gy (11 Gy/fx)
Proximal Bronchial Tree	<4 cc	15.6 Gy (3.9 Gy/fx)	34.8 Gy (8.7 Gy/fx)
Skin	<10 cc	33.2 Gy (8.3 Gy/fx)	36 Gy (9 Gy/fx)
**Parallel Tissue**	**Critical Volume (cc)**	**Critical Volume Dose Max (Gy)**	
Chest wall	<30 cc	30 Gy*	
Combined Lung -GTV	1500 cc<br>1000 cc<br>< 10%	11.6 Gy (2.9 Gy/fx)<br>12.4 Gy (3.1 Gy/fx)<br>20 Gy	

Table 6. 5 Fraction SAR Constraints

Serial Tissue	Volume	Volume Max (Gy)	Max Point Dose (Gy)
Spinal Cord	<0.35 cc<br><1.2 cc	23 Gy (4.6 Gy/fx)<br>14.5 Gy (2.9 Gy/fx)	30 Gy (6 Gy/fx)
Esophagus	<5 cc	19.5 Gy (3.9 Gy/fx)	35 Gy (7 Gy/fx)
Brachial Plexus	<3 cc	27 Gy (5.4 Gy/fx)*	30.5 Gy (6.1 Gy/fx)*
Heart/Pericardium	<15 cc	32 Gy (6.4 Gy/fx)	50 Gy (10 Gy/fx)
Proximal Bronchial Tree	<4 cc	16.5 Gy (3.3 Gy/fx)	40 Gy (8 Gy/fx)
Skin	<10 cc	36.5 Gy (7.3 Gy/fx)	39.5 Gy (7.9 Gy/fx)
**Parallel Tissue**	**Critical Volume (cc)**	**Critical Volume Dose Max (Gy)**	
Chest wall	<30 cc	30 Gy*	
Combined Lung -GTV	1500 cc<br>1000 cc<br>< 10%	12.5 Gy (2.5 Gy/fx)<br>13.5 Gy (2.7 Gy/fx)<br>20 Gy	

*Chest wall and brachial plexus constraints are optional but highly encouraged when achievable
Guidelines for Treatment interruption or discontinuation and the management of specific adverse events are provided in Section 6.0.

6.0 GENERAL PLAN TO MANAGE SAFETY CONCERNS

Measures will be taken to ensure the safety of patients participating in this trial, including the use of stringent inclusion and exclusion criteria (see Sections 4.1 and 4.2) and close monitoring (as indicated below and in the study calendar).

Eligibility Criteria

Eligibility criteria were selected to guard the safety of patients in this trial. Results from the nonclinical toxicology studies with MPDL3280A, as well as the nonclinical/clinical data from other PD-L1/PD-1 inhibitors, were taken into account.

Monitoring

Safety will be evaluated in this study through the monitoring of all serious and non-serious AEs, defined and graded according to NCI CTCAE v4.0. Patients will be assessed for safety (including laboratory values) according to the Study Calendar. Patients will be followed for safety for 30 days following the last dose of study treatment or until receipt of another anticancer therapy, whichever comes first.

General safety assessments will include serial interval histories, physical examinations, and specific laboratory studies, including serum chemistries and blood counts (see Study Calendar for the list and timing of study assessments). All serious adverse events (SAEs) and protocol-defined events of special interest will be reported in an expedited fashion. In addition, the investigators will review and evaluate observed AEs on a regular basis.

Patients who have an ongoing study treatment–related AE upon study completion or at discontinuation from the study will be followed until the event has resolved to baseline grade, the event is assessed by the investigator as stable, new anticancer treatment is initiated, the patient is lost to follow-up, the patient withdraws consent, or until it has been determined that study treatment or participation is not the cause of the AE.

6.1 Management of MPDL3280A-Specific Adverse Events

Toxicities associated or possibly associated with MPDL3280A treatment should be managed according to standard medical practice. Additional tests, such as autoimmune serology or biopsies, may be used to determine a possible immunogenic etiology.

Although most immune-related adverse events observed with immunomodulatory agents have been mild and self-limiting, such events should be recognized early and treated promptly to avoid potential major complications. Discontinuation of MPDL3280A may not have an immediate therapeutic effect and in severe cases, immune-related toxicities may require acute management with topical corticosteroids, systemic corticosteroids, mycophenolate, or TNF-α inhibitors.

The primary approach to Grade 1-2 immune-related adverse events is supportive and symptomatic care with continued treatment with MPDL3280A; for higher grade immune-related adverse events, MPDL3280A should be held and oral/parental steroids administered. Recurrent Grade 2 immune-related adverse events may also mandate holding MPDL3280A or the use of steroids. Consideration for benefit/risk balance should be made by the investigator, with consideration of the totality of information as it pertains to the nature of the toxicity and the degree of clinical benefit a given patient may be experiencing prior to further administration of MPDL3280A. MPDL3280A should be permanently discontinued in patients with life-threatening irAEs.

6.2 Guidelines for Dosage Modification and Treatment Interruption or Discontinuation

There will be no dose reduction for MPDL3280A in this study. Patients may temporarily suspend study treatment for up to 42 days beyond the scheduled date of delayed infusion if study drug-related toxicity requiring dose suspension is experienced. If MPDL3280A is held because of AEs for > 42 days beyond the scheduled date of infusion, the patient will be discontinued from MPDL3280A and will be followed for safety and efficacy.

Dose interruptions for reasons other than toxicity, such as surgical procedures, may be allowed. The acceptable length of interruption will be at the discretion of the Sponsor.

Any toxicities associated or possibly associated with MPDL3280A treatment should be managed according to standard medical practice. Additional tests, such as autoimmune serology or biopsies, may be used to determine a possible immunogenic etiology. Although most irAEs observed with immunomodulatory agents have been mild and self-limiting, such events should

be recognized early and treated promptly to avoid potential major complications. Discontinuation of MPDL3280A may not have an immediate therapeutic effect, and there is no available antidote for MPDL3280A. In severe cases, immune-related toxicities may be acutely managed with topical corticosteroids, systemic corticosteroids, mycophenolate, or TNFα inhibitors.

Patients should be assessed clinically (including review of laboratory values) for toxicity prior to, during, and after each infusion. If unmanageable toxicity due to MPDL3280A occurs at any time during the study, treatment with MPDL3280A should be discontinued.

Management of hepatitis/transaminitis, colitis, rash, and hypothyroidism are presented below as they have been observed in MPDL3280A studies and are potentially immune related. See Section 5.1.1 for guidelines for the management of infusion-related reactions (see Appendix 5 for precautions for anaphylaxis).

6.2.1 Gastrointestinal Toxicity

Immune-mediated colitis has been associated with the administration of MPDL3280A.

Patients should be advised to inform the investigator if any diarrhea occurs, even if it is mild.

If the event is of significant duration or magnitude or is associated with signs of systemic inflammation or acute phase reactants (e.g. increased CRP or platelet count or bandemia), it is recommended that sigmoidoscopy (or colonoscopy, if appropriate) with colonic biopsy with three to five specimens for standard paraffin block be performed. If possible, one or two biopsy specimens should be snap frozen and stored.

Treatment may be restarted following the resolution of colitis if steroids were not instituted. If the patient is being managed with corticosteroids, the patient will be permanently discontinued from treatment. Table 7 provides a summary of dose modification guidelines for gastrointestinal toxicities.

Table 7. Dose Modification Guidelines for Gastrointestinal Toxicity

Toxicity	Description	Management
Diarrhea	Grade 2 (4–6 stools per day over baseline) < 5 days	Hold MPDL3280A and discontinue NSAIDS (or other medications known to exacerbate colitis).<br>Investigate for etiology. Restart MPDL3280A once at baseline stool frequency.
	Grade 2 (4–6 stools per day over baseline) > 5 days	Hold MPDL3280A and discontinue NSAIDS (or other medications known to exacerbate colitis) while etiology is being investigated. Consider referral to a gastroenterologist.<br>Administer anti-diarrheal agent (e.g., Imodium®).<br>Consider oral budesonide, mesalamine, or 10 mg oral prednisone equivalent per day.<br>Restart MPDL3280A once at baseline stool frequency.
	Abdominal pain<br><br>Blood or mucus in stool<br><br><u>OR</u><br><br>Grade ≥ 3 (≥ 7 stools/day over baseline) with peritoneal signs, ileus, or fever	Hold MPDL3280A and discontinue NSAIDS (or other medications known to exacerbate colitis).<br>Rule out bowel perforation.<br><br>Consider administering prednisone 60 mg/day or equivalent.<br>Taper steroids over 1 month.<br>If steroids are initiated the patient will be permanently discontinued from treatment.<br>Permanently discontinue MPDL3280A for life-threatening, immune-related diarrhea or colitis.
NSAID = nonsteroidal anti-inflammatory drug.		

6.2.2 Hepatotoxicity

Immune-mediated hepatitis has been associated with the administration of MPDL3280A.

While on this study, patients presenting with right upper-quadrant abdominal pain and/or unexplained nausea or vomiting should have LFTs performed immediately and LFTs should be reviewed before administration of the next dose of study drug.
If LFTs increase, neoplastic, concurrent medications, viral hepatitis, and toxic etiologies should be considered and addressed, as appropriate. Imaging of the liver, gall bladder, and biliary tree should be performed to rule out neoplastic or other causes for the increased LFTs. Anti-nuclear antibody, perinuclear anti-neutrophil cytoplasmic antibody, anti-liver-kidney microsomal antibodies, and anti-smooth muscle antibody tests should be performed in an autoimmune etiology is considered.

Patients with LFT abnormalities should be managed according to the guidelines in Table 8.

Table 8. Dose Modification Guidelines for Hepatotoxicity

Toxicity	Description	Management
LFT abnormalities	AST/ALT (> ULN to 3 × ULN) with total bilirubin < 2 × ULN	Continue with the standard monitoring plan (i.e., LFTs every 3 weeks before dosing).
	AST/ALT (> 3 × ULN to < 10 × ULN) with total bilirubin < 2 × ULN	Continue MPDL3280A.<br>Monitor LFTs at least weekly.<br>Consider referral to a hepatologist.
	AST/ALT > 10 × ULN	Hold MPDL3280A.<br>Consider administering IV steroids for 24–48 hours (prednisone 60 mg/day equivalent) followed by an oral prednisone (or equivalent) taper over 2–4 weeks. If LFT results do not decrease within 48 hours after initiation of systemic steroids, addition of an alternative immunosuppressive agent (e.g., mycophenolate or TNFα antagonist) to the corticosteroid regimen may be considered. Monitor LFTs every 48–72 hours until decreasing and then follow weekly.<br>If steroids are initiated the patient will be permanently discontinued from treatment.Permanently discontinue MPDL3280A for life-threatening, immune-related hepatic events.
LFT abnormalities (cont.)	AST/ALT ≥ 3 × ULN with bilirubin > 2 × ULN	Hold MPDL3280A.<br>Consult a hepatologist.<br>Consider administering IV steroids for 24–48 hours (prednisone 60 mg/day equivalent) followed by oral taper over 1 month. If LFTs results do not decrease within 48 hours after initiation of systemic steroids, addition of an alternative immunosuppressive agent (e.g., mycophenolate or TNFα antagonist) to the corticosteroid regimen may be considered. Monitor LFTs every 48–72 hours until decreasing and then follow weekly. If steroids are initiated the patient will be permanently discontinued from treatment.<br>Permanently discontinue MPDL3280A for life-threatening, immune-related hepatic events.
IV = intravenous; LFT = liver function test; TNFα = tumor necrosis factor alpha; ULN = upper limit of normal.		

6.2.3 Dermatologic Toxicity

Treatment-emergent rash has been associated with MPDL3280A. The majority of cases of rash were mild in severity and self-limited, with or without pruritus.

A dermatologist should evaluate persistent and/or severe rash or pruritus. A biopsy should be performed unless contraindicated. Low-grade rash and pruritus immune-related adverse events have been treated with symptomatic therapy (e.g. antihistamines). Topical or parenteral corticosteroids may be required for more severe symptoms.

Dermatologic toxicity and rash should be managed according to the guidelines in Table 9.

Table 9. Dose Modification Guidelines for Dermatologic Toxicity

Toxicity	Description	Management
Dermatologic toxicity/rash (e.g., maculopapular or purpura)	Grade 1: Mild<br>< 10% BSA	Continue MPDL3280A symptomatic therapy with antihistamine PRN.<br>Consider topical steroids and/or other symptomatic therapy (e.g., antihistamines).
	Grade 2: Moderate<br>10%–30% BSA	Continue MPDL3280A. Consider dermatologist referral.<br>Administer topical steroids.<br>Consider higher potency topical steroids if rash is unresolved.
	Grade 3: Severe<br>> 30% BSA	Hold MPDL3280A.<br>Consult dermatologist. Administer oral prednisone 10 mg or equivalent. If the rash is unresolved after 48–72 hours, administer oral prednisone 60 mg or equivalent.<br>If 60 mg of prednisone or equivalent is needed the patient will be permanently discontinued from treatment.<br>Permanently discontinue MPDL3280A for life-threatening, immune-related dermatologic toxicity.
BSA = body surface area; PRN = as needed.		

6.2.4 Endocrine Toxicity

Hypothyroidism has been associated with the administration of MPDL3280A.

Patients with unexplained symptoms such as fatigue, myalgias, impotence, mental status changes, or constipation should be investigated for the presence of thyroid, pituitary, or adrenal endocrinopathies, as well as for hyponatremia or hyperkalemia. An endocrinologist should be consulted if an endocrinopathy is suspected. TSH and free T4 levels should be obtained to determine if thyroid abnormalities are present. TSH, prolactin, and a morning cortisol level will help to differentiate primary adrenal insufficiency from primary pituitary insufficiency.

Hypothyroidism should be managed according to the guidelines in Table 10.

Table 10. Dose Modification Guidelines for Endocrine Toxicity

Toxicity	Description	Management
Hypothyroidism	TSH elevated, asymptomatic	Continue MPDL3280A.<br>Start thyroid-replacement hormone.<br>Monitor TSH weekly.
	TSH elevated, symptomatic	Hold MPDL3280A.<br>Consider referral to an endocrinologist.<br>Restart MPDL3280A when symptoms are

		controlled by thyroid replacement and TSH levels are decreasing.
TSH = thyroid-stimulating hormone.		

6.2.5 Pulmonary Toxicity

Dyspnea, cough, fatigue, hypoxia, pneumonitis, and pulmonary infiltrates have been associated with the administration of MPDL3280A and have primarily been observed in patients with underlying NSCLC.

Mild-to-moderate events of pneumonitis have been associated with MPDL3280A. All pulmonary events should be thoroughly evaluated for other commonly reported etiologies such as pneumonia/infection, lymphangitic carcinomatosis, pulmonary embolism, heart failure, chronic obstructive pulmonary disease (COPD), or pulmonary hypertension and the following should be performed:

- Measurement of oxygen saturation (i.e. arterial blood gas)
- High-resolution CT scan of the chest
- Bronchoscopy with bronchoalveolar lavage
- Pulmonary function tests (diffusing capacity of the lung for carbon monoxide [DL_{CO}])

Patients will be assessed for pulmonary signs and symptoms throughout the study. Patients will also have CT scans of the chest at every tumor assessment.

Pulmonary toxicity should be managed according to the guidelines in Table 11.

Table 11. Dose Modification Guidelines for Pulmonary Toxicity

Toxicity	Description	Management
Pulmonary toxicity	GGO or non-infectious infiltrate in absence of hypoxia, or dyspnea	Hold treatment with MPDL3280A.<br>Re-evaluate after 1 week.<br>If no worsening in GGO/infiltrates and patient still asymptomatic, resume treatment with MPDL3280A.<br>If GGO/infiltrates worsen and patient is still asymptomatic, continue to hold MPDL3280A and refer for bronchoscopy.<br>Consider starting low-dose oral prednisone 10 mg or equivalent.<br>Re-evaluate after 1 week. Resume MPDL3280A if GGO/infiltrates improving.

	Hypoxia or dyspnea in presence of GGO or infiltrate without alternative etiology	Hold MPDL3280A.<br>Consult a pulmonologist. Investigate for other etiologies and consider bronchoscopy. If bronchoscopy is consistent with immune-related etiology, start 60 mg prednisone equivalent per day followed by taper over 2 weeks.<br>If steroids are initiated the patient will be permanently discontinued from treatment<br>Permanently discontinue MPDL3280A for life-threatening, immune-related pulmonary events.
GGO = ground glass opacities.		

6.2.6 Pericardial and Pleural Effusions

Pericardial and pleural involvement with associated effusions is common in patients with NSCLC and have the theoretical potential to be exacerbated by inflammation associated with antitumor immunity following PD-L1 blockade. Patients presenting with dyspnea, chest pain, or unexplained tachycardia should be evaluated for the presence of a pericardial effusion. Patients with preexisting pericardial effusion should be followed closely for pericardial fluid volume measurements and impact on cardiac function. When intervention is required for pericardial or pleural effusions, appropriate workup includes cytology, LDH, glucose, cholesterol, protein concentrations (with pleural effusions), and cell count. For patients with a pericardial effusion causing end-diastolic right ventricular collapse, treatment may be restarted following the placement of a pericardial window, demonstrations of hemodynamic stability, and resolution of right ventricular dysfunction.

6.2.7 Potential Pancreatic Toxicity

Symptoms of abdominal pain associated with elevations of amylase and lipase suggestive of pancreatitis have been associated with the administration of other immunomodulatory agents (ipilimumab). The differential diagnosis of acute abdominal pain should be pancreatitis. Appropriate workup should include an evaluation for obstruction, as well as serum amylase and lipase tests.

6.2.8 Potential Eye Toxicity

An ophthalmologist should evaluate visual complaints. Uveitis or episcleritis may be treated with topical corticosteroid eye drops. MPDL3280A should be permanently discontinued for immune-mediated ocular disease that is unresponsive to local immunosuppressive therapy.

Ocular toxicity should be managed according to the guidelines in Table 12.

Table 12. Dose Modification Guidelines for Eye Toxicity

Toxicity	Description	Management

Eye toxicity (autoimmune uveitis, iritis, or episcleritis)	Symptomatic	Hold MPDL3280A.<br>Consult ophthalmologist and start topical corticosteroid eye drops.<br>If steroids are initiated the patient will be permanently discontinued from treatment.

6.3 Management of SAR- Specific Adverse Events

6.3.1 Dose Modification

Patients with peripherally located tumors for whom dose volume constraints cannot be met can be treated with the centrally located tumor regimen of 50 Gy in 5 fractions if that will allow achievement of mandatory dose volume constraints. No other dose modifications for SAR will be permitted. If the dose volume constraints listed in Tables 5 and 6 cannot be achieved, patients should not be treated on protocol. No mid-treatment dose modifications are allowed, as SAR-induced side effects typically do not manifest during a course of treatment. Any patient manifesting grade 3 or higher pulmonary toxicity during the course of SAR treatment will be discontinued from protocol therapy.

6.3.2 Lung Injury

Radiation pneumonitis is a sub-acute (weeks to months from treatment) inflammation of the end bronchioles and alveoli. Radiation fibrosis is a late manifestation of radiation injury to the irradiated lung. Given the small amount of lung that is typically included in the SBRT portals, lung toxicity has not been as dose-limiting as in conventionally fractionated large field RT, but it is nevertheless seen, can be symptomatic, and may be confused with other causes of respiratory deterioration, including infections, and tumor recurrence. The infiltrate on chest x-ray should include the area treated to high dose, but may extend outside of these regions. The infiltrates may characteristically correspond to the regions of high-dose radiation, but may also be ill-defined. Patients reporting symptoms as above will be promptly evaluated and treated.

Appropriate workup for pulmonary adverse events is described above in 6.2.5 and reiterated here. It should include the following as appropriate, as well as ruling out alternative causes (e.g., lymphangitic carcinomatosis, infection, heart failure, pulmonary embolism, chronic obstructive pulmonary disease, or pulmonary hypertension):

- Measurement of oxygen saturation (i.e. arterial blood gas)
- High-resolution CT scan of the chest
- Bronchoscopy with bronchoalveolar lavage
- Pulmonary function tests (diffusing capacity of the lung for carbon monoxide [DLCO])

Pulmonary function testing and CT with a pulmonary embolism protocol may also be helpful in the diagnostic evaluation. Consultation with a pulmonologist is appropriate for a suspected lung immune-related adverse event.

Mild radiation pneumonitis may be treated with nonsteroidal anti-inflammatory agents or steroid inhalers. More significant pneumonitis will be treated with systemic steroids, bronchodilators,

and pulmonary toilet. For severe symptoms, prednisone 60 mg or equivalent may be required to control initial symptoms, and the dose should be gradually tapered over 4-6 weeks. Prednisone (for oral administration) or methylprednisolone (for IV administration) are the corticosteroids of choice in the treatment of severe pulmonary toxicity. Supra- and concurrent infections should be treated with antibiotics. Consideration of prophylaxis of opportunistic infections should be considered in immunocompromised patients.

Patients will be assessed for pulmonary signs and symptoms throughout the study. Patients will also have CT scans of the chest at every tumor assessment.

Effort will be made to distinguish between radiation pneumonitis and pulmonary toxicity caused by MPDL3280A, including distribution of CT abnormalities in the lung (within or outside the target volume) and timing of onset of symptoms in relation to deliver of SAR and MPDL3280A.

6.3.3 Gastrointestinal/Esophageal Injury

The radiation effects on the esophagus can be acute: esophagitis (i.e., dysphagia, causing pain on swallowing, typically relatively soon after RT course is completed, and typically resolves on its own within days to a week or longer), or chronic, typically manifesting with dysphagia due to stenosis, or esophageal ulceration, with perforation in the extreme cases.

6.3.4 Central Airway/Bronchial Injury

Bronchial injury with subsequent focal collapse of lung may impair overall pulmonary status and may hamper further assessment of tumor response as the collapsed lung approximates treated tumor. The consequences of bronchial toxicity, e.g., cough, dyspnea, hypoxia, impairment of pulmonary function test parameters, pleural effusion or pleuritic pain (associated with collapse), should all be graded according to the Common Terminology Criteria for Adverse Events (CTCAE), v. 4

7.0 CONCOMITANT AND EXCLUDED THERAPIES

7.1 Concomitant Therapy

The patient must be told to notify the investigational site about any new medications he/she takes after the start of the study drug. All medications (other than study drug) and significant non-drug therapies (including blood transfusions) administered during the study should be recorded.

Concomitant therapy includes any prescription medications or over-the-counter preparations used by a patient between the 7 days preceding the screening evaluation and the treatment discontinuation visit.

Patients who experience infusion-associated symptoms may be treated symptomatically with antipyretics (ibuprofen preferred), diphenhydramine, and/or cimetidine or another H2 receptor antagonist, as per standard practice. Serious infusion-associated events manifested by dyspnea, hypotension, wheezing, bronchospasm, tachycardia, reduced oxygen saturation, or respiratory

distress should be managed with supportive therapies as clinically indicated (e.g. supplemental oxygen and β_2-adrenergic agonists).

Systemic corticosteroids and TNFα inhibitors may attenuate potential beneficial immunologic effects of treatment with MPDL3280A but may be administered at the discretion of the treating physician. If feasible, alternatives to corticosteroids should be considered. Premedication may be administered for Cycles ≥ 2 at the discretion of the treating physician. The use of inhaled corticosteroids and mineralocorticoids (e.g., fludrocortisone) for patients with orthostatic hypotension or adrenocortical insufficiency is allowed. Megastrol administered as appetite stimulant is acceptable while the patient is enrolled in the study.

Patients who use oral contraceptives, hormone-replacement therapy, prophylactic or therapeutic anticoagulation therapy (such as low-molecular-weight heparin or warfarin at a stable dose level), or other allowed maintenance therapy should continue their use. Males and females of reproductive potential should use highly effective means of contraception.

7.2 Excluded Therapy

Any concomitant therapy intended for the treatment of cancer, whether health authority-approved or experimental, is prohibited. This includes but is not limited to the following:

- Chemotherapy, hormonal therapy, immunotherapy, radiotherapy, investigational agents, or herbal therapy
  - After Cycle 1, certain forms of radiotherapy may be considered for pain palliation if patients are deriving benefit (e.g., treatment of known bony metastases); MPDL3280A administration may be suspended during radiotherapy.

It is strongly recommended that:

- Traditional herbal medicines not be administered because the ingredients of many herbal medicines are not fully studied and their use may result in unanticipated drug-drug interactions that may cause, or confound assessment of, toxicity
- The use of a RANKL inhibitor (denosumab) be discontinued during the study; this agent could potentially alter the activity and the safety of MPDL3280A

Initiation or increased dose of granulocyte colony-stimulating factors (e.g., granulocyte colony-stimulating factor, granulocyte/macrophage colony-stimulating factor, and/or pegfilgrastim) is prohibited for patients with solid malignancies.

Patients are not allowed to receive immunostimulatory agents, including but not limited to IFN-α, IFN-γ, or IL-2, during the entire study. These agents, in combination with MPDL3280A, could potentially increase the risk for autoimmune conditions.

Patients should also not be receiving immunosuppressive medications, including but not limited to cyclophosphamide, azathioprine, methotrexate, and thalidomide. These agents could potentially alter the activity and the safety of MPDL3280A. Systemic corticosteroids and anti-TNFα agents may attenuate potential beneficial immunologic effects of treatment with

MPDL3280A but may be administered at the discretion of the treating physician. If feasible, alternatives to these agents should be considered.

In addition, all patients (including those who discontinue the study early) should not receive other immunostimulatory agents for 10 weeks after the last dose of MPDL3280A.

8.0 TREATMENT DURATION

Patients will complete therapy unless one of the following criteria applies:

- Disease progression
- Intercurrent illness that prevents further administration of treatment
- Unacceptable adverse events
- Patient withdrawal from study (patient choice)
- Pregnancy
- Delay in treatment > 42 days due to toxicity
- Failure of patient to adhere to study requirements

8.1 Duration of Follow Up

All patients will be followed for 30 days after the last dose of treatment with the agent under this IND or until all treatment related clinical significant toxicities resolve to ≤ grade 1. Adverse events with attribution of possible, probable or definite will be reported following guidelines for adverse event reporting and all SAEs will be reported for 30 days after the last dose. Patients will be followed with a CT chest, labs and clinic visit every 3 months years 1-2 then every 6 months years 3-5.

9.0 STUDY ASSESSMENTS AND MONITORING

The study calendar lists all the study assessments and their scheduled times. All data obtained from these assessments will be recorded in the study specific case report forms that will be generated prior to study activation. See Appendix 2 for study registration guidelines and Appendix 3 for data submission schedule.

Assessment Window (Days)	Screening	All Cycles	EOT	Follow Up
	Days - 28 to -1	Day 1[a] (± 3 Days for Cycles ≥ 2)	≤ 30 Days after Last Dose	
Signed Informed Consent Form(s)	X			
Review of eligibility criteria	X			
Medical History	X	X	X	X
Concomitant medications	X	X	X	X
Physical examination	X	X	X	X
ECOG performance status	X	X	X	X
Vital signs	X	X	X	X
Weight	X	X	X	X
Height	X			
Tumor assessment	X[g]	Every 6 weeks[g]	X[b]	X[b]
PFT	X[e]			
Hematology	X	X	X	X
Serum chemistry	X	X	X	X
INR/PTT	X[f]			
EBV, HIV, HBV, HCV serology	X			
C-reactive protein testing	X	X	X	
TSH, free T3, free T4	X	X	X	
Serum pregnancy test if applicable	X	X	X	
Adverse events	X	X (assessed weekly during the DLT period[h])	X	X
Blood samples for immune assays[d]	X	X	X	
Archival/screening FFPE tumor tissue specimen or 16 unstained slides[d]	X			
Stool sample[d]	X	End of Cycle 2	X (treatment discontinuation)	
MPDL3280A infusion		X		
SAR		X (cycle 3)		
Optional post treatment biopsy		X[c]		

[a] If blood work is obtained within 14 days of Cycle 1 it does not have to be repeated.
[b] Patients will be followed with a CT chest, labs and clinic visit every 3 months years 1-2 then every 6 months years 3-5
[c] Patients may undergo a post treatment biopsy at the end of cycle 6 if deemed safe by the study team and the patient agrees
[d] See the correlative study section for detailed information (section 11.0)
[e] PFTs may be obtained within 3 months of registrations.
[f] Only for patients on anticoagulation.
[g] Scans will be performed at baseline within 8 weeks of registration, and on treatment scans will be performed Day 1 (±1 week) everv 6 weeks.
[h] Patients will be assessed by a clinic visit or by telephone if a patient cannot make an appointment.

Abbreviations: ECOG-Eastern Cooperative Oncology Group; PFT=pulmonary function test; INR/PTT=international normalized ratio/partial thromboplastin time; EBV-Epstein Barr Virus; HIV-Human Immunodeficiency Virus; HBV-Hepatitis B Virus; HCV-Hepatitis C Virus; TSH-Thyroid Stimulating Hormone; T3-Triiodothyronine; T4-Thyroxine; DLT-Dose Limiting Toxicity; FFPE-Formalin-fixed, paraffin-embedded; SAR- Stereotactic Ablative Radiotherapy ; CT-Computerized tomography

10.0 ASSESSMENT TYPES

10.1 Efficacy

Response and progression will be evaluated in this study using the new international criteria proposed by the revised Response Evaluation Criteria in Solid Tumors (RECIST) guideline (version 1.1) (25) as well as irRECIST criteria. Additionally, imaging will be reviewed by a radiologist in conjunction with a radiation oncologist to help distinguish tumor progression from post-SAR scarring. Changes in the largest diameter (unidimensional measurement) of the tumor lesions and the shortest diameter in the case of malignant lymph nodes are used in the RECIST criteria.

Disease Parameters

Measurable Disease
The presence of at least one measurable lesion. If the measurable disease is restricted to a solitary lesion, its neoplastic nature should be confirmed by cytology/histology.

Measurable Lesions
Lesions that can be accurately measured in at least one dimension with longest diameter 20mm using conventional techniques or 10mm with spiral CT scan.

Non-Measurable Lesions
All other lesions, including small lesions (longest diameter <20 mm with conventional techniques or <10 mm with spiral CT scan), i.e., bone lesions, leptomeningeal disease, ascites, pleural/pericardial effusion, inflammatory breast disease, lymphangitis cutis/pulmonis, cystic lesions, and also abdominal masses that are not confirmed and followed by imaging techniques**.**

All measurements should be taken and recorded in metric notation, using a ruler or calipers. All baseline evaluations should be performed as closely as possible to the beginning of treatment and never more than 4 weeks before the beginning of the treatment.

The same method of assessment and the same technique should be used to characterize each identified and reported lesion at baseline and during follow up.

Clinical lesions will only be considered measurable when they are superficial (e.g., skin nodules and palpable lymph nodes). For the case of skin lesions, documentation by color photography, including a ruler to estimate the size of the lesion, is recommended.

Methods of Measurement

CT and MRI are the best currently available and reproducible methods to measure target lesions selected for response assessment. Conventional CT and MRI should be performed with cuts of 10 mm or less in slice thickness contiguously. Spiral CT should be performed using a 5 mm contiguous reconstruction algorithm. This applies to tumors of the chest, abdomen and pelvis. Head and neck tumors and those of extremities usually require specific protocols.

Lesions on chest X-ray are acceptable as measurable lesions when they are clearly defined and surrounded by aerated lung. However, CT is preferable.

When the primary endpoint of the study is objective response evaluation, ultrasound (US) should not be used to measure tumor lesions. It is, however, a possible alternative to clinical measurements of superficial palpable lymph nodes, subcutaneous lesions and thyroid nodules. US might also be useful to confirm the complete disappearance of superficial lesions usually assessed by clinical examination.

The utilization of endoscopy and laparoscopy for objective tumor evaluation has not yet been fully and widely validated. Their uses in this specific context require sophisticated equipment and a high level of expertise that may only be available in some centers. Therefore, the utilization of such techniques for objective tumor response should be restricted to validation purposes in specialized centers. However, such techniques can be useful in confirming complete pathological response when biopsies are obtained.

Cytology and histology can be used to differentiate between PR and CR in rare cases (e.g., after treatment to differentiate between residual benign lesions and residual malignant lesions in tumor types such as germ cell tumors).

Response Criteria

Evaluation of Target Lesions

- **Complete Response (CR):** Disappearance of the target lesion
- **Partial Response (PR):** At least a 30% decrease in the sum of the LD of target lesions, taking as reference the baseline sum LD
- **Progressive Disease (PD):** At least a 20% increase in the sum of the LD of target lesion, or the appearance of one or more new lesions
- **Stable Disease (SD):** Neither sufficient shrinkage to qualify for PR nor sufficient increase to qualify for PD, taking as reference the smallest sum LD since the treatment started

Evaluation of Best Overall Response

The best overall response is the best response recorded from the start of the treatment until disease progression/recurrence (taking as reference for PD the smallest measurements recorded since the treatment started). In general, the patient's best response assignment will depend on the achievement of both measurement and confirmation criteria.

Best Overall Response Evaluation

**Target Lesion**	**New Lesion**	**Overall Response**
CR	No	CR
PR	No	PR
SD	No	SD
PD	Yes or No	PD

Patients with a global deterioration of health status requiring discontinuation of treatment without objective evidence of disease progression at that time should be classified as having "symptomatic deterioration". Every effort should be made to document the objective progression even after discontinuation of treatment.

In some circumstances it may be difficult to distinguish residual disease from normal tissue. When the evaluation of complete response depends on this determination, it is recommended that the residual lesion be investigated (fine needle aspirate/biopsy) to confirm the complete response status.

Immune Related Response Criteria

A growing body of literature indicates that radiographic responses to immunotherapy may have different patterns and kinetics than what would be expected with traditional cytotoxic therapies. To account for these differences we will also characterize radiographic outcomes using the immune related response criteria outlined by Wolchok and colleagues (25). See Appendix 6.

Confirmation

The main goal of confirmation of objective response is to avoid overestimating the response rate observed. In cases where confirmation of response is not feasible, it should be made clear when reporting the outcome of such studies that the responses are not confirmed.

To be assigned a status of PR or CR, changes in tumor measurements must be confirmed by repeat assessments that should be performed no less than 4 weeks after the criteria for response are first met. Longer intervals as determined by the study protocol may also be appropriate.
In the case of SD, follow-up measurements must have met the SD criteria at least once after study entry at a minimum interval (in general, not less than 6-8 weeks) that is defined in the study protocol

Duration of Overall Response

The duration of overall response is measured from the time measurement criteria are met for CR or PR (whichever status is recorded first) until the first date that recurrence or PD is objectively documented, taking as reference for PD the smallest measurements recorded since the treatment started.

Duration of Stable Disease

SD is measured from the start of the treatment until the criteria for disease progression are met, taking as reference the smallest measurements recorded since the treatment started.
The clinical relevance of the duration of SD varies for different tumor types and grades. Therefore, it is highly recommended that the protocol specify the minimal time interval required between two measurements for determination of SD. This time interval should take into account the expected clinical benefit that such a status may bring to the population under study.

Disease Control Rate (DCR)

DCR is defined as the percentage of patients that achieve an objective tumor response or stable disease to therapy.

Progression-Free Survival (PFS)

PFS is defined as the duration of time from start of treatment to time of progression or death, whichever occurs first.

Overall Survival (OS)

OS is defined as the duration of time from the start of treatment to death from any cause.

Reporting of Results

All patients included in the study must be assessed for response to treatment, even if there are major protocol treatment deviations or if they are ineligible. Each patient will be assigned one of the following categories: 1) complete response, 2) partial response, 3) stable disease, 4) progressive disease, 5) early death from malignant disease, 6) early death from toxicity, 7) early death because of other cause, or 9) unknown (not assessable, insufficient data).

All of the patients who met the eligibility criteria should be included in the main analysis of the response rate. Patients in response categories 4-9 should be considered as failing to respond to treatment (disease progression). Thus, an incorrect treatment schedule or drug administration does not result in exclusion from the analysis of the response rate. Precise definitions for categories 4-9 will be protocol specific.

All conclusions should be based on all eligible patients. Subanalyses may then be performed on the basis of a subset of patients, excluding those for whom major protocol deviations have been identified (e.g., early death due to other reasons, early discontinuation of treatment, major protocol violations, etc.). However, these subanalyses may not serve as the basis for drawing conclusions concerning treatment efficacy, and the reasons for excluding patients from the analysis should be clearly reported.

The 95% confidence intervals should be provided.

10.2 Safety

Safety assessments will consist of monitoring and recording all adverse events, including serious adverse events, the monitoring of hematology, blood chemistry, coagulation parameters, urinalysis and the regular monitoring of vital signs, and physical condition as shown in corresponding tables. For details on AE collection and reporting, refer to the Safety section in the protocol.

11.0 CORRELATIVE SCIENCE

11.1 Rationale

One of the major shortcomings of immunotherapy trials has been the lack of in depth correlative studies to help identify the mechanism of action of these therapies, identify biomarkers of response, and provide a foundation for further improving these approaches. To address this we plan to perform in depth immunological analysis of patient blood and tumor samples. Pre- and post-treatment blood and tumor biopsies (when available) will be obtained as outlined in the study calendar. We will evaluate immunologic changes systemically and in the tumor and tumor microenvironment within patient's pre to post therapy and across the cohort of patients to identify predictive biomarkers and elucidate the mechanistic immunologic effects of therapy. For further details, refer to Appendix 4. Correlative studies will include (in order of priority):

Blood
- FACS for quantification, immunophenotyping, and functional assessment of PBMCs
- qPCR (quantitative polymerase chain reaction) evaluation of immune gene signatures including: cytokines, T-cell activation markers, immunosuppressive enzymes and molecules (IDO [indoleamine-pyrrole 2,3-dioxygenase], arginase, CTLA-4, PD-1/PD-L1), macrophage polarization, etc.
- Luminex evaluation of plasma for systemic cytokine / chemokine signatures
- T-cell receptor (TCR) deep sequencing to determine clonal expansion of T-cells in the systemic circulation
- Other studies as deemed feasible and informative by the principal investigators

Tissue
- IHC to determine PD-1, PD-L1, CD8, CD4, and FOXP3 expression
- qPCR evaluation of the tumor microenvironment gene signatures including: cytokines, T-cell activation markers, immunosuppressive enzymes and molecules (IDO, arginase, CTLA4, PD-1/PD-L1), macrophage polarization, etc.
- Other studies as deemed feasible and informative by the principal investigators

Stool
- A growing body of evidence suggests that the gut microbiome is an important contributor to the overall immune and inflammatory status of an individual. The intestinal flora can produce bioactive molecules such as short chain fatty acids and tryptophan metabolites which can have profound effects on immune responses [51]. The microbiome could influence the outcome of immunotherapy and play a role in regulating the anti-cancer immune response [52].
- Stool microbiome analysis will be performed on stool samples as a fee for service by MicroTrek.

A comprehensive analysis of tumor, blood, and stool samples will provide valuable insight into potential biomarkers and into the mechanism of action of our combined therapy.

11.2 Study Design and Methodology

We have extensive expertise in all of the techniques described at the UC Davis Laboratory of Tumor Immunology. Blood samples will be separated into PBMCs and plasma and stored at -80 for batched analysis. PBMCs will be stained for FACS analysis using well characterized antibodies against markers such as CD45, CD3, CD4, CD8, FOXP3, Granzyme B, Interferon gamma, tumor necrosis factor (TNF) alpha, PD-1, etc. Results will be analyzed using a BD Fortessa multi-color flow cytometer and Flowjo software. An aliquot of PBMCs will also be set aside for RNA isolation and batched analysis of gene expression. mRNA (messenger ribonucleic acid) will be extracted using RNeasy kits (Qiagen) and reverse transcribed to cDNA. Cellular RNA will be analyzed by qRT-PCR (real-time reverse transcription-polymerase chain reaction) using verified primers to investigate the expression of genes including IDO, arginase, iNOS (inducible Nitric oxide synthases), CTLA-4, PD-1, PD-L1 and others. To determine if certain clones of T cells are preferentially expanding after therapy, an aliquot of PBMCs will also be used for TCR deep sequencing which will be performed in conjunction with the Beijing Genomics Institute (which is located at UC Davis adjacent to the Laboratory of Tumor Immunology). Plasma will be evaluated for systemic cytokine and chemokine signatures using Luminex technology. Markers such as IL-2, IL-6, IL-10, IL-12p70, GM-CSF (granulocyte-macrophage colony-stimulating factor), TNF alpha, IFN (interferon) gamma, CXCL10 (C-X-C motif chemokine 10), RANTES (regulated on activation, normal T cell expressed and secreted), MIP1 (Macrophage Inflammatory Protein) alpha, MIP1 beta and others will be evaluated using the Bio-Rad human 27-plex panel and TGF-beta will be evaluated using the Bio-Rad TGF-beta 3-plex panel. These multi-plexed suspension arrays will be analyzed using a Bio-Plex 200 system and Bio-Plex Manager software. Tissue biopsies will be formalin fixed and paraffin embedded using standard protocols. Tissue will be analyzed by IHC staining for PD-1, PD-L1, FOXP3, CD8, and CD4 markers. If sufficient biopsy material is available then a tissue sample will be stored in RNA later for batched analysis by qPCR as described above.

11.3 All Study Variables

The panel of study endpoints and how they will be measured are described in the below table.

**Table 6. Study Variables and Measurements**		
Study Variable	Measurement	Comment
Feasibility	Completion of planned treatment	
Safety	CTCAE Version 4.0	
Dose Limiting Toxicity	CTCAE version 4.0	The DLT period is defined as the first 9 weeks of study treatment. Using CTCAE version 4.0 any Grade 3 or 4 immune related AE **OR** any other grade 3-4 AE that does not resolve within 14 days of onset.
Maximum Tolerated Dose	CTCAE version 4.0	One dose level below that in which ≥ 2 of 6 patients develops DLT OR dose level 3.
ORR	RECIST 1.1	
DFS	RECIST 1.1	
Blood:<br>PBMC Immunophenotyping	FACS	
PBMC Gene Signature	qPCR	
T cell clonal expansion	TCR spectratyping	
Systemic cytokine signature	Luminex	
Tissue Marker Expression	IHC	
Tumor Microenvironment Gene Signature	qPCR	
Abbreviations: CTCAE v4-Common Terminology Criteria for Adverse Events Version 4; DLT-Dose Limiting Toxicity; AE-Adverse Event; ORR-Objective Response Rate; RECIST- Response Evaluation Criteria in Solid Tumors; PBMC- Peripheral Blood Mononuclear Cell; FACS-Fluorescence-activated Cell Sorting; qPCR-Quantitative Polymerase Chain Reaction; TCR-T cell antigen receptor; IHC-Immunohistochemistry;		

12.0 DATA AND STATISTICAL ANALYSIS PLAN

12.1 Sample Size

For the dose escalation phase a traditional 3 + 3 design will be used with the goal of determining the MTD through DLT assessment. Per design each cohort will consist of 3-6 patients. The number of patients will depend on the occurrence of DLT (Table 3). We estimate a minimum of 6 patients and a maximum of 18 patients (6 patients/cohort) would be enrolled. Fifteen patients will be enrolled in the expansion phase. The primary endpoint of this study is to determine the MTD of MPDL3280A when combined with SAR and to further assess safety at the proposed MTD, with preliminary assessment of efficacy in preparation for a phase II trial. Therefore the sample size was not determined from power analysis, but from the dose-escalation design combined with a feasible dose expansion cohort.

With six patients treated at the MTD in the dose-escalation phase and an additional 15 in the dose-expansion phase, we will have a total of 21 patients available for assessing safety (primary outcome) and providing preliminary estimates of efficacy (secondary outcomes). With 21 patients, we will have an 89% chance of seeing at least one example of any DLT occurring in 10% or more of patients, and an 81% chance of seeing at least one example of any DLT occurring in 7.5% or more of patients.

12.2 Statistical Analysis Plan

Descriptive statistics will be used. All data will be summarized by dose cohort, dose expansion and overall subject population.

12.3 Safety

The adverse events observed will be summarized as frequency, proportion of patients, and exact 95% confidence interval for proportion, categorized by type (organ affected or laboratory determination), severity by CTCAE v4 and nadir or maximum values for the laboratory measures), time of onset (i.e. course number), duration, and reversibility or outcome. Tables will be created to summarize these toxicities by dose and course.

12.4 Efficacy

As a secondary endpoint, all responses will be reported using RECIST 1.1 definitions [53]. Because of the potential heterogeneity of the patients, all results will be considered preliminary and hypothesis generating for future studies. Response rate among will be summarized by exact binomial confidence intervals. Disease free survival will be summarized with Kaplan-Meier plots to describe the outcome of patients treated on this protocol. The median DFS time will be estimated using standard life table methods.

12.5 Subject Course

Information regarding the subject's course such as completing the study treatment, dose delays, premature discontinuation and major protocol violation will be tabulated and summarized.

12.6 Correlative Laboratory Markers

Correlative biomarker endpoints are exploratory and hypothesis generating. Descriptive statistics will be applied to characterize differences within a given patient pre-treatment to post-treatment and across the cohort of patients. Changes in the correlative endpoints will be evaluated using a two-tailed paired student's T-test. To provide a preliminary estimate of the predictive or prognostic value of the various correlative endpoints for DFS we will be using multiple regression analysis with ordinal category for response (generalized linear models) or time to event (proportional hazards survival analysis) as the outcome.

13.0 SAFETY REPORTING OF ADVERSE EVENTS

13.1 Assessment of Safety

Safety assessments will consist of monitoring and reporting adverse events (AEs) and serious adverse events (SAEs) that are considered related to MPDL3280A, all events of death, and any study specific issue of concern.

13.2 Risks Associated with MPDL3280A

The PD-L1/PD-1 pathway is involved in peripheral tolerance; therefore, such therapy may increase the risk of immune-related AEs, specifically the induction or enhancement of autoimmune conditions. AEs with potentially immune-related causes, including rash,

hypothyroidism, hepatitis/transaminitis, colitis, myositis, and myasthenia gravis, have been observed in Study PCD4989g.

Although most immune-related AEs observed with immunomodulatory agents have been mild and self-limiting, such events should be recognized early and treated promptly to avoid potential major complications.

13.3 Adverse Events

An AE is any unfavorable and unintended sign, symptom, or disease temporally associated with the use of an investigational medicinal product (IMP) or other protocol-imposed intervention, regardless of attribution.
This includes the following:

- AEs not previously observed in the subject that emerge during the protocol-specified AE reporting period, including signs or symptoms associated Stage IV NSCLC that were not present prior to the AE reporting period.
- Complications that occur as a result of protocol-mandated interventions (e.g., invasive procedures such as cardiac catheterizations).
- If applicable, AEs that occur prior to assignment of study treatment associated with medication washout, no treatment run-in, or other protocol-mandated intervention.
- Preexisting medical conditions (other than the condition being studied) judged by the investigator to have worsened in severity or frequency or changed in character during the protocol-specified AE reporting period.

13.3.1 Serious Adverse Events

An AE should be classified as an SAE if the following criteria are met:

- It results in death (i.e., the AE actually causes or leads to death).
- It is life threatening (i.e., the AE, in the view of the investigator, places the subject at immediate risk of death. It does not include an AE that, had it occurred in a more severe form, might have caused death.).
- It requires or prolongs inpatient hospitalization.
- It results in persistent or significant disability/incapacity (i.e., the AE results in substantial disruption of the subject’s ability to conduct normal life functions).
- It results in a congenital anomaly/birth defect in a neonate/infant born to a mother exposed to the IMP.
- It is considered a significant medical event by the investigator based on medical judgment (e.g., may jeopardize the subject or may require medical/surgical intervention to prevent one of the outcomes listed above).

13.4 Methods and Timing for Assessing and Recording Safety Variables

The investigator is responsible for ensuring that all AEs and SAEs that are observed or reported during the study are collected and reported to the FDA, appropriate IRB(s), and Genentech, Inc. in accordance with CFR 312.32 (IND Safety Reports).

13.4.1 Adverse Event Reporting Period

The study period during which all AEs and SAEs must be reported begins after informed consent is obtained and initiation of study treatment and ends 30 days following the last administration of study treatment or study discontinuation/termination, whichever is earlier. After this period, investigators should only report SAEs that are attributed to prior study treatment.

13.4.2 Assessment of Adverse Events

All AEs and SAEs whether volunteered by the subject, discovered by study personnel during questioning, or detected through physical examination, laboratory test, or other means will be reported appropriately. Each reported AE or SAE will be described by its duration (i.e., start and end dates), regulatory seriousness criteria if applicable, suspected relationship to the MPDL3280A and radiotherapy (see following guidance), and actions taken.

To ensure consistency of AE and SAE causality assessments, investigators should apply the following general guideline:

Yes (Possibly, Probably, or Definitely)
There is a plausible temporal relationship between the onset of the AE and administration of the MPDL3280A, and the AE cannot be readily explained by the subject’s clinical state, intercurrent illness, or concomitant therapies; and/or the AE follows a known pattern of response to the MPDL3280A; and/or the AE abates or resolves upon discontinuation of the MPDL3280A or dose reduction and, if applicable, reappears upon re-challenge.

No (Unlikely, Unrelated)
Evidence exists that the AE has an etiology other than the MPDL3280A (e.g., preexisting medical condition, underlying disease, intercurrent illness, or concomitant medication); and/or the AE has no plausible temporal relationship to MPDL3280A administration (e.g., cancer diagnosed 2 days after first dose of study drug).

Expected adverse events are those adverse events that are listed or characterized in the Package Insert or current Investigator Brochure.

Unexpected adverse events are those not listed in the Package Insert (P.I.) or current Investigator Brochure (I.B.) or not identified. This includes adverse events for which the specificity or severity is not consistent with the description in the P.I. or I.B. For example, under this definition, hepatic necrosis would be unexpected if the P.I. or I.B. only referred to elevated hepatic enzymes or hepatitis.

13.5 Procedures for Eliciting, Recording, and Reporting Adverse Events

13.5.1 Eliciting Adverse Events

A consistent methodology for eliciting AEs at all subject evaluation time points should be adopted. Examples of non-directive questions include:

- "How have you felt since your last clinical visit?"
- "Have you had any new or changed health problems since you were last here?"

13.5.2 Specific Instructions for Recording Adverse Events

Investigators should use correct medical terminology/concepts when reporting AEs or SAEs. Avoid colloquialisms and abbreviations.

a. Diagnosis vs. Signs and Symptoms
If known at the time of reporting, a diagnosis should be reported rather than individual signs and symptoms (e.g., record only liver failure or hepatitis rather than jaundice, asterixis, and elevated transaminases). However, if a constellation of signs and/or symptoms cannot be medically characterized as a single diagnosis or syndrome at the time of reporting, it is ok to report the information that is currently available. If a diagnosis is subsequently established, it should be reported as follow-up information.

b. Deaths
All deaths that occur during the protocol-specified AE reporting period (see Section 13.2.1), regardless of attribution, will be reported to the appropriate parties. When recording a death, the event or condition that caused or contributed to the fatal outcome should be reported as the single medical concept. If the cause of death is unknown and cannot be ascertained at the time of reporting, report "Unexplained Death".

c. Preexisting Medical Conditions
A preexisting medical condition is one that is present at the start of the study. Such conditions should be reported as medical and surgical history. A preexisting medical condition should be re-assessed throughout the trial and reported as an AE or SAE only if the frequency, severity, or character of the condition worsens during the study. When reporting such events, it is important to convey the concept that the preexisting condition has changed by including applicable descriptors (e.g., "more frequent headaches").

d. Hospitalizations for Medical or Surgical Procedures
Any AE that results in hospitalization or prolonged hospitalization should be documented and reported as an SAE. If a subject is hospitalized to undergo a medical or surgical procedure as a result of an AE, the event responsible for the procedure, not the procedure itself, should be reported as the SAE. For example, if a subject is hospitalized to undergo coronary bypass surgery, record the heart condition that necessitated the bypass as the SAE.

Hospitalizations for the following reasons do not require reporting:

- Hospitalization or prolonged hospitalization for diagnostic or elective surgical procedures for preexisting conditions
- Hospitalization or prolonged hospitalization required to allow efficacy measurement for the study or
- Hospitalization or prolonged hospitalization for scheduled therapy of the target disease of the study.

e. Pregnancy

If a female subject becomes pregnant while receiving investigational therapy or within 120 days after the last dose of study drug, a report should be completed and expeditiously submitted to the Genentech, Inc. Follow-up to obtain the outcome of the pregnancy should also occur. Abortion, whether accidental, therapeutic, or spontaneous, should always be classified as serious, and expeditiously reported as an SAE. Similarly, any congenital anomaly/birth defect in a child born to a female subject exposed to the MPDL3280A should be reported as an SAE. Male patients will be instructed through the ICF to immediately inform the investigator if their partner becomes pregnant during the study or within 90 days after completing treatment with MPDL3280A. Male patients who received study treatment should not attempt to father a child until 90 days after the last dose of MPDL3280A. An investigator who is contacted by the male patient or his pregnant partner may provide information on the risks of the pregnancy and the possible effects on the fetus, to support an informed decision in cooperation with the treating physician and/or obstetrician.

f. Post-Study Adverse Events

The investigator should expeditiously report any SAE occurring after a subject has completed or discontinued study participation if attributed to prior MPDL3280A exposure. If the investigator should become aware of the development of cancer or a congenital anomaly in a subsequently conceived offspring of a female subject who participated in the study, this should be reported as an SAE.

g. Reconciliation

The Sponsor-Investigator agrees to conduct reconciliation for the product. Genentech and the Sponsor-Investigator will agree to the reconciliation periodicity and format, but agree at minimum to exchange monthly line listings of cases received by the other party. If discrepancies are identified, the Sponsor-Investigator and Genentech will cooperate in resolving the discrepancies. The responsible individuals for each party shall handle the matter on a case-by-case basis until satisfactory resolution.

h. AEs of Special Interest (AESIs)

AEs of Special Interest are defined as a potential safety problem, identified as a result of safety monitoring of the Product. The following AEs are considered of special interest and must be reported to Genentech Drug Safety expeditiously, irrespective of regulatory seriousness criteria.

The MPDL3280A Events of Special Interest are:

- Cases of potential drug-induced liver injury that include an elevated ALT or AST in combination with either an elevated bilirubin or clinical jaundice, as defined by Hy's Law and based on the following observations:
    - Treatment-emergent ALT or AST > 3 x ULN (or > 3 x baseline value in disease states where LFTs may be elevated at baseline) in combination with total bilirubin > 2 x ULN (of which ≥ 35% is direct bilirubin)
    - Treatment-emergent ALT or AST > 3 x ULN (or > 3 x baseline value in disease states where LFTs may be elevated at baseline) in combination with clinical jaundice.
- Suspected transmission of an infectious agent by the study treatment, as defined below
    - Any organism, virus, or infectious particle (e.g., prion protein transmitting transmissible spongiform encephalopathy), pathogenic or non-pathogenic, is considered an infectious agent. A transmission of an infectious agent may be suspected from clinical symptoms or laboratory findings that indicate an infection in a patient exposed to a medicinal product. This term applies only when a contamination of study treatment is suspected.
- Pneumonitis
- Colitis
- Endocrinopathies: diabetes mellitus, pancreatitis, adrenal insufficiency, hyperthyroidism, and hypophysitis
- Hepatitis, including AST or ALT > 10xULN
- Systemic lupus erythematosus
- Neurological disorders: Guillain-Barré syndrome, myasthenic syndrome or myasthenia gravis, and meningoencephalitis
- Events suggestive of hypersensitivity, infusion-related reactions, cytokine release syndrome, influenza-like illness, systemic inflammatory response syndrome, and systemic immune activation
- Nephritis
- Ocular toxicities (e.g., uveitis, retinitis, optic neuritis)
- Myositis
- Myopathies, including rhabdomyolysis
- Grade > 2 cardiac disorders (e.g., atrial fibrillation, myocarditis, pericarditis)
- Vasculitis
- Autoimmune hemolytic anemia
- Severe cutaneous reactions (e.g., Stevens-Johnson syndrome, dermatitis bullous, toxic epidermal necrolysis)

i. SAE Reporting

Investigators must report all SAEs to Genentech within the timelines described below. The completed MedWatch/case report should be faxed immediately upon completion to Genentech Drug Safety (Appendix 7) at:

(650) 225-4682
OR
(650) 225-4630

- Relevant follow-up information should be submitted to Genentech Drug Safety as soon as it becomes available.
- Serious AE reports, AEs of Special Interest (regardless of causality) and pregnancies will be transmitted to Genentech within 1 business day of the Awareness Date.
- Additional Reporting Requirements to Genentech include the following:
- All Non-serious Adverse Events originating from the Study will be forwarded in a quarterly report to Genentech.

In addition to SAEs, pregnancy reports and AESIs, the following Special Situations Reports should be collected and transmitted to Genentech/Roche even in the absence of an Adverse Event within thirty (30) calendar days:

- Data related to product usage during pregnancy or breastfeeding
- Data related to overdose, abuse, misuse, inadvertent/erroneous administration, medication error or occupational exposure, with or without association with an AE/SAE unless otherwise specified in the protocol
- Lack of therapeutic efficacy

Note: Investigators should also report events to their IRB as required.

13.6 MedWatch 3500A Reporting Guidelines

In addition to completing appropriate patient demographic and suspect medication information, the report should include the following information within the Event Description (section 5) of the MedWatch 3500A form:

- Protocol description (and number, if assigned)
- Description of event, severity, treatment, and outcome if known
- Supportive laboratory results and diagnostics
- Investigator’s assessment of the relationship of the adverse event to each investigational product and suspect medication

Follow-up Information

- Additional information may be added to a previously submitted report by any of the following methods:
- Adding to the original MedWatch 3500A report and submitting it as follow-up

- Adding supplemental summary information and submitting it as follow-up with the original MedWatch 3500A form
- Summarizing new information and faxing it with a cover letter including patient identifiers (i.e. D.O.B. initial, patient number), protocol description and number, if assigned, brief adverse event description, and notation that additional or follow-up information is being submitted (The patient identifiers are important so that the new information is added to the correct initial report)

Occasionally Genentech may contact the reporter for additional information, clarification, or current status of the patient for whom and adverse event was reported. For questions regarding SAE reporting, you may contact the Genentech Drug Safety representative noted above or the MSL assigned to the study. Relevant follow-up information should be submitted to Genentech Drug Safety as soon as it becomes available and/or upon request.

MedWatch 3500A (Mandatory Reporting) form is available at
http://www.fda.gov/medwatch/getforms.html

13.7 Additional Reporting Requirements for IND Holders

For Investigator-Sponsored IND Studies, some additional reporting requirements for the FDA apply in accordance with the guidance set forth in 21 CFR § 600.80.

Events meeting the following criteria need to be submitted to the Food and Drug Administration (FDA) as expedited IND Safety Reports according to the following guidance and timelines:

7 Calendar Day Telephone or Fax Report:

The Investigator is required to notify the FDA of any fatal or life-threatening adverse event that is unexpected and assessed by the investigator to be possibly related to the use of MPDL3280A. An unexpected adverse event is one that is not already described in the MPDL3280A Investigator Brochure. Such reports are to be telephoned or faxed to the FDA and Genentech within 7 calendar days of first learning of the event.

15 Calendar Day Written Report

The Investigator is also required to notify the FDA and all participating investigators, in a written IND Safety Report, of any serious, unexpected AE that is considered reasonably or possibly related to the use of MPDL3280A. An unexpected adverse event is one that is not already described in the MPDL3280A investigator brochure.

Written IND Safety reports should include an Analysis of Similar Events in accordance with regulation 21 CFR § 312.32. All safety reports previously filed by the investigator with the IND concerning similar events should be analyzed and the significance of the new report in light of the previous, similar reports commented on.

Written IND safety reports with Analysis of Similar Events are to be submitted to the FDA, Genentech, and all participating investigators within 15 calendar days of first learning of the event. The FDA prefers these reports on a MedWatch 3500 form, but alternative formats are acceptable (e.g., summary letter).

Under requirements of 21 CFR312.23, the completed MedWatch form and FDA Form 1571 must be sent to the FDA. If assistance is needed with completing filing the IND safety report, you may contact the Office of Clinical Research (OCR) Protocol Development Officer/IND Manager.

FDA fax number for IND Safety Reports:
Fax: 1 (800) FDA 0178

All written IND Safety Reports submitted to the FDA by the Investigator must also be faxed to Genentech Drug Safety and submitted to the IRB per IRB reporting requirements:

Fax:
(650) 225-4682 OR (650) 225-4630

And to the Site IRB per institutional policies:

2921 Stockton Blvd
Suite 1400, Room 1429
Sacramento, CA 95817
Phone: (916) 703-9151
Fax: (916) 703-9160

For questions related to safety reporting, please fax to Genentech Drug Safety:

Tel: (888) 835-2555
Fax: (650) 225-4682 or (650) 225-4630

13.7.1 IND Annual Reports

Copies to Genentech:
All IND annual reports submitted to the FDA by the Sponsor-Investigator should be copied to Genentech. Copies of such reports should be faxed to Genentech Drug Safety:

Fax:
(650) 225-4682
OR
(650) 225-4630

13.8 Study Close-Out

Any study report submitted to the FDA by the Sponsor-Investigator should be copied to Genentech. This includes all IND annual reports and the Clinical Study Report (final study report). Additionally, any literature articles that are a result of the study should be sent to Genentech. Copies of such reports should be mailed to the assigned Clinical Operations contact for the study:

Amber Lapp IST Study Manager, US Medical Affairs RPS Strategic Solutions-
A Division of PRA International, on assignment with Genentech Inc.
Phone: 484.533.2019 x 3051
Email: lappa@gene.com
Email: anti-pdl-1-mpd3280a-gsur@gene.com

14.0 ETHICAL CONSIDERATIONS AND ADMINISTRATIVE PROCEDURES

14.1 Ethics and Good Clinical Practice

This study must be carried out in compliance with the protocol and Good Clinical Practice, as described in:

1. ICH Harmonized Tripartite Guidelines for Good Clinical Practice 1996.
2. Directive 91/507/EEC, The Rules Governing Medicinal Products in the European Community.
3. US 21 Code of Federal Regulations dealing with clinical studies (including parts 50 and 56 concerning informed consent and IRB regulations).
4. Declaration of Helsinki, concerning medical research in humans (Recommendations Guiding Physicians in Biomedical Research Involving Human Subjects, Helsinki 1964, amended Tokyo 1975, Venice 1983, Hong Kong 1989, Somerset West 1996).

The investigator agrees, when signing the protocol, to adhere to the instructions and procedures described in it and thereby to adhere to the principles of Good Clinical Practice that it conforms to.

14.2 Institutional Review Board/Independent Ethics Committee

Before implementing this study, the protocol, the proposed informed consent form and other information to subjects, must be reviewed by a properly constituted Institutional Review Board/Independent Ethics Committee (IRB/IEC/REB). A signed and dated statement that the protocol and informed consent have been approved by the IRB/IEC/REB must be given to Genentech before study initiation. The name and occupation of the chairman and the members of the IRB/IEC/REB must be supplied to Genentech. Any amendments to the protocol, other than administrative ones, must be approved by this committee.

All human subjects research funded by the Department of Defense (DoD) requires a second-level review to ensure compliance with the human subjects protection regulations and the DoD and Army's requirements. The Human Research Protection Office (HRPO) located in Fort Detrick, Maryland, will conduct this review on behalf of the USAMRMC. See Appendix 8.

14.3 Study Documentation

Each participating site is responsible for submitting copies of all relevant regulatory documentation to the Coordinating Center. The required documents include, but are not limited to the following: local IRB approvals (i.e., protocol, consent form, amendments, patient brochures and recruitment material, etc.), IRB membership rosters, summary of unanticipated problems or protocol deviations, and documentation of expertise of the investigators. The Coordinating Center will provide each participating site with a comprehensive list of the necessary documents. It is the responsibility of the participating sites to maintain copies of all documentation submitted to the Coordinating Center.

14.4 Informed Consent

The investigator must explain to each subject (or legally authorized representative) the nature of the study, its purpose, the procedures involved, the expected duration, the potential risks and benefits involved and any discomfort it may entail. Each subject must be informed that participation in the study is voluntary and that he/she may withdraw from the study at any time and that withdrawal of consent will not affect his/her subsequent medical treatment or relationship with the treating physician.

This informed consent should be given by means of a standard written statement, written in non-technical language. The subject should read and consider the statement before signing and dating it, and should be given a copy of the signed document. If the subject cannot read or sign the documents, oral presentation may be made or signature given by the subject's legally appointed representative, if witnessed by a person not involved in the study, mentioning that the patient could not read or sign the documents. No patient can enter the study before his/her informed consent has been obtained.

The informed consent form is considered to be part of the protocol, and must be submitted by the investigator with it for IRB/IEC/REB approval.

Fertile men and women of child bearing potential should be informed that taking the study medication may involve unknown risks to the fetus if pregnancy were to occur during the study and agree that in order to participate in the study they must adhere to the contraception requirement for the duration of the study.

Acceptable methods of birth control for men and women are the following:
Highly effective methods

- Male sterilization (vasectomy). For female patients, the vasectomized male partner should be the only partner
- True abstinence, if this is your preferred and usual lifestyle
- Hormonal birth control for male patient's partner

Effective methods

- Placement of intrauterine device or intrauterine system
- Condom with spermicidal foam/gel/film/cream/suppository
- Occlusive cap (diaphragm or cervical/vault caps) with spermicidal foam/gel/film/cream/suppository

Unacceptable methods

- Abstinence at certain times of the cycle only, such as during the days of ovulation, after ovulation (based on symptoms or temperature)
- Pre-ejaculatory withdrawal

If there is any question that the patient will not reliably comply, they should not be entered in the study.

In accordance with UCD OCR policy an original signed and dated participant Informed Consent document will reside in a secured location at participating institutions. Copies of the signed and dated Informed Consent document will be provided to the study participant and a copy will be stored in the patient's electronic medical record at the participating institution.

14.5 Discontinuation of Study Support

Genentech reserves the right to discontinue support for any study under the conditions specified in the clinical trial agreement.

14.6 Amendments to the Protocol

Any change or addition to this protocol requires a written protocol amendment that must be approved by Genentech and the investigator before implementation. Any protocol amendment requires approval by the IRB/IEC/REB. A copy of the written approval of the IRB/IEC/REB, must be sent to Genentech.

14.6.1 Publication of Results

Any formal presentation or publication of data from this trial may be published after review and comment by Genentech and prior to any outside submission. Genentech must receive copies of any intended communication in advance of publication (at least ten working days for presentational materials and abstracts and fifteen working days for manuscripts). These requirements acknowledge Genentech's responsibility to provide peer input regarding the scientific content and conclusions of such publications or presentations. Principal Investigator/Institution shall have the final authority to determine the scope and content of its publications, provided such authority shall be exercised with reasonable regard for the interests of Genentech and, in accord with the trial contract and shall not permit disclosure of Genentech confidential or proprietary information.

14.6.2 Disclosure and Confidentiality

The investigator agrees to keep all information provided by Genentech in strict confidence and to request similar confidentiality from his/her staff and the IRB/IEC/REB. Study documents provided by Genentech (investigators' brochures and other material) will be stored appropriately to ensure their confidentiality. The information provided by Genentech to the investigator may not be disclosed to others without direct written authorization from Genentech, except to the extent necessary to obtain informed consent from patients who wish to participate in the trial.

14.6.3 Declaration of Helsinki

The investigator must conduct the trial in accordance with the principles of the Declaration of Helsinki. Copies of the Declaration of Helsinki and amendments will be provided upon request or can be accessed via the website of the World Medical Association at http://www.wma.net/e/policy/17-c_e.html.

14.7 Protocol Deviations

All protocol deviations will be reported in accordance with UCD IRB Administration and UCD Cancer Center OCR policies or the participating site's IRB policies.

14.8 Quality Assurance

Quality assurance audits of select patients and source documents may be conducted by the Quality Assurance and/or Data Safety Committees at participating institutions or UC Davis Cancer Center as outlined in the UC Davis Cancer Center Data and Safety Monitoring plan. Quality assurance audits may be accomplished in one of two ways: (1) source documents and research records for selected patients are mailed/sent by from participating sites to the Coordinating Center for audit, or (2) selected patient records may be audited on-site at participating sites.

The USAMRMC ORP HRPO conducts site visits as part of its responsibility for compliance oversight. Accurate and complete study records must be maintained and made available to representatives of the DoD as a part of their responsibility to protect human subjects in research.

15.0 OVERSIGHT AND MONITORING

15.1 Data and Safety Monitoring

In addition to the requirements for adverse event reporting as outlined in Section 7.0, this protocol is also subject to the UC Davis Comprehensive Cancer Center's (UCDCCC) Data and Safety Monitoring Plan. The UCDCCC is committed to pursuing high-quality patient-oriented clinical research and has established mechanisms to ensure both scientific rigor and patient safety in the conduct of clinical research studies. The UCDCCC relies on a multi-tiered committee system that reviews and monitors all cancer clinical trials and ensures the safety of its participants, in compliance with institutional and federal requirements on adverse event (AE) reporting, verification of data accuracy, and adherence to protocol eligibility requirements, treatment guidelines, and related matters. The Scientific Review Committee (SRC) assumes overall oversight of cancer studies, with assistance and input from two independent, but interacting, committees: the Quality Assurance Committee and the Data and Safety Monitoring Committee. A multi-level review system strengthens the ability of the UCDCCC to fulfill its mission in conducting high quality clinical cancer research.

As per UCDCCC Office of Clinical Research (OCR) standard operating procedures, the principal investigator (PI) and clinical research coordinator meet at least monthly for ongoing study information, to discuss patient data and adverse events and to determine if dose escalation is warranted, when applicable.

According to the UCDCCC Data and Safety Monitoring Plan, any new serious adverse events related to the drugs being used on this trial are reviewed monthly by the UCDCCC Data and

Safety Monitoring Committee and any applicable changes to the study are recommended to the PI, if necessary.

The UCDCCC SRC determines if a UCDCCC Data and Safety Monitoring Board (DSMB) is required. If required, the Data and Safety Monitoring Committee will appoint a DSMB. The DSMB is responsible for reviewing study accrual logs, adverse event information and dose escalation meeting minutes (where applicable) to ensure subject safety and compliance with protocol defined guidelines.

15.2 Data Safety and Monitoring (USAMRMC ORP Human Research Protections Office (HRPO) Guidelines)

The Research Monitor Dr. Edward Kim is responsible to oversee the safety of the research and report observations/findings to the IRB or a designated institutional official. The Research Monitor will review all unanticipated problems involving risks to subjects or others associated with the protocol and provide an independent report of the event to the IRB. The Research Monitor may discuss the research protocol with the investigators; shall have authority to stop a research protocol in progress, remove individual human subjects from a research protocol, and take whatever steps are necessary to protect the safety and well-being of human subjects until the IRB can assess the monitor's report; and shall have the responsibility to promptly report their observations and findings to the IRB or other designated official and the HRPO.

15.3 Investigator Monitoring Guidelines

Investigators will conduct continuous review of patient safety. As mentioned in section 3.0, patients will be monitored weekly during the 9-week DLT period and every 3 weeks for the remaining three cycles or more frequently if needed. All patients on active treatment will be discussed at weekly teleconferences that are held between the UC (University of California) Davis team and Dr. Mitchell at David Grant USAF (United States Air Force) Medical Center. Per Cancer Center guidelines, a trial cannot proceed to the next dose level until a DLT meeting is conducted to comprehensively review all toxicity data and approve the dose escalation. The discussion will include for each dose level: the number of patients, significant toxicities as described in the protocol, doses adjustments, and responses observed.

All patients will be monitored for a minimum of 12 months following SAR to evaluate for long-term radiation effects. Preliminary efficacy as determined by ORR and DFS will be assessed every 2 cycles then every 3 months for the first two years and every 6 months for the next 3-5 years. Follow-up will be conducted at the institution where the patient was recruited.

Please see Appendix 8 for Reporting Requirements and Responsibilities of the Principal Investigator to the USAMRMC ORP Human Research Protections Office (HRPO).

16.0 REFERENCES

1. Suga, JM, Nguyen, DV, Mohammed, SM, Brown, M, Calhoun, R, Yoneda, K, Gandara, DR, and Lara, PN, Jr., *Racial disparities on the use of invasive and noninvasive staging in patients with non-small cell lung cancer.* J Thorac Oncol, 2010. **5**(11): p. 1772-8.
2. Crawford, RS, 3rd, Wu, J, Park, D, and Barbour, GL, *A study of cancer in the military beneficiary population.* Mil Med, 2007. **172**(10): p. 1084-8.
3. Zullig, LL, Jackson, GL, Dorn, RA, Provenzale, DT, McNeil, R, Thomas, CM, and Kelley, MJ, *Cancer incidence among patients of the U.S. Veterans Affairs Health Care System.* Mil Med, 2012. **177**(6): p. 693-701. http://www.ncbi.nlm.nih.gov/pmc/articles/PMC3531965/
4. Landrum, MB, Keating, NL, Lamont, EB, Bozeman, SR, and McNeil, BJ, *Reasons for underuse of recommended therapies for colorectal and lung cancer in the Veterans Health Administration.* Cancer, 2012. **118**(13): p. 3345-55.
5. Wang, S, Wong, ML, Hamilton, N, Davoren, JB, Jahan, TM, and Walter, LC, *Impact of age and comorbidity on non-small-cell lung cancer treatment in older veterans.* J Clin Oncol, 2012. **30**(13): p. 1447-55. http://www.ncbi.nlm.nih.gov/pmc/articles/PMC3383118/
6. National Cancer Institute. *Surveillance Epidemiology and End Result: SEER Stat Fact Sheets. Cancer: Lung and Bronchus*. 2014 08/19/14]; Available from: http://seer.cancer.gov/statfacts/html/lungb.html.
7. National Lung Screening Trial Research, T, Aberle, DR, Adams, AM, Berg, CD, Black, WC, Clapp, JD, Fagerstrom, RM, Gareen, IF, Gatsonis, C, Marcus, PM, and Sicks, JD, *Reduced lung-cancer mortality with low-dose computed tomographic screening.* N Engl J Med, 2011. **365**(5): p. 395-409.
8. Timmerman, R, Paulus, R, Galvin, J, Michalski, J, Straube, W, Bradley, J, Fakiris, A, Bezjak, A, Videtic, G, Johnstone, D, Fowler, J, Gore, E, and Choy, H, *Stereotactic body radiation therapy for inoperable early stage lung cancer.* JAMA, 2010. **303**(11): p. 1070-6. http://www.ncbi.nlm.nih.gov/pmc/articles/PMC2907644/
9. Baumann, P, Nyman, J, Hoyer, M, Wennberg, B, Gagliardi, G, Lax, I, Drugge, N, Ekberg, L, Friesland, S, Johansson, KA, Lund, JA, Morhed, E, Nilsson, K, Levin, N, Paludan, M, Sederholm, C, Traberg, A, Wittgren, L, and Lewensohn, R, *Outcome in a prospective phase II trial of medically inoperable stage I non-small-cell lung cancer patients treated with stereotactic body radiotherapy.* J Clin Oncol, 2009. **27**(20): p. 3290-6.
10. Fakiris, AJ, McGarry, RC, Yiannoutsos, CT, Papiez, L, Williams, M, Henderson, MA, and Timmerman, R, *Stereotactic body radiation therapy for early-stage non-small-cell lung carcinoma: four-year results of a prospective phase II study.* Int J Radiat Oncol Biol Phys, 2009. **75**(3): p. 677-82.
11. Zheng, X, Schipper, M, Kidwell, K, Lin, J, Reddy, R, Ren, Y, Chang, A, Lv, F, Orringer, M, and Spring Kong, FM, *Survival Outcome After Stereotactic Body Radiation Therapy and Surgery for Stage I Non-Small Cell Lung Cancer: A Meta-Analysis.* Int J Radiat Oncol Biol Phys, 2014.
12. Chang, JY, Liu, H, Balter, P, Komaki, R, Liao, Z, Welsh, J, Mehran, RJ, Roth, JA, and Swisher, SG, *Clinical outcome and predictors of survival and pneumonitis after*

stereotactic ablative radiotherapy for stage I non-small cell lung cancer. Radiat Oncol, 2012. **7**: p. 152. http://www.ncbi.nlm.nih.gov/pmc/articles/PMC3444889/

13. Matsuo, Y, Shibuya, K, Nakamura, M, Narabayashi, M, Sakanaka, K, Ueki, N, Miyagi, K, Norihisa, Y, Mizowaki, T, Nagata, Y, and Hiraoka, M, *Dose-volume metrics associated with radiation pneumonitis after stereotactic body radiation therapy for lung cancer.* Int J Radiat Oncol Biol Phys, 2012. **83**(4): p. e545-9.
14. Baker, R, Han, G, Sarangkasiri, S, DeMarco, M, Turke, C, Stevens, CW, and Dilling, TJ, *Clinical and dosimetric predictors of radiation pneumonitis in a large series of patients treated with stereotactic body radiation therapy to the lung.* Int J Radiat Oncol Biol Phys, 2013. **85**(1): p. 190-5.
15. Barriger, RB, Forquer, JA, Brabham, JG, Andolino, DL, Shapiro, RH, Henderson, MA, Johnstone, PA, and Fakiris, AJ, *A dose-volume analysis of radiation pneumonitis in non-small cell lung cancer patients treated with stereotactic body radiation therapy.* Int J Radiat Oncol Biol Phys, 2012. **82**(1): p. 457-62.
16. Stanic, S, Paulus, R, Timmerman, RD, Michalski, JM, Barriger, RB, Bezjak, A, Videtic, GM, and Bradley, J, *No clinically significant changes in pulmonary function following stereotactic body radiation therapy for early- stage peripheral non-small cell lung cancer: an analysis of RTOG 0236.* Int J Radiat Oncol Biol Phys, 2014. **88**(5): p. 1092-9.
17. Chi, A, Liao, Z, Nguyen, NP, Xu, J, Stea, B, and Komaki, R, *Systemic review of the patterns of failure following stereotactic body radiation therapy in early-stage non-small-cell lung cancer: clinical implications.* Radiother Oncol, 2010. **94**(1): p. 1-11.
18. Allibhai, Z, Taremi, M, Bezjak, A, Brade, A, Hope, AJ, Sun, A, and Cho, BC, *The impact of tumor size on outcomes after stereotactic body radiation therapy for medically inoperable early-stage non-small cell lung cancer.* Int J Radiat Oncol Biol Phys, 2013. **87**(5): p. 1064-70.
19. Clarke, K, Taremi, M, Dahele, M, Freeman, M, Fung, S, Franks, K, Bezjak, A, Brade, A, Cho, J, Hope, A, and Sun, A, *Stereotactic body radiotherapy (SBRT) for non-small cell lung cancer (NSCLC): is FDG-PET a predictor of outcome?* Radiother Oncol, 2012. **104**(1): p. 62-6.
20. Lee, DS, Kim, YS, Yoo Ie, R, Kang, YN, Kim, SJ, Oh, JK, Kim, YK, Wang, YP, Park, JG, Kang, JH, Han, DH, Ahn, MI, and Lee, KY, *Long-term clinical experience of high-dose ablative lung radiotherapy: high pre-treatment [18F]fluorodeoxyglucose-positron emission tomography maximal standardized uptake value of the primary tumor adversely affects treatment outcome.* Lung Cancer, 2013. **80**(2): p. 172-8.
21. Erlich, P, *Ueber den jetzigen stand der karzinomforschung.* Ned Tijdschr Geneeskd 1909. **5**: p. 273-90.
22. Old, LJ and Boyse, EA, *Immunology of experimental tumors.* Annu Rev Med 1964. **15**: p. 167-86.
23. Topalian, SL, Drake, CG, and Pardoll, DM, *Targeting the PD-1/B7-H1(PD-L1) pathway to activate anti-tumor immunity.* Curr Opin Immunol, 2012. **24**(2): p. 207-12. http://www.ncbi.nlm.nih.gov/pmc/articles/PMC3319479/
24. Brahmer, JR, Horn, L, Antonia, SJ, Spigel, DR, Gandhi, L, Sequist, LV, Sankar, V, Ahlers, CM, Wigginton, JM, Kollia, GD, Gupta, A, and Gettinger, SN, *Survival and long-term follow-up of the phase I trial of nivolumab (Anti-PD-1; BMS-936558; ONO-4538) in patients (pts) with previously treated advanced non-small cell lung cancer (NSCLC).* J Clin Oncol, 2013. **31**(suppl; abstr 8030).

25. Brahmer, JR, Horn, L, Antonia, SJ, Spigel, DR, Gandhi, L, Sequist, LV, Sankar, V, Ahlers, CM, Wigginton, JM, Kollia, GD, Gupta, A, and Gettinger, SN, *Nivolumab (anti-PD-1; BMS-936558; ONO-4538) in patients with non-small cell lung cancer (NSCLC): Overall survival and long-term safety in a phase 1 trial.* J Thoracic Oncol, 2013. **8**(suppl 2 (abstr MO18.03)).
26. Garon, EB, Leigh, NB, Rizvi, NA, Blumenschein, GR, Balmanoukian, AS, Eder, JP, Goldman, JW, Hui, R, Soria, J-C, Gangadhar, TC, Sun, J-M, Patnaik, A, Gubens, MA, Lubiniecki, GM, Zhang, J, Niewood, M, Emancipator, K, Dolled-Filhart, M, Hanson, ME, and Gandhi, L, *Safety and clinical activity of MK-3475 in previously treated patients (pts) with non-small cell lung cancer (NSCLC).* J Clin Oncol, 2014. **32**(5s): p. (abstr 8020).
27. Horn, L, Herbst, RS, Spigel, DR, Gettinger, SN, Gordon, MS, Hollebecque, A, Gandhi, L, Felip, E, Heist, R, Mokatrin, A, Kowanetz, M, Waterkamp, D, Fine, G, and Soria, J-C, *An analysis of the relationship of clinical activity to baseline EGFR status, PDL1 expression and prior treatment history in patients with non-small cell lung cancer (NSCLC) following PD-L1 blockade with MPDL3280A (anti-PDL1).* J Thorac Oncol, 2013. **8**(suppl 2 (abstr MO18.01)).
28. Topalian, SL, Hodi, FS, Brahmer, JR, Gettinger, SN, Smith, DC, McDermott, DF, Powderly, JD, Carvajal, RD, Sosman, JA, Atkins, MB, Leming, PD, Spigel, DR, Antonia, SJ, Horn, L, Drake, CG, Pardoll, DM, Chen, L, Sharfman, WH, Anders, RA, Taube, JM, McMiller, TL, Xu, H, Korman, AJ, Jure-Kunkel, M, Agrawal, S, McDonald, D, Kollia, GD, Gupta, A, Wigginton, JM, and Sznol, M, *Safety, activity, and immune correlates of anti-PD-1 antibody in cancer.* N Engl J Med, 2012. **366**(26): p. 2443-54. http://www.ncbi.nlm.nih.gov/pmc/articles/PMC3544539/
29. Rizvi, NA, Garon, EB, Patnaik, A, Gandhi, L, Leighl, NB, Balmanoukian, AS, Goldman, JW, Eder, JP, Johnson, E, Blumenschein, GR, Gubens, MA, Papadopoulos, KP, Lubiniecki, GM, Zhang, J, Niewood, M, Emancipator, K, Dolled-Filhart, M, Hanson, ME, and Hui, R, *Safety and clinical activity of MK-3475 as intial therapy in patients with advanced non-small cell lung cancer (NSCLC).* J Clin Oncol, 2014. **32**(5a): p. (abstr 8007).
30. Gettinger, SN, Shepherd, FA, Antonia, SJ, Brahmer, JR, Chow, LQM, Juergens, RA, Borghaei, H, Shen, Y, Harbison, C, Alaparthy, S, Chen, AC, and Rizvi, NA, *First-line nivolumab (anti-PD-1; BMS-936558; ONO-4538) monotherapy in advanced NSCLC: Safety, efficacy, and correlation of outcomes with PD-L1 status.* J Clin Oncol, 2014. **32**(5s): p. (abstr 8024).
31. Antonia, SJ, Brahmer, JR, Gettinger, SN, Chow, LQM, Juergens, RA, Shepherd, FA, Laurie, SA, Gerber, DE, Goldman, JW, Shen, Y, Harbison, C, Alaparthy, S, and Chen, AC, *Nivolumab (anti-PD-1; BMS-936558; ONO-4538) in combination with platinum-based doublet chemotherpy (PT-DC) in advanced non-small cell lung cancer (NSCLC).* J Clin Oncol, 2014. **32**(5s): p. (abstr 8113).
32. Rizvi, NA, Chow, LQM, Borghaei, H, Shen, Y, Harbison, C, Alaparthy, S, Chen, AC, and Gettinger, SN, *Safety and response with nivolumab (anti-PD-1; BMS-936558l; ONO-4538) plus erlotinib in pateints (pts) with epidermal growth factor receptor mutat (EGFR MT) advanced NSCLC.* J Clin Oncol, 2014. **32**(5s): p. (abstr 8022).
33. Herbst, RS, Gordon, MS, Fine, GD, Sosman, JA, Soria, J-C, Hamid, O, Powderly, JD, Burris, HA, Mokatrin, A, Kowanetz, M, Leabman, M, Anderson, M, Chen, DS, and

Hodi, FS, *A study of MPDL3280A, an engineered PD-L1 antibody in patients with locally advanced or metastatic tumors.* J Clin Oncol, 2013. **31**: p. (suppl; abstr 3000).

34. Spigel, DR, Gettinger, SN, Horn, L, Herbst, RS, Gandhi, L, Gordon, MS, Cruz, C, Conkling, P, Cassier, PA, Antonia, SJ, Burris, HA, Fine, GD, Mokatrin, A, Kowanetz, M, Shen, X, Chen, DS, Soria, J-C, and Cannon, S, *Clinical activity, safety and biomarkers of MPDL3280A, an engineered PD-L1 antibody in patients with locally advanced or metastatic non-small cell lung cancer (NSCLC).* J Clin Oncol, 2013. **31**: p. (suppl; abstr 8008).
35. Formenti, SC and Demaria, S, *Systemic effects of local radiotherapy.* Lancet Oncol, 2009. **10**(7): p. 718-26. http://www.ncbi.nlm.nih.gov/pmc/articles/PMC2782943/
36. Formenti, SC and Demaria, S, *Combining radiotherapy and cancer immunotherapy: a paradigm shift.* J Natl Cancer Inst, 2013. **105**(4): p. 256-65. http://www.ncbi.nlm.nih.gov/pmc/articles/PMC3576324/
37. Klug, F, Prakash, H, Huber, PE, Seibel, T, Bender, N, Halama, N, Pfirschke, C, Voss, RH, Timke, C, Umansky, L, Klapproth, K, Schakel, K, Garbi, N, Jager, D, Weitz, J, Schmitz-Winnenthal, H, Hammerling, GJ, and Beckhove, P, *Low-dose irradiation programs macrophage differentiation to an iNOS(+)/M1 phenotype that orchestrates effective T cell immunotherapy.* Cancer Cell, 2013. **24**(5): p. 589-602.
38. Wu, CY, Yang, LH, Yang, HY, Knoff, J, Peng, S, Lin, YH, Wang, C, Alvarez, RD, Pai, SI, Roden, RB, Hung, CF, and Wu, TC, *Enhanced cancer radiotherapy through immunosuppressive stromal cell destruction in tumors.* Clin Cancer Res, 2014. **20**(3): p. 644-57.
39. Apetoh, L, Ghiringhelli, F, Tesniere, A, Obeid, M, Ortiz, C, Criollo, A, Mignot, G, Maiuri, MC, Ullrich, E, Saulnier, P, Yang, H, Amigorena, S, Ryffel, B, Barrat, FJ, Saftig, P, Levi, F, Lidereau, R, Nogues, C, Mira, JP, Chompret, A, Joulin, V, Clavel-Chapelon, F, Bourhis, J, Andre, F, Delaloge, S, Tursz, T, Kroemer, G, and Zitvogel, L, *Toll-like receptor 4-dependent contribution of the immune system to anticancer chemotherapy and radiotherapy.* Nat Med, 2007. **13**(9): p. 1050-9.
40. Obeid, M, Tesniere, A, Ghiringhelli, F, Fimia, GM, Apetoh, L, Perfettini, JL, Castedo, M, Mignot, G, Panaretakis, T, Casares, N, Metivier, D, Larochette, N, van Endert, P, Ciccosanti, F, Piacentini, M, Zitvogel, L, and Kroemer, G, *Calreticulin exposure dictates the immunogenicity of cancer cell death.* Nat Med, 2007. **13**(1): p. 54-61.
41. Demaria, S, Kawashima, N, Yang, AM, Devitt, ML, Babb, JS, Allison, JP, and Formenti, SC, *Immune-mediated inhibition of metastases after treatment with local radiation and CTLA-4 blockade in a mouse model of breast cancer.* Clin Cancer Res, 2005. **11**(2 Pt 1): p. 728-34.
42. Deng, L, Liang, H, Burnette, B, Beckett, M, Darga, T, Weichselbaum, RR, and Fu, YX, *Irradiation and anti-PD-L1 treatment synergistically promote antitumor immunity in mice.* J Clin Invest, 2014. **124**(2): p. 687-95. http://www.ncbi.nlm.nih.gov/pmc/articles/PMC3904601/
43. Seung, SK, Curti, BD, Crittenden, M, Walker, E, Coffey, T, Siebert, JC, Miller, W, Payne, R, Glenn, L, Bageac, A, and Urba, WJ, *Phase 1 study of stereotactic body radiotherapy and interleukin-2--tumor and immunological responses.* Sci Transl Med, 2012. **4**(137): p. 137ra74.
44. Slovin, SF, Higano, CS, Hamid, O, Tejwani, S, Harzstark, A, Alumkal, JJ, Scher, HI, Chin, K, Gagnier, P, McHenry, MB, and Beer, TM, *Ipilimumab alone or in combination*

with radiotherapy in metastatic castration-resistant prostate cancer: results from an open-label, multicenter phase I/II study. Ann Oncol, 2013. **24**(7): p. 1813-21. http://www.ncbi.nlm.nih.gov/pmc/articles/PMC3707423/

45. Postow, MA, Callahan, MK, Barker, CA, Yamada, Y, Yuan, J, Kitano, S, Mu, Z, Rasalan, T, Adamow, M, Ritter, E, Sedrak, C, Jungbluth, AA, Chua, R, Yang, AS, Roman, RA, Rosner, S, Benson, B, Allison, JP, Lesokhin, AM, Gnjatic, S, and Wolchok, JD, *Immunologic correlates of the abscopal effect in a patient with melanoma.* N Engl J Med, 2012. **366**(10): p. 925-31. http://www.ncbi.nlm.nih.gov/pmc/articles/PMC3345206/
46. Golden, EB, Demaria, S, Schiff, PB, Chachoua, A, and Formenti, SC, *An abscopal response to radiation and ipilimumab in a patient with metastatic non-small cell lung cancer.* Cancer Immunol Res, 2013. **1**(6): p. 365-72.
47. Brody, JD, Ai, WZ, Czerwinski, DK, Torchia, JA, Levy, M, Advani, RH, Kim, YH, Hoppe, RT, Knox, SJ, Shin, LK, Wapnir, I, Tibshirani, RJ, and Levy, R, *In situ vaccination with a TLR9 agonist induces systemic lymphoma regression: a phase I/II study.* J Clin Oncol, 2010. **28**(28): p. 4324-32. http://www.ncbi.nlm.nih.gov/pmc/articles/PMC2954133/
48. Kim, YH, Gratzinger, D, Harrison, C, Brody, JD, Czerwinski, DK, Ai, WZ, Morales, A, Abdulla, F, Xing, L, Navi, D, Tibshirani, RJ, Advani, RH, Lingala, B, Shah, S, Hoppe, RT, and Levy, R, *In situ vaccination against mycosis fungoides by intratumoral injection of a TLR9 agonist combined with radiation: a phase 1/2 study.* Blood, 2012. **119**(2): p. 355-63. http://www.ncbi.nlm.nih.gov/pmc/articles/PMC3257006/
49. Kandoth, C, McLellan, MD, Vandin, F, Ye, K, Niu, B, Lu, C, Xie, M, Zhang, Q, McMichael, JF, Wyczalkowski, MA, Leiserson, MD, Miller, CA, Welch, JS, Walter, MJ, Wendl, MC, Ley, TJ, Wilson, RK, Raphael, BJ, and Ding, L, *Mutational landscape and significance across 12 major cancer types.* Nature, 2013. **502**(7471): p. 333-9. http://www.ncbi.nlm.nih.gov/pmc/articles/PMC3927368/
50. Aljubran, A, Leighl, N, Pintilie, M, Feld, R, Burkes, R, and Shepherd, F, *Compliance of adjusted chemotherapy in non-small cell lung cancer (NSCLC); No longer an issue.* J Clin Oncol, 2006. **24**(18s): p. (abstr 7154).
51. McDermott AJ, Huffnagle GB. The microbiome and regulation of mucosal immunity. Immunology 2014;142:24-31.
52. Viaud S, Daillere R, Boneca IG, et al. Harnessing the intestinal microbiome for optimal therapeutic immunomodulation. Cancer research 2014;74:4217-21.
53. Eisenhauer, EA, Therasse, P, Bogaerts, J, Schwartz, LH, Sargent, D, Ford, R, Dancey, J, Arbuck, S, Gwyther, S, Mooney, M, Rubinstein, L, Shankar, L, Dodd, L, Kaplan, R, Lacombe, D, and Verweij, J, *New response evaluation criteria in solid tumours: revised RECIST guideline (version 1.1).* Eur J Cancer, 2009. **45**(2): p. 228-47.
54. Berriochoa C, Videtic GM, Woody NM, Djemil T, Zhuang T, Stephans KL. *Stereotactic Body Radiotherapy for T3N0 Lung Cancer With Chest Wall Invasion*. Clin Lung Cancer, 2016. **17**(6): p.595-601.

17.0 APPENDICES

Appendix 1: Performance Status Scale

ECOG PERFORMANCE STATUS SCALE

ECOG (Zubrod)	Karnofsky	Definitions
0	100	Asymptomatic
1	80-90	Symptomatic, fully ambulatory
2	60-70	Symptomatic, in bed less than 50% of the day
3	40-50	Symptomatic, in bed more than 50% of the day, but not bedridden
4	20-30	Bedridden

Appendix 2: Study Registration

A. Registrations for this protocol must be made through the Office of Clinical Research (OCR) of the University of California, Davis Cancer Center between normal business hours (Pacific Time), Monday through Friday (except holidays). Documentation of current IRB approval of this protocol by non-UCD institutions must be on file prior to registration of patients at these institutions.

B. Pre-study laboratory tests, scans, and x-rays, must be completed prior to registration, within the time frame specified in the protocol. The eligibility checklist must be completed. Patients must sign an informed consent prior to registration.

C. If the patient is to be registered the same day as the proposed treatment start date, the UC Davis Study Coordinator must be notified by fax (916-734-4177) and/or email 24 hrs prior to proposed treatment start date that the site has a patient to register.

D. Patients may be registered up to 72 hrs prior to treatment initiation. The signed consent, completed checklist and reports from all pre-study laboratory tests, scans and x-rays must be faxed/email to the University of California, Davis Comprehensive Cancer Center Clinical Trials Support Unit in order to register the patient. The UC Davis Study Coordinator will review these documents and fax a registration confirmation within 24 hours. **NOTE: Administration of study medication may not be initiated until the registration confirmation has been received.**

E. A patient failing to meet all protocol requirements may not be registered. If you have any questions regarding eligibility, please contact the coordinating site PI or Study Coordinator

Appendix 3: Data Submission Schedule

All data will be collected using UC Davis data collection forms. Copies of the completed forms will be submitted to UC Davis data coordinating center for data entry and storage in a secure location. The original data collection forms will reside at the originating institution in secure location.

- SUBMIT WITHIN 24 HOURS OF REGISTRATION:
  Patient Registration Form

- SUBMIT WITHIN 14 DAYS OF REGISTRATION:
  In-House Pre-Study Evaluation Form (IH-102)

- SUBMIT WITHIN 7 DAYS OF SCREENING FAILURE:
  Patient Screen Failure Form

- SUBMIT WITH 14 DAYS OF CYCLE COMPLETION:
  Adverse Event/Drug Relationship Form

- SUBMIT WITHIN 14 DAYS OF END OF EACH TREATMENT CYCLE:
  In-House Treatment Cycle Form – Infusion

- SUBMIT WITHIN 14 DAYS OF EACH RESPONSE ASSESSMENT:
  Tumor Measurement Log

- SUBMIT WITHIN 14 DAYS OF OFF TREATMENT:
  Off Treatment/In Follow-up/Off Study/Expiration Form (IH-301)

- SUBMIT WITHIN 14 DAYS OF KNOWLEDGE OF DEATH IF PATIENT IS STILL ON STUDY OR 30–DAYS IF OFF STUDY:
  Off Treatment/In Follow-up/Off Study/Expiration Form (IH-301)

- SUBMIT WITHIN 2 DAYS OF KNOWLEDGE OF PROTOCOL DEVIATION:
  Clinical Trials Support Unit: Notice of Protocol Deviation

- SUBMIT WITHIN 14 DAYS OF EACH REQUIRED FOLLOW-UP ENCOUNTER:
  Follow-Up Form (IH-302)

- ALL SERIOUS ADVERSE EVENTS MUST BE REPORTED AS OUTLINED IN THE PROTOCOL.

Appendix 4: Molecular Correlative Sample Handling

Specimen Submission for Correlative Studies:
Patients must be offered participation in these molecular correlative studies. With the patient's consent, tissue and blood specimens will be submitted as outlined below. Samples will be de-identified and coded with a new patient ID number to protect patient's identity.

Specimen Collection, Storage, Shipping and Submission Requirements
It is required that paraffin-embedded tissue blocks or slides from time of diagnosis (or subsequent, but prior to therapy) as well as blood specimens, as outlined below, be submitted for expression of relevant molecular targets.

• **Archival tumor specimens**: If available, 1 - 2 paraffin-embedded tissue blocks containing formalin-fixed tumor or needle aspirate slides from time of diagnosis (or subsequent, but prior to therapy) should be submitted for evaluation of expression of PD-L1 and other relevant molecules. Paraffin blocks may be processed according to standard institutional protocols. If blocks are unavailable, 16 unstained slides are acceptable alternatives.

• Fresh **tumor biopsy**: If deemed appropriate by the study team and with the patient's consent, fresh tumor may be collected after 6 weeks of SAR. **All specimens must be labeled with protocol number, patient registration number, and date of specimen collection.** Tissue portions will be used for (in order of priority):

1. Fixed in formalin for the preparation of FFPE blocks
2. Placed in 1 ml RNA later and snap frozen with liquid nitrogen and stored at -70 to -80°C for future RNA extraction and qPCR and immunochip analysis,
3. Placed in 30-40 ml cold RPMI media and delivered to the lab Laboratory of Cancer Immunology in the Institute for Regenerative Cures for immediate processing, staining, and FACS analysis
4. Snap frozen with liquid nitrogen and stored at -70 to -80°C for future luminex cytokine evaluation

• **Blood specimens**: Blood specimens (3 x 10 ml lavender top EDTA tubes) will be collected from each patient.

Each purple-top (EDTA) tube should be delivered to the lab where it will be inverted several times, and placed on wet ice until centrifugation. The tubes should be centrifuged as soon as possible at approximately 600 x g for 10 minutes. Approximately 1.5 ml of plasma should be removed from each tube and pooled in a fresh, sterile 15 ml conical tube. Centrifuge plasma a second time at 1500-1600 x g for 5 – 10 minutes to pellet and remaining cells. After the second spin, plasma should be removed and placed in 500 ul aliquots in labeled cryotubes. Plasma will be frozen and stored at -70 to -80°C. Plasma samples will be assayed by Luminex to evaluate systemic cytokine levels.

Blood remaining in the original EDTA tubes will be processed for PBMC collection. Briefly, blood cells will be resuspended in room temperature PBS at a 2:1 ratio. The blood will then be carefully layered over Lymphocyte Separation Media (Ficol Solution, LSM) and centrifuged at

400 x g for 30 minutes. Resulting cells will be washed twice in PBS then resuspended in RPMI to facilitate cell counting. To retain viability of cells, cells will be frozen at about 1.0 x 107 in a freezing media consisting of 50% RPMI, 40% FBS, & 10% DMSO. Cells will be frozen at -1°C/minute, the optimal rate for cell preservation.

- **Stool microbiome analysis** will be performed on stool samples as a fee for service by MicroTrek. Patients will receive a stool sample kit. The specimen will be placed at -20 if collected at home and then placed in a -80 freezer. Samples collected on site will be immediately placed in a -80 freezer.

- **Shipping Instructions:** All archival paraffin block or slide specimens should be sent at ambient temperature with a room temperature cool pack. Frozen specimens (blood and stool samples) should be shipped on **dry ice**. Batch shipping is recommended. These should be shipped by overnight courier Monday through Wednesday only, to the following address:

Anthony Martinez, BS
UC Davis Comprehensive Cancer Center
4501 X Street, Suite 1009
Sacramento, CA 95817
Phone: 916-734-6447
Email: axmartinez@ucdavis.edu

A Specimen Submission Form must be submitted with each specimen. Institutions should notify the recipient by either phone or fax prior to shipping specimens. This will allow the recipient to track the package in the event that there are any problems in delivery.

The Federal Guidelines for Shipment are as follows (these periodically change, please check for the most current guidelines):
1. The specimen must be wrapped in an absorbable material
2. The specimen must then be placed in an AIRTIGHT container (resealable bag)
3. Pack the resealable bag and specimen in a styrofoam shipping container
4. Pack the styrofoam shipping container in a cardboard box
5. The cardboard box should be labeled “UN3373 Biological Substance, Category B” “BIOHAZARD”

Appendix 5: Anaphylaxis Precautions

EQUIPMENT NEEDED

Tourniquet
Oxygen
Epinephrine for subcutaneous, intravenous, and/or endotracheal use in accordance with standard practice
Antihistamines
Corticosteroids
Intravenous infusion solutions, tubing, catheters, and tape

PROCEDURES

In the event of a suspected anaphylactic reaction during study drug infusion, the following procedures should be performed:

1. Stop the study drug infusion.
2. Apply a tourniquet proximal to the injection site to slow systemic absorption of study drug. Do not obstruct arterial flow in the limb.
3. Maintain an adequate airway.
4. Administer antihistamines, epinephrine, or other medications as required by patient status and directed by the physician in charge.
5. Continue to observe the patient and document observations.

Appendix 6: Immune-Related Response Criteria (irRC)

Increasing clinical experience indicates that traditional response criteria (e.g., Response Evaluation Criteria in Solid Tumors, Version 1.1 [RECIST v1.1] and World Health Organization [WHO]) may not be sufficient to characterize fully activity in the new era of target therapies and/or biologics. In studies with cytokines, cancer vaccines, and monoclonal antibodies, complete response, partial response, or stable disease has been shown to occur after an increase in tumor burden as characterized by progressive disease by traditional response criteria. Therefore, conventional response criteria may not adequately assess the activity of immunotherapeutic agents because progressive disease (by initial radiographic evaluation) does not necessarily reflect therapeutic failure. Long-term effect on the target disease must also be captured. The immune-related response criteria[1] (irRC) are criteria that attempt to do that by enhancing characterization of new response patterns that have been observed with immunotherapeutic agents (i.e., ipilimumab). (Note: The irRC only index and measurable new lesions are taken into account.)

GLOSSARY

Term	Definition
SPD	sum of the products of the two largest perpendicular diameters
Tumor burden	$SPD_{\text{index lesions}} + SPD_{\text{new, measurable lesions}}$
Nadir	minimally recorded tumor burden
irCR	immune-related complete response
irPD	immune-related progressive disease
irPR	immune-related partial response
irSD	immune-related stable disease
irBOR	immune-related best overall response

BASELINE ASSESSMENT USING irRC

Step 1. Identify the index lesions (five lesions per organ, up to ten visceral lesions and five cutaneous lesions).

Step 2. Calculate the SPD of all of these index lesions:

$$SPD = \sum_i (\text{Largest diameter of lesion i}) \times (\text{Second largest diameter of lesion i}).$$

[1] Wolchok JD, Hoos A, O'Day S, et al. Guidelines for the evaluation of immune therapy activity in solid tumors: immune-related response criteria. Clin Can Res 2009;15:7412–20.

POST-BASELINE ASSESSMENTS USING irRC

Step 1. Calculate the SPD of the index lesions.

Step 2. Identify new, measurable lesions ($\geq 5 \times 5$ mm; up to five new lesions per organ: five new cutaneous lesions and ten visceral lesions).

Step 3. Calculate the SPD of the new, measurable lesions.

Step 4. Calculate the tumor burden:
Tumor burden = $SPD_{\text{index lesions}} + SPD_{\text{new, measurable lesions}}$

Step 5. Calculate the change in tumor burden relative to baseline and the change in tumor burden relative to nadir.

Step 6. Derive the overall response using the table below.

Overall Response	Criterion
irCR	Complete disappearance of all lesions (whether measurable or not, and no new lesions) confirmed by a repeat, consecutive assessment ≥ 4 weeks from the date first documented
irPR	Decrease in tumor burden ≥ 50% relative to baseline confirmed by a consecutive assessment ≥ 4 weeks from the date first documented
irSD	Criteria for irCR, irPR, and irPD are not met; does not require confirmation
irPD	Increase in tumor burden ≥ 25% relative to nadir confirmed by a consecutive assessment ≥ 4 weeks from the date first documented

irCR = immune-related complete response; irPD = immune-related progressive disease; irPR = immune-related partial response; irSD = immune-related stable disease.

DETERMINATION OF irBOR

Once a patient has completed all tumor assessments, his/her irBOR may be determined:

Condition	irBOR
At least one irCR	irCR
At least one irPR and no irCR	irPR
At least one irSD and no irCR and no irPR	irSD
At least one irPD and no irCR, no irPR, and no irSD	irPD

irBOR = immune-related best overall response; irCR = immune-related complete response; irPD = immune-related progressive disease; irPR = immune-related partial response; irSD = immune-related stable disease.

Appendix 7: Safety Reporting Fax Cover Sheet

Genentech
A Member of the Roche Group

GENENTECH SUPPORTED RESEARCH

- AE/SAE FAX No: (650) 225-4682
- Alternate Fax No: (650) 225-4630

Page 1 of ___

Genentech Study Number	ML29955
Principal Investigator	
Site Name	
Reporter name	
Reporter Telephone #	
Reporter Fax #	
Initial Report Date	____________/_____/_____<br>dd / mmm / yyyy
Follow-up Report Date	____________/_____/_____<br>dd / mmm / yyyy
Patient ID Number	____________

SAE or Safety Reporting questions, contact Genentech Safety: (888) 835-2555
PLEASE PLACE MEDWATCH REPORT or SAFETY REPORT BEHIND THIS COVER SHEET.

Appendix 8: Reporting Requirements and Responsibilities of the Principal Investigator to the USAMRMC ORP Human Research Protections Office (HRPO)

The Principal Investigator must comply with the following minimum reporting requirements. Specific reporting requirements for the protocol will be included in the HRPO Approval Memorandum. Failure to comply could result in suspension of funding.

The protocol will not be initiated until written notification of approval of the research project is issued by the USAMRMC ORP HRPO.

1. Substantive modifications to the research protocol and any modifications that could potentially increase risk to subjects must be submitted to the HRPO for approval prior to implementation. The USAMRMC ORP HRPO defines a substantive modification as a change in Principal Investigator, change or addition of an institution, elimination or alteration of the consent process, change to the study population that has regulatory implications (e.g. adding children, adding active duty population, etc), significant change in study design (i.e. would prompt additional scientific review) or a change that could potentially increase risks to subjects.

2. Any changes of the IRB used to review and approve the research will be promptly reported to the USAMRMC ORP HRPO.

3. All unanticipated problems involving risk to subjects or others must be promptly reported by telephone (301-619-2165), by email (usarmy.detrick.medcom usamrmc.other.hrpo@mail.mil), or by facsimile (301-619-7803) to the HRPO. A complete written report will follow the initial notification. In addition to the methods above, the complete report can be sent to the US Army Medical Research and Materiel Command, ATTN: MCMR-RP, 810 Schreider Street, Fort Detrick, Maryland 21702-5000.

4. Suspensions, clinical holds (voluntary or involuntary), or terminations of this research by the IRB, the institution, the Sponsor, or regulatory agencies will be promptly reported to the USAMRMC ORP HRPO.

5. A change in subject status when a previously enrolled human subject becomes a prisoner must be promptly reported to USAMRMC ORP HRPO. The report must include actions taken by the institution and the Institutional Review Board.

6. A copy of the continuing review approval notification by the IRB of Record must be submitted to the HRPO as soon as possible after receipt. Please note that the HRPO also conducts random audits at the time of continuing review. Additional information and documentation may be requested at that time.

7. The final study report, including any acknowledgement documentation and supporting documents, must be submitted to the HRPO when available.

8. The knowledge of any pending compliance inspection/visit by the FDA, DHHS Office of Human Research Protections (OHRP), or other government agency concerning this research, the issuance of Inspection Reports, FDA Form 483, warning letters or actions taken by any regulatory agencies including legal or medical actions and any instances of serious or continuing noncompliance with the regulations or requirements, must be promptly reported to the HRPO.

Please Note: The USAMRMC ORP HRPO conducts site visits as part of its responsibility for compliance oversight. Accurate and complete study records must be maintained and made available to representatives of the DoD as a part of their responsibility to protect human subjects in research. Research records must be stored in a confidential manner so as to protect the confidentiality of subject information.

For questions regarding the HRPO human research protocol review requirements email usarmy.detrick.medcom-usamrmc.other.hrpo@mail.mil or leave a voicemail at 301-619-2165.
