# Supplementary material for: Atezolizumab plus stereotactic ablative radiotherapy for medically inoperable patients with early-stage non-small cell lung cancer: a multi-institutional phase I trial
Source: Nat Commun. 2023 Sep 2;14:5332. doi: 10.1038/s41467-023-40813-w (PMC10474145; doi:10.1038/s41467-023-40813-w)
Supplement: Supplementary file 3 — Reporting Summary [file 41467_2023_40813_MOESM3_ESM.pdf]

nature portfolio

Corresponding author(s): Megan E. Daly

Last updated by author(s): Jul 26, 2023

Reporting Summary

Nature Portfolio wishes to improve the reproducibility of the work that we publish. This form provides structure for consistency and transparency in reporting. For further information on Nature Portfolio policies, see our Editorial Policies and the Editorial Policy Checklist.

Statistics

For all statistical analyses, confirm that the following items are present in the figure legend, table legend, main text, or Methods section.

n/a	Confirmed	
[ ]	[x]	The exact sample size (*n*) for each experimental group/condition, given as a discrete number and unit of measurement
[ ]	[x]	A statement on whether measurements were taken from distinct samples or whether the same sample was measured repeatedly
[ ]	[x]	The statistical test(s) used AND whether they are one- or two-sided<br>*Only common tests should be described solely by name; describe more complex techniques in the Methods section.*
[x]	[ ]	A description of all covariates tested
[ ]	[x]	A description of any assumptions or corrections, such as tests of normality and adjustment for multiple comparisons
[ ]	[x]	A full description of the statistical parameters including central tendency (e.g. means) or other basic estimates (e.g. regression coefficient) AND variation (e.g. standard deviation) or associated estimates of uncertainty (e.g. confidence intervals)
[ ]	[x]	For null hypothesis testing, the test statistic (e.g. *F*, *t*, *r*) with confidence intervals, effect sizes, degrees of freedom and *P* value noted<br>*Give P values as exact values whenever suitable.*
[x]	[ ]	For Bayesian analysis, information on the choice of priors and Markov chain Monte Carlo settings
[x]	[ ]	For hierarchical and complex designs, identification of the appropriate level for tests and full reporting of outcomes
[x]	[ ]	Estimates of effect sizes (e.g. Cohen's *d*, Pearson's *r*), indicating how they were calculated

Our web collection on statistics for biologists contains articles on many of the points above.

Software and code

Policy information about availability of computer code

Data collection	Velos, Microsoft excel
Data analysis	FlowJo software version 6.10.2, Graph pad prism v8.3, STAR v.2.5.1, DESeq2 R package, R statistical package (v.4.1.1), R package logistf and "ROCR" package

For manuscripts utilizing custom algorithms or software that are central to the research but not yet described in published literature, software must be made available to editors and reviewers. We strongly encourage code deposition in a community repository (e.g. GitHub). See the Nature Portfolio guidelines for submitting code & software for further information.

Data

Policy information about availability of data

All manuscripts must include a data availability statement. This statement should provide the following information, where applicable:

- Accession codes, unique identifiers, or web links for publicly available datasets
- A description of any restrictions on data availability
- For clinical datasets or third party data, please ensure that the statement adheres to our policy

The RNA sequencing data generated in this study have been deposited in the sequence read archive (SRA) database under accession code PRJNA999721 [https://www.ncbi.nlm.nih.gov/bioproject/999721]. Individual de-identified participant data on demographics, toxicity, and clinical outcomes, will be maintained by the UC Davis Cancer Center Office of Clinical Research and will be shared for academic purposes on request (Dr. Megan E. Daly, medaly@ucdavis.edu) for at least two years from the date of publication, with the completion of a data access agreement. Individual de-identified data for correlative studies and transcriptomics is included in

the manuscript or publicly available as listed above. Multiplex IHC imaging data is maintained by Dr. Kurt Schalper (kurt.schalper@yale.edu) and will be shared for academic purposes on request for at least 2 years from the date of publication. The study protocol is available as Supplementary Note in the Supplementary Information file.
Source data are provided with this paper. The remaining data are available within the Article, Supplementary Information or Source Data file.

Human research participants

Policy information about studies involving human research participants and Sex and Gender in Research.

Reporting on sex and gender	We did not collect data on sex. We collected self reported sex (gender) characteristics. These are reported in the manuscript. 9 patients identified as male and 11 as female. No analysis was planned or performed based on sex or gender given the small number of patients in this early phase clinical trial.
Population characteristics	Information on age, gender, performance status, smoking status, stage, and high risk features is reported in table 1. Data on race was collected but not included in Table 1. 15 patients were white, 3 were African American, 1 was Asian, and 1 was Native American.
Recruitment	Please see the protocol recruitment plan below:<br>UCDCCC: Patients will be identified at the UC Davis multidisciplinary lung cancer tumor board where new cases are presented and in pulmonary, thoracic surgery and radiation oncology across the UC Davis Health System. Broad recruitment efforts at UC Davis include 1) discussion of the trial at the bimonthly clinical investigators meeting upon trial activation, 2) weekly email broadcasts of phase I trial slot availability; and 3) monthly cancer clinical trials updates to all oncologists, radiation oncologists and surgeons in the northern and central California region.<br>Mercy: Patients will be identified by the radiation oncologists at Mercy Cancer Center or Mercy San Juan Medical Center and referred to UCDCCC for consultation about the trial. Trial flyers and monthly email reminders are sent to these physicians.<br>Military patients and families: Identification of veterans for trial eligibility will be conducted by UC Davis faculty that staff VA Mather and VA Martinez and by radiation oncologists at David Grant USAF Medical Center. David Grant USAF Medical Center physicians may also recruit other military service members or their families. Monthly email reminders are sent to these physicians.<br>Cedars-Sinai Medical Center: Patients will be identified through their multidisciplinary lung cancer tumor board and email reminders. PIs will participate in the monthly trial updates meeting.
Ethics oversight	The study was approved by the IRB at UC Davis, Cedars Sinai, and VA David Grant Medical Center

Note that full information on the approval of the study protocol must also be provided in the manuscript.

Field-specific reporting

Please select the one below that is the best fit for your research. If you are not sure, read the appropriate sections before making your selection.

☒ Life sciences ☐ Behavioural & social sciences ☐ Ecological, evolutionary & environmental sciences

For a reference copy of the document with all sections, see nature.com/documents/nr-reporting-summary-flat.pdf

Life sciences study design

All studies must disclose on these points even when the disclosure is negative.

Sample size	Sample size was determined by the trial design using a traditional 3 + 3 phase I dose escalation design with three planned dose levels and 3-6 patients at each dose level. An expansion cohort of 15 patients was planned but this was closed early due to opening of the Phase III trial.
Data exclusions	No data was excluded, some patients were not evaluable and did not have all data available as described in the manuscript.
Replication	For the clinical trial data no repeated measures were used. For correlative studies repeated measures were used as indicated (multiple fields were counted for PD-L1 histology and multi-plex IF).
Randomization	This is a single arm trial and there was no randomization.
Blinding	This is a single arm trial and there was no randomization or assigned groups for blinding.

Reporting for specific materials, systems and methods

We require information from authors about some types of materials, experimental systems and methods used in many studies. Here, indicate whether each material, system or method listed is relevant to your study. If you are not sure if a list item applies to your research, read the appropriate section before selecting a response.

Materials & experimental systems

n/a	Involved in the study
	☒ Antibodies
☒	Eukaryotic cell lines
☒	Palaeontology and archaeology
☒	Animals and other organisms
	☒ Clinical data
☒	Dual use research of concern

Methods

n/a	Involved in the study
☒	ChIP-seq
	☒ Flow cytometry
☒	MRI-based neuroimaging

Antibodies

Antibodies used

This data is available in supplemental table 2 but we have also included it here as requested. Live/dead Aqua NA Invitrogen L34957
CD3 BB515 UCHT1 BD Bioscience 564465
CD4 BuV395 L200 BD Bioscience 564107
CD8 BuV737 SK1 BD Bioscience 612754
FoxP3 Alexa647 259D/C7 BD Bioscience 560045
GranB Alexa700 GB11 BD Bioscience 560213
IL10 BV421 JES3-9D7 BD Bioscience 566276
TNFa BV711 MAb11 BioLegend 502940
CD25 PE M-A251 BD Bioscience 560989
Ki67 PE-Cy5 SoLA15 Invitrogen 15-5698-82
IFNg PE-Cy7 4S.B3 BD Bioscience 557844
TGFb PE-CF594 TW4-9E7 BD Bioscience 562422
PD1 BB700 EH12.1 BD Bioscience 566461
T cell Panel
Live/dead Aqua NA Invitrogen L34957
CD3 BB515 UCHT1 BD Bioscience 564465
CD4 BuV395 L200 BD Bioscience 564107
CD8 BuV737 SK1 BD Bioscience 612754
CD45RO BV711 UCHL1 BD Bioscience 563723
CD19 BV605 SJ25C1 BD Bioscience 562653
CD25 PECF594 M-A251 BD Bioscience 562403
PD-1 PE MIH4 BD Bioscience 560908
CD278 BV785 C398.4A BD Bioscience 567923
Ki67 PE-Cy5 SoLA15 Invitrogen 15-5698-82
HLADR PE-Cy7 G46-6 BD Bioscience 560651
TREG / NK panel
Live/dead Aqua NA Invitrogen L34957
CD3 BB515 UCHT1 BD Bioscience 564465
CD4 BuV395 L200 BD Bioscience 564107
CD8 BuV737 SK1 BD Bioscience 612754
CD16 Alexa700 3G8 BD Bioscience 557920
CD56 PE MY31 BD Bioscience 566647
CD25 PECF594 M-A251 BD Bioscience 562403
FoxP3 Alexa647 259D/C7 BD Bioscience 560045
TIGIT BB700 741182 BD Bioscience 741182
TIM3 BV711 7D3 BD Bioscience 565566
B cell panel
Live/dead Aqua NA Invitrogen L34957
CD19 BV786 SJ25C1 BD Bioscience 563325
HLADR PE-Cy7 G46-6 BD Bioscience 560651
IgD BB700 IA6-2 BD Bioscience 566538
CD27 Alexa647 M-T271 BioLegend 366434
CD5 BV605 UCHT2 BD Bioscience 563945
CD1d PE CD1d42 BD Bioscience 550255
CD25 PECF594 M-A251 BD Bioscience 562403
PDL1 BuV395 MIH1 BD Bioscience 740320
IL10 BV421 JES3-9D7 BD Bioscience 566276
MDSC / DC panel
Live/dead Aqua NA Invitrogen L34957
CD3 BV711 OKT3 BioLegend 317328
CD19 BV711 SJ25C1 BD Bioscience 563036
CD56 BV711 B159 BD Bioscience 740781
CD14 BuV395 MϕP9 BD Bioscience 563562
CD11b PECY7 M1/70 BD Bioscience 561098

CD33 BB515 WM53 BD Bioscience 564588
HLADR BB700 G46-6 BD Bioscience 566481
CD11c BV605 B-ly6 BD Bioscience 563930
CD1c PE F10/21A3 BD Bioscience 564900
CD141 BV421 1A4 BD Bioscience 563321
CD303 BV786 V24-785 BD Bioscience 748000
PDL1 APC MIH1 BD Bioscience 563741

Validation

All anti-bodies used are commercially available and have been previously validated by the manufacturer as well as in published data.

Clinical data

Policy information about clinical studies

All manuscripts should comply with the ICMJEguidelines for publication of clinical research and a completedCONSORT checklist must be included with all submissions.

Clinical trial registration	NCT02599454
Study protocol	The full study protocol has been submitted with the supplemental information
Data collection	Data collection occurred at UC Davis Comprehensive Cancer Center, David Grant Medical Center, and Cedars Sinai Medical Center from 4/2016 - 4/2021
Outcomes	1.1 Primary Objective<br>To determine the maximum tolerated dose (MTD) of MPDL3280A that can be given with SAR in patients with inoperable early stage NSCLC.<br>1.2 Secondary Objectives<br>1.2.1 To characterize the safety profile of this regimen using CTCAE v4 (Common Toxicity Criteria for Adverse Events version 4)<br>1.2.2 To provide preliminary efficacy data of the combination as determine by objective response rate and disease free survival using RECIST 1.1 (Response Evaluation Criteria for Solid Tumors) and Immune Related RECIST (irRECIST).<br>1.3 Exploratory Objectives<br>1.3.1 To analyze serial blood for change in cytokine signatures, FACS and immunophenotyping of peripheral blood mononuclear cells (PBMCs) and tumor infiltrating immune cells.<br>1.3.2 To evaluate pre and post treatment tumor tissue (if available) for PD-L1 and other immune proteins in the tumor and tumor microenvironment and for molecular profiling in a subset of patient samples.<br>1.3.3 To discover biomarkers of response from the data obtained in 1.3.1 and 1.3.2.

Flow Cytometry

Plots

Confirm that:

- [x] The axis labels state the marker and fluorochrome used (e.g. CD4-FITC).
- [x] The axis scales are clearly visible. Include numbers along axes only for bottom left plot of group (a 'group' is an analysis of identical markers).
- [x] All plots are contour plots with outliers or pseudocolor plots.
- [x] A numerical value for number of cells or percentage (with statistics) is provided.

Methodology

Sample preparation	Cryopreserved PBMCs were thawed, washed, and viability was assessed. They were then incubated with Fc-block (BD Bioscience, 202 Franklin Lakes, NJ) on ice for 15 minutes. Then, cells were stained with specific antibody cocktails 203 (supplemental Table 2) for 1 h on ice, and then stained with Aqua-LIVE/DEAD (Invitrogen, 204 Carlsbad, CA) for 30 min at room temperature. Cells were washed after each step using PBS containing 0.5% BSA and before being analyzed.
Instrument	BD Fortessa flow cytometer (BD 206 Bioscience, Franklin Lakes, NJ).
Software	FlowJo V10.6.2
Cell population abundance	Sorting was not performed.
Gating strategy	Gating was performed using fluorescence minus one groups to set the negative gate. Gating strategies are demonstrated in representative flow plots throughout the manuscript.

- [x] Tick this box to confirm that a figure exemplifying the gating strategy is provided in the Supplementary Information.
